# New insights into the evolution of portunoid swimming crabs (Portunoidea, Heterotremata, Brachyura) and the brachyuran axial skeleton

**DOI:** 10.1186/s12983-022-00467-8

**Published:** 2022-10-27

**Authors:** Dennis Hazerli, Christoph Gert Höpel, Stefan Richter

**Affiliations:** Allgemeine & Spezielle Zoologie, Institut für Biowissenschaften, Universität Rostock, Universitätsplatz 2, 18055 Rostock, Germany

**Keywords:** Swimming crab, Brachyura, Axial skeleton, 3D reconstruction, Morphology, Character, Evolution, Phylogeny

## Abstract

Portunoidea (Heterotremata) is a morphologically disparate taxon of true crabs (Brachyura) best-known for many of its representatives being considered “swimming crabs”. The term “swimming crab”, however, sometimes refers to a distinct taxon (traditionally to Portunidae within Portunoidea), and sometimes to a certain morphotype in which the 5th pereiopod (P5) has a specific shape that facilitates swimming. We use the term “P5-swimming crab” or “P5-swimmer” herein, not only to restrict it to the morphotype, but also to distinguish the swimming in question from other kinds of swimming in Brachyura. The evolution of P5-swimming crabs has not yet been satisfactorily investigated. In particular, it is not known whether the morphotype evolved several times independently in different lineages of Portunoidea or whether it evolved only once and was lost in several lineages. Ours is the first approach combining molecular with morphological data to result in a new phylogenetic positioning of some members of Portunoidea. For the first time, data from the axial skeleton and extrinsic musculature are used. Morphological examinations reveal that the axial skeleton and extrinsic musculature in P5-swimming crabs are more diverse than previously thought, with the exception of the P5 anterior coxa muscle, which originates at the median plate in all P5-swimmers. Ancestral state reconstructions based on parsimony reveal that the stem species of Portunoidea already showed the morphotype of a P5-swimming crab, but with a long merus which probably resulted in less effective P5-swimming than in extant P5-swimming crab species with a short merus. Several other extant taxa represent a reversal of the P5-swimmer morphotype to varying degrees, with some extant species showing a complete reversal of unambiguous P5-swimming crab character states—one example being the well-known common shore crab *Carcinus maenas*. The absence of a connection between interosternite 7/8 and the sella turcica (the secondary loss of the “brachyuran sella turcica”) in the stem species of Heterotremata, resulting in a junction plate which forms a cavity that offers room and attachment sites for the P5 extrinsic musculature is uncovered as preadaptation to the P5-swimmer morphotype in Heterotremata. This preadaptation is missing in Podotremata and Thoracotremata, the other two traditional main taxa of Brachyura.

**Supplementary Information:**

The online version contains supplementary material available at 10.1186/s12983-022-00467-8.

## Introduction

Brachyuran crabs of the taxon Portunoidea Rafinesque, 1815 display a wide range of morphological disparity. The taxon is well-known for many of its representatives exhibiting a specific morphotype in which the 5th pereiopod (P5) is shaped as a swimming leg, enabling these crabs to perform a special mode of swimming (see for example [21, 29, 51, 52]). This particular mode of swimming is referred to herein as *P5-swimming* to distinguish it from other modes of swimming that have been documented in crabs that show no swimming modifications of their pereiopods or that have different morphological modifications for swimming [21, 66]. Accordingly, we refer to the morphotype as the *P5-swimming crab* (or *P5-swimmer*) *morphotype,* while referring to P5 in this morphotype as the *swimming leg*. However, many portunoid taxa do not exhibit this morphotype. For example, representatives of *Carcinus* Leach, 1814 and *Chaceon* R.B. Manning & Holthuis, 1989 are typical walkers, entirely lacking swimming ability (but being able to bury), while others like *Thia* Leach, 1815 and *Portumnus* Leach, 1814 show morphological features primarily considered to represent adaptations to a burying mode of life [2, 7, 13, 14, 21, 22, 41, 51]. Some representatives of the portunid subfamily Thalamitinae Paulson, 1875 are known to live in symbiotic relationships to other marine organisms, and in some of these cases the P5 is modified for grasping [6, 12, 20, 62, 65].

The morphology and evolution of portunoid crabs has been little examined considering the high level of disparity in this taxon. The external features of the P5-swimming crab morphotype/swimming leg were characterized by Hartnoll [21] on the basis of statements by Herter [24] regarding *Liocarcinus holsatus* (J. C. Fabricius, 1798). They include (1) a significantly shorter P5 merus than in a walking leg, (2) a P5 propodus and dactylus that are much broader than in a walking leg, and paddle-like, (3) a rotation in the P5 thoracal-coxal articulation axis of about 90° compared to a hypothetical ancestor (i.e. a subdorsal P5 coxa position) and (4) an increased range of motion in the P5 coxal-basi-ischial and meral-carpal articulations. According to Kühl [29], the external features of the P5-swimming crab morphotype/swimming leg also include (5) a carpal-propodal articulation axis that lies on the longitudinal propodus axis (rather than oblique to it like in a walking leg; see also [22]). Schäfer [51] emphasizes the significance of (6) relatively long setae arranged in dense fringes along P5 podomere margins for P5-swimming. However, whether a short merus really should be used to characterize a P5-swimmer is a matter of debate. Steudel [66] found several portunoid genera to exhibit all the typical P5-swimming crab features mentioned above except for a short merus. One of them—*Carupa* Dana, 1851—was anecdotally reported to swim, but with no details given on the swimming technique. It was concluded that these genera are generally able to perform the same swimming movements as “real” P5-swimmers, but not as fast or effectively [66]. However, this hypothesis was never corroborated by behavioural observations or kinematic studies. Another feature considered to be crucial to the swimming performance of a P5-swimming crab is the ability to bend the swimming leg in an antero-dorsal direction over the carapace [21, 29, 51, 52, 66].

When it comes to internal anatomy, there is a distinct lack of information about most taxa of Portunoidea, as there is about other Brachyura. Of particular interest are features concerning the inner skeleton, which is formed by infoldings of the exoskeleton [9, 19, 56–58]. In the P5-swimmer *Liocarcinus depurator* (Linnaeus, 1758) (which is morphologically very similar to *L. holsatus*) the endophragmal system shows significant differences to that in non-swimmers such as *Cancer pagurus* Linnaeus, 1758 and *Carcinus maenas* (Linnaeus, 1758) [22]. These differences are associated with the enlarged P5 extrinsic musculature in P5-swimmers, and with differences in muscle attachment sites. Generally speaking, it is a combination of these inner morphological traits and external features (particularly those of the swimming leg) that permit P5-swimming and that can thus be used to characterise the P5-swimming crab morphotype [22]. Schmidt et al. [52] also found an increased range of motion in the P5 thoracal-coxal articulation in P5-swimmers, which matches findings by Hazerli and Richter [22] in which a larger P5 thoracal-coxal arthrodial cavity is present in *L. depurator* than in *C. maenas* and *C. pagurus*. However, the latter finding has yet to be confirmed in other P5-swimmers, as do features concerning the endophragmal system and extrinsic musculature. The first aim of this study, therefore, is to examine, describe and 3D-visualize the axial skeleton (= endophragmal system + pleurum + sternum; for more information on the terminology used here, see [22]) and P5 extrinsic musculature of various portunoid taxa (together with some outgroup taxa). This study is the most comprehensive to date on these structures in Portunoidea, and the first to test whether the traits (morphemes) found in *L. depurator* are also present in other P5-swimmers.

The main goal of the present study is to deduce the transformations involved in morphological character evolution in Portunoidea, and to test whether the P5-swimmer morphotype evolved several times independently or whether it appeared only once and was lost in some lineages. To answer this question, a robust phylogenetic hypothesis is necessary, which to date has not been achieved. Previous phylogenetic analyses of Portunoidea remain controversial, at least in part [12, 26, 54, 63, 64]. In the present study, a new phylogenetic hypothesis is formulated by combining available gene sequences with new morphological data pertaining to the inner anatomy and newly conceptualized characters of external structures (for the most part based on Karasawa et al. [26], though statements by Evans [12] and Spiridonov [64] are also considered in our character conceptualization). To understand the evolution of morphological traits, the ancestral states of characters are reconstructed on the basis of parsimony [49, 55, 67].

## Material and methods

### Taxon sampling, provision of voucher material

Several species of Portunoidea were chosen to represent the ingroup (Table 1). The taxon sampling represents the morphological disparity (especially with regard to the locomotive apparatus, which involves pereiopods 2–5) in putative monophyletic groups within Portunoida (*sensu* [12]). Typical P5-swimming crabs are represented in each group. Based on the criteria mentioned by Herter [24], Kühl [29], Schäfer [51], and Hartnoll [21], typical P5-swimmers are *Liocarcinus depurator, “Polybius” henslowii* Leach, 1820 (we here follow [12] in putting *Polybius* in quotation marks as the genus has repeatedly been recognized to be nested within *Liocarcinus* Stimpson, 1871, which is then paraphyletic; see also [40, 54]),* Macropipus rugosus* (Doflein, 1904), *Necora puber* (Linnaeus, 1767) and *Parathranites orientalis* (Miers, 1886) (all six Carcinidae MacLeay, 1838 *sensu* [12]), *Ovalipes ocellatus* (Herbst, 1799) (Geryonidae Colosi, 1923), *Callinectes sapidus* Rathbun, 1896 and *Portunus inaequalis* (Miers, 1881) (both Portunidae Rafinesque 1815; *Portunus* has recently been shown to be paraphyletic [34]; The recent suggestion to transfer *Portunus inaequalis* into a genus *Achelous* came too late to be fully considered herein [28]). Outgroup taxa are *Sternodromia monodi* (Forest & Guinot, 1966) representing Dromiidae De Haan, 1833 of Podotremata Guinot, 1977 (which may be paraphyletic, see for example Luque et al. [32]), *Eriochier sinensis* H. Milne Edwards, 1853 and *Varuna litterata* (J. C. Fabricius, 1798) as representatives of Thoracotremata Guinot, 1977, the putative sister taxon to Heterotremata Guinot, 1977 (to which Portunoidea belong), and *Medorippe lanata* (Linnaeus, 1767) (Dorippidae MacLeay, 1838, Dorippoidea MacLeay, 1838) as putative basal heterotrematan species [9, 10, 19, 25]). Several other non-portunoid taxa of Heterotremata were also added to represent the outgroup, with *Cancer irroratus* Say, 1817 and *Cancer pagurus* being chosen as representatives of Cancroidea Latreille, 1802, and *Corystes cassivelaunus* (Pennant, 1777) and *Telmessus cheiragonus* (Tilesius, 1815) being chosen to represent Corystoidea Samouelle, 1819, both putative sister taxa to Portunoidea [54]. *Ashtoret lunaris* (Forskål, 1775) (Matutidae De Haan, 1835) was added as having comprehensive morphological modifications for swimming and/or burying but not fitting all the criteria proposed by earlier authors for a P5-swimmer. *Calappa granulata* (Linnaeus, 1758) represents Calappidae De Haan, 1833, the potential sister taxon to Matutidae (both Calappoidea De Haan, 1833) [27, 31, 38]. Altogether, representatives of 34 species were examined.

**Table 1:** Taxa used in this study with their voucher codes, references and GenBank accession numbers

Taxa examined for morphological chracters	Voucher ID (morphological characters)	Taxa examined and combined as OTUs for the genetic data set	Voucher ID (genetic data set)	References	16S	CO1	NADH1	H3
*Ashtoret lunaris*	SMF 19731, ZSRO no ID	*Ashtoret lunaris*	Unknown	Tan et al. [69]	LK391941	LK391941	LK391941	
		*Matuta planipes*	ZRC2009.0753	Tsang et al. [70]				KJ133142
*Bathynectes maravigna*	ZMH-K 34625 & 34205	*Bathynectes maravigna*	MNHN-B31441	Schubart and Reuschel [54]	FM208770		FM208770	FM208814
			JSDUKdeep_47	da Silva et al. [8]		JQ305964		
*Calappa granulata*	ZSRO 347	*Calappa granulata*	ZMS:6832	Unpublished	KU206591			KU206702
			JSDAz218	da Silva et al. [8]		JQ306054		
		*Calappa bilineata*	Unknown	Lu et al. [31]			MN562587	
*Callinectes sapidus*	ZMH-K 2217 & 2218	*Callinectes sapidus*	Unknown	Place et al. [39]	AY363392	AY363392	AY363392	
			ULLZ3895	Schubart and Reuschel [54]				FM208798
*Cancer pagurus*	ZSRO no ID	*Cancer pagurus*	SMF-32764	Schubart and Reuschel [54]	FM207653		FM207653	FM208806
			JSDUK10	da Silva et al. [8]		JQ306000		
*Cancer irroratus*	ZMH-K 656	*Cancer irroratus*	ULLZ 3843	Schubart and Reuschel [54]	FM207654			FM208807
			L195AR2-01	Radulovici et al. [42]		FJ581562		
*Caphyra loevis*	AMS P.17124, SMF 6353	*Caphyra loevis*	NMMBCD 4090	Evans [12]	KT365592	KT365697		KT425009
*Caphyra rotundifrons*	ZMH-K 2565 & 2566	*Caphyra rotundifrons*	UF:4079	Evans [12]	KT365530	KT365698	KT365530	KT424989
*Carcinus maenas*	ZSRO no ID	*Carcinus maenas*	SMF-32757	Schubart and Reuschel [54]	FM208763		FM208763	FM208811
			L174AR1-07	Radulovici et al. [42]		FJ581597		
*Carupa tenuipes*	SMF ZMG 832	*Carupa tenuipes*	MNHN-B31436	Schubart and Reuschel [54]	FM208758		FM208758	FM208789
			UF16184	Evans [12]		KT365703		
*Catoptrus nitidus*	ZMH-K 3136	*Catoptrus nitidus*	MNHN-B31435	Schubart and Reuschel [54]	FM208755		FM208755	
		*Catoptrus aff. nitidus*	UF1024	Evans [12]		KT365706		
*Chaecon mediterraneus*	SMF 29486	*Chaceon granulatus*	SMF-32762	Schubart and Reuschel [54]	FM208775		FM208775	FM208827
			Unknown	Unpublished		AB769383		
*Coelocarcinus foliatus*	UF 050654	*Coelocarcinus foliatus*	UF: 40056	Evans [12]	KT365601	KT365724		KT425058
		*Coelocarcinus sp.*	UF 27553	Evans [12]			KT365545	
*Corystes cassivelaunus*	ZMH-K 4887 & 27128	*Corystes cassivelaunus*	SMF-32770	Schubart and Reuschel [54]	FM208781		FM208781	FM208801
			JSDUK23	da Silva et al. [8]		JQ306005		
*Eriocheir sinensis*	ZMH-K 24504	*Eriocheir sinensis*	Unknown	Li et al. [30]	KP064329	KP064329	KP064329	
		*Eriocheir japonica*	MSLKHC-EjapHK	Tsang et al. [70]				KJ133099
*Libystes nitidus*	ZMH-K 3143	*Libystes nitidus*	MNHN-B31438	Schubart and Reuschel [54]	FM208762		FM208762	
			UF12587	Evans [12]		KT365728		
*Liocarcinus depurator*	ZSRO no ID	*Liocarcinus depurator*	MNHN-B31439	Schubart and Reuschel [54]	FM208767		FM208767	FM208819
			JSDUK052-08	da Silva et al. [8]		JQ306013		
*Liocarcinus navigator*	ZSRO no ID, SMF 10662 & 45835	*Liocarcinus navigator*	SMF-32775	Schubart and Reuschel [54]				FM208821
			SMF<DEU>:44087	Plagge et al. [40]	KU560476	KP795939	KU560476	
*Lissocarcinus orbicularis*	SMF 19738	*Lissocarcinus orbicularis*	Unknown	Schubart and Reuschel [54]	FM208757		FM208757	FM208791
			UF15741	Evans [12]		KT365732		
*Macropipus rugosus*	ZSRO 262	*Macropipus tuberculatus*	MNHN-B31440	Schubart and Reuschel [54]	FM208769		FM208769	FM208815
			FCFOPC041-33	da Silva et al. [8]		JQ306218		
*Medorippe lanata*	ZSRO 189	*Medorippe lanata*	ZRC	Sin et al. [60]	EU636950	EU636981		
		*Dorippe quadridens*	ZRC2008.0064	Tsang et al. [70]				KJ133093
*Necora puber*	ZSRO no ID, SMF 4906	*Necora puber*	SMF-32749	Schubart and Reuschel [54]	FM208771		FM208771	FM208813
			Unknown	Sotelo et al. [61]		FJ755619		
*Ovalipes ocellatus*	SMF 22191 & 7326	*Ovalipes punctatus*	MNHN-B31442	Schubart and Reuschel [54]				FM208824
			FKU63_mgp01	Unpublished	MH802052	MH802052	MH802052	
*Parathranites orientalis*	SMF 30810	*Parathranites orientalis*	NTOU B00090	Tsang et al. [70]	KJ132616			KJ133173
*Pirimela denticulata*	ZMH-K 6780 & 6781	*Pirimela denticulata*	SMF-32767	Schubart and Reuschel [54]	FM208783		FM208783	FM208808
*Polybius henslowii*	ZMH-K 26238 & 2631	*Polybius henslowii*	SMF-32759	Schubart and Reuschel [54]	FM208765		FM208765	FM208816
			JSDUK74	da Silva et al. [8]		JQ306293		
*Portumnus latipes*	ZSRO 195, SMF 43553, ZMH-K 2616	*Portumnus latipes*	SMF-32758	Schubart and Reuschel [54]	FM208764		FM208764	FM208812
*Portunus inaequalis*	ZSRO 193 & 225	*Portunus inaequalis*	SMF-32754	Schubart and Reuschel [54]	FM208752		FM208752	FM208795
		*Portunus pelagicus*	Unknown	Meng et al. [35]		KR153996		
*Raymanninus schmitti*	UF 9676	*Raymanninus schmitti*	UF:9676	Evans [12]	KT365560		KT365560	
*Sternodromia monodi*	ZSRO 354	*Lauridromia dehaani*	AMSP67928	Ahyong and O'Meally [1]	AY583899			
			Unknown	Sin et al. [60]		EU636986		
			NTOU B00006	Tsang et al. [70]				KJ133125
			C20191207LD	Yang et al. [75]			MT038417	
*Telmessus cheiragonus*	ZMH-K 4952	*Telmessus cheiragonus*	SMF-22475	Schubart and Reuschel [54]	FM207656		FM207656	FM208802
			CIB213	Castelin et al. [5]		KX039796		
*Thia scutellata*	ZSRO 363	*Thia scutellata*	SMF-32769	Schubart and Reuschel [54]	FM208782		FM208782	FM208810
			MT04558	Raupach et al. [45]		KT209396		
*Varuna litterata*	SMF-10002, ZSRO 100	*Varuna litterata*	Unknown	Wang et al. [73]	MW125542	MW125542	MW125542	
			MZUF:2503	Schubart and Cuesta [53]				FN434060
*Xaiva biguttata*	ZMH-K 29763	No data						

Specimens used in morphological examinations were provided by the collections of the Senckenberg Research Institute and Natural History Museum in Frankfurt, Germany (SMF), the Zoological Museum of the Center of Natural History (CeNak) in Hamburg, Germany (ZMH-K), the Florida Museum of Natural History, University of Florida in Gainesville, USA (UF), the Australian Museum in Sydney, Australia (AMS) and our own collection at the Institute of Zoology in Rostock (ZSRO), Germany. For more information on the material, see Table 1. Specimens of *C. pagurus*, *Carcinus maenas*, *L. depurator* and *Liocarcinus navigator* freshly collected in 2019 from the waters near Gullmarn, Sweden, were also considered in the morphological examinations.

### Micro-computed tomography (μCT), 3D reconstruction

At least one female and one male specimen per species (if available) were used for X-ray imaging using a XRadia Versa 410 X-ray microscope (ZEISS, Oberkochen, Germany) and the program Scout and Scan v.11 (Table 1). In species in which only one sex was represented in the voucher material, at least two specimens of the same sex (if available) were used. All specimens were adults except for one juvenile specimen of *Necora puber* and immature specimens of *Callinectes sapidus* and *Portunus inaequalis*. If extrinsic pereiopod musculature was barely visible in μCT scan images, the respective specimen was bathed in alcoholic Lugol's iodine solution for several days to improve the visibility of the musculature during μCT scans. Digital image stacks obtained via μCT were processed using the 3D reconstruction software Amira 6.4/6.5/6.6/6.7 (by FEI). Scans for 3D reconstruction of the axial skeletons and P5 extrinsic musculature were chosen on the basis of the quality of the scan and the condition of the extrinsic musculature. μCT scan image stacks were checked to see whether differences in the extrinsic musculature of pereiopods 2–4 (P2–P4) could be found between species. Some of these muscles were reconstructed in 3D models as well. The remaining scans were used to check whether there were differences between specimens of the same sex or between specimens of different sexes (if available) in each species (and optionally, the axial skeleton and extrinsic musculature were roughly reconstructed). Some μCT scans, 3D models and photographs of the specimens were used to produce images and drawings using CorelDraw 2020 and common graphics programs.

### Terminology

The morphological terminology used here is basically the same as in Hazerli and Richter [22], with some adjustments pertaining to the details of the term endophragma (pl. endophragmata). In Hazerli and Richter [22], we adopt the definition of endophragma used by Guinot et al. [19] as infoldings of the axial skeleton “composed of two layers that are confluent with their cuticle at its outer margin” (and “technically inseparable at moult”). However, we found that in many species, large parts of axial skeleton infoldings generally defined as endophragmata consist of two separate, clearly distinguishable layers of cuticular infoldings. This applies in particular to large areas of the junction plate and median plate. In fact, the wall of the junction plate cavity always appears to be formed by only one layer of cuticular infolding. Plus, in many of the species in which interosternite 4/5, 5/6, and/or 6/7 is connected to the median plate, the two layers of median infoldings connected to interosternites are predominantly not confluent at their outer margin. These infoldings are not true endophragmata according to the definition used by Hazerli and Richter [22]. However, here we use a less strict definition of the term endophragma, simply labelling the median plate and interosternites as median and lateral sternal infoldings, and interopleurites as pleural infoldings, which together with the junction plate and sella turcica are referred to as endophragmata herein.

### Molecular data set, phylogenetic analysis and ancestral state reconstruction

We created a combined dataset containing 2141 characters comprised of 59 newly conceptualised morphological characters and 2082 nucleotide sites. All characters were equally weighted. Gaps were treated as missing data. In all analyses, *Sternodromia monodi* was set as the most basal outgroup. Sequences were obtained from GenBank and derived from 63 vouchered specimens listed in Table 1. The final molecular data set comprises 33 operational taxonomic units (OTUs). When sequence data were not available for a certain species analysed for morphology, OTUs were concatenated of the species studied morphologically and sequence data of closely related species (see Table 1), an approach which has been used previously (e.g. [15]). This data set combines fragments of 16S rRNA, CO1, NADH1, and H3 data from 90 sequences. The sequences of all four gene fragments were separately aligned using Geneious Alignment with default parameters (16S, COI, H3) or with the parameters Gap open penalty and Gap extension penalty set to 30 (NADH1) in Geneious Prime 2021.0.3 (Biomatters Limited) and concatenated to a single molecular data set with a total number of 2082 nucleotides (16S: 671 Bp, COI: 657 Bp, NADH1: 426 Bp, H3: 328 Bp). Published sequences were mostly drawn from Schubart and Reuschel [54], da Silva et al. [8] and Evans [12]. The dataset was analysed using maximum parsimony (MP) and Bayesian inference (BI). For BI analyses, the best fit model of evolution was determined using the implemented model test in MEGA X. BI analyses were performed using MrBayes 3.2.7a [50] on CIPRES Science Gateway [36]. The GTR + G + I Model was applied. Each Bayesian analysis included four runs with four differentially heated chains and the analysis was run for 30 × 10^6 generations, sampled every 3000 generations. The first 10% were discarded as burn-in. All parameters were checked with Tracer version 1.7 [44]. The MP analyses were performed in TNT v1.5. [16] using New Technology. In “New Technology Search” the following parameters were used: initial addseq = 30, find minimum tree length 100 times and random seed = 2000. Default settings were used for sectorial searches, tree drifting and tree fusing. Unsupported nodes were collapsed. Nodal support was assessed using 1000 replicates of standard bootstrap support (“New Technology Search” with sectorial search, ratchet, drift and tree fusing [default], initial addseqs = 30, find minimum length tree 2 times). The resulting trees were visualized using FigTree v.1.4.2 [43] and Corel Draw 2020.

Ancestral state reconstructions were traced using the software Mesquite (build 927) by Maddison and Maddison [33]. They were based on the phylogenetic tree deduced from MP analysis by choosing the parsimony model ‘unordered’ as the most conservative option for optimizing character states.

## Results and discussion of morphological characters

### Character conceptualization

We here follow Hennig ([23]; see also [17, 18, 48, 74]) in assigning character states belonging to a transformation series (which in turn represents the character). We further treat the assignment of the same state to different taxa as “character state identity hypothesis”, while the assignment of two (or more) different states to the same character represents one (or more) “transformational hypothesis” (or “hypotheses”). Both types of hypotheses can be seen as homology hypotheses of a kind (e.g., [23, 47, 68, 72]). Characters are phrased as suggested by Sereno [59]. Character dependencies are expressed by “inapplicable (–) if” followed by the state(s) of the character(s) which make(s) the character in question inapplicable for a certain taxon. This work focuses on characters concerned with the axial skeleton, the extrinsic musculature of pereiopods 2–5 (P2–P5) and the external morphology of the 5th pereiopod (the extensively modified swimming leg in P5-swimmers), which are discussed in detail herein. With respect to remaining external features, characters are only briefly discussed and conceptualized mainly on the basis of statements by Karasawa et al. [26] and Spiridonov [64]. A complete character state data matrix is suppilied in the “Appendix” in Additional file 1.

#### Characters concerned with the axial skeleton and extrinsic musculature of pereiopods 2–5

**Fig. 1:**
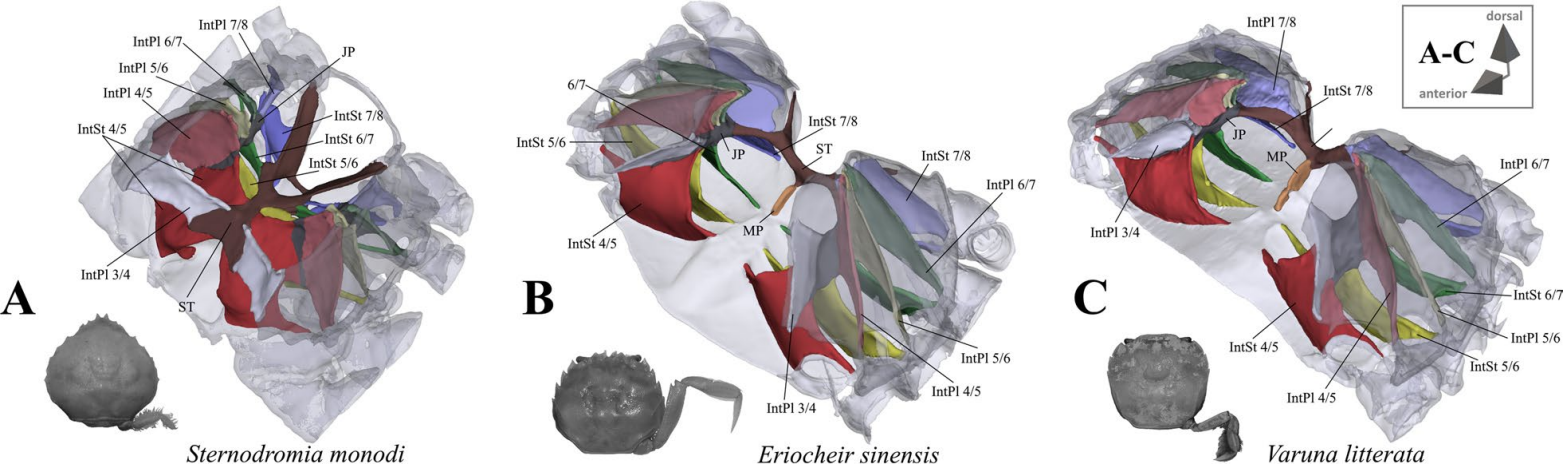
Representations of three-dimensional (3D) data from the axial skeletons of outgroup taxa of Podotremata and Thoracotremata. **A** Dromiidae (Podotremata), 3D model is supplied in Additional file 2, **B**, **C** Varunidae (Thoracotremata), 3D models are supplied in Additional files 3, 4. Each taxon is also represented by an image showing the cephalothorax with right 5th pereiopod in dorsal view. *IntPl* interopleurite (with number pair indicating thoracomeres between which it is situated), *IntSt* interosternite (with number pair indicating thoracomeres between which it is situated), *JP* junction plate, *ST* sella turcica

**Fig. 2:**
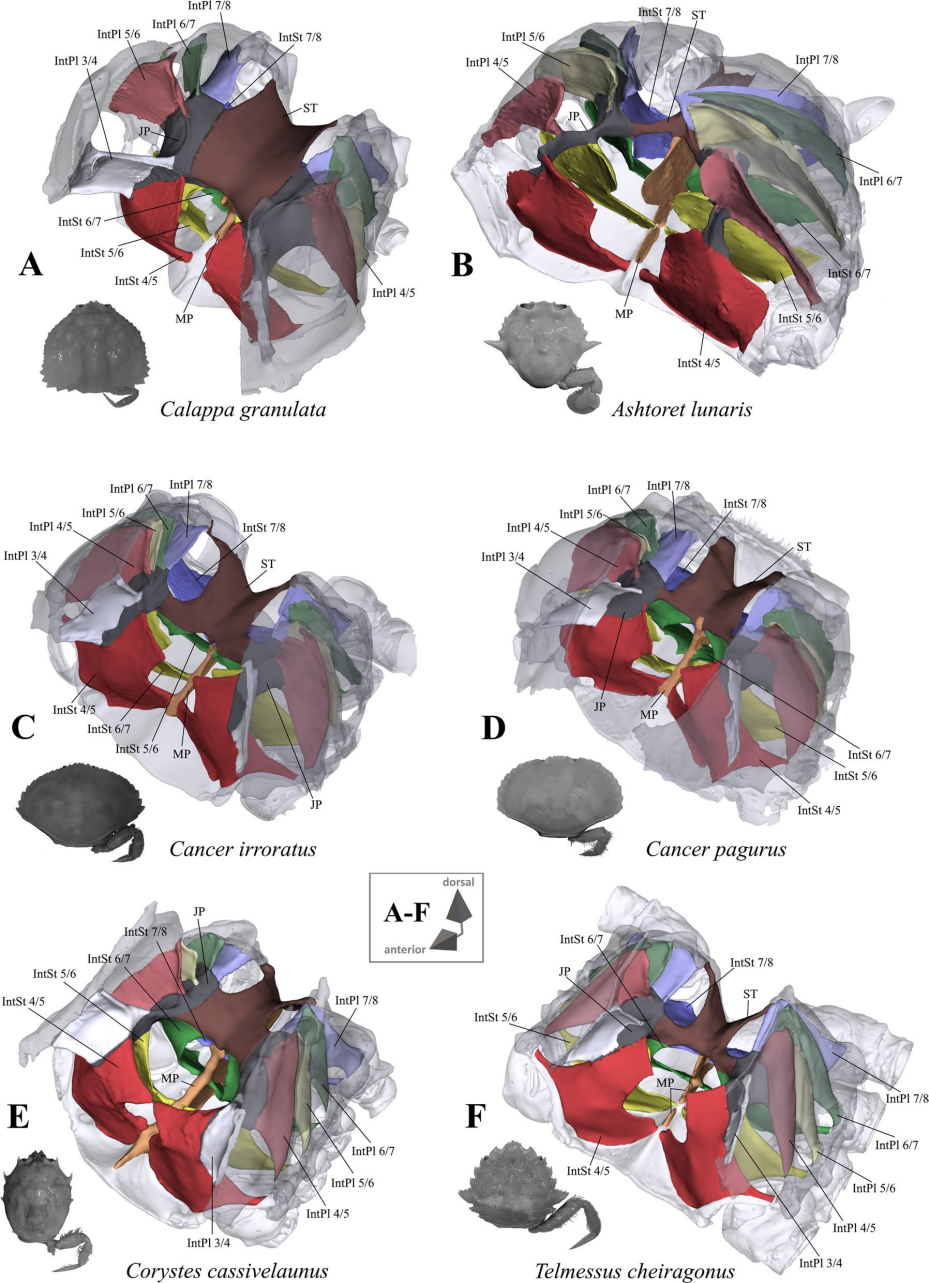
Representations of three-dimensional (3D) data from the axial skeletons of outgroup taxa of Heterotremata. **A** Calappidae (Calappoidea), 3D model is supplied in Additional file 5, **B** Matutidae (Calappoidea), 3D model is supplied in Additional file 6, **C**, **D** Cancridae (Cancroidea), 3D models are supplied in Additional files 7, 8, **E** Corystidae (Corystoidea), 3D model is supplied in Additional file 9, **F** Cheiragonidae (Corystoidea), 3D model is supplied in Additional file 10. Each taxon is also represented by an image showing the cephalothorax with right 5th pereiopod in dorsal view. *IntPl* interopleurite (with number pair indicating thoracomeres between which it is situated), *IntSt* interosternite (with number pair indicating thoracomeres between which it is situated), *JP* junction plate, *ST* sella turcica

**Fig. 3:**
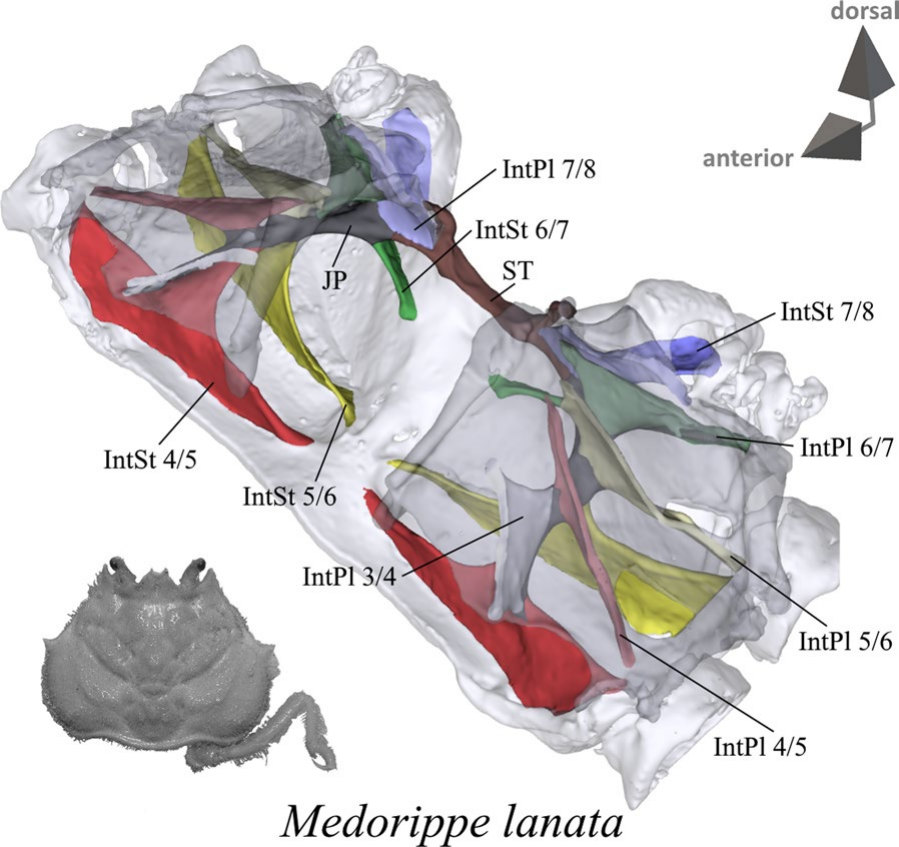
Representations of three-dimensional (3D) data from the axial skeleton of outgroup taxon *Medorippe lanata*. The species is also represented by an image showing the cephalothorax with right 5th pereiopod in dorsal view. 3D model is supplied in Additional file 11. *IntPl* interopleurite (with number pair indicating thoracomeres between which it is situated), *IntSt* Interosternite (with number pair indicating thoracomeres between which it is situated), *JP* junction plate, *ST* sella turcica

**Fig. 4:**
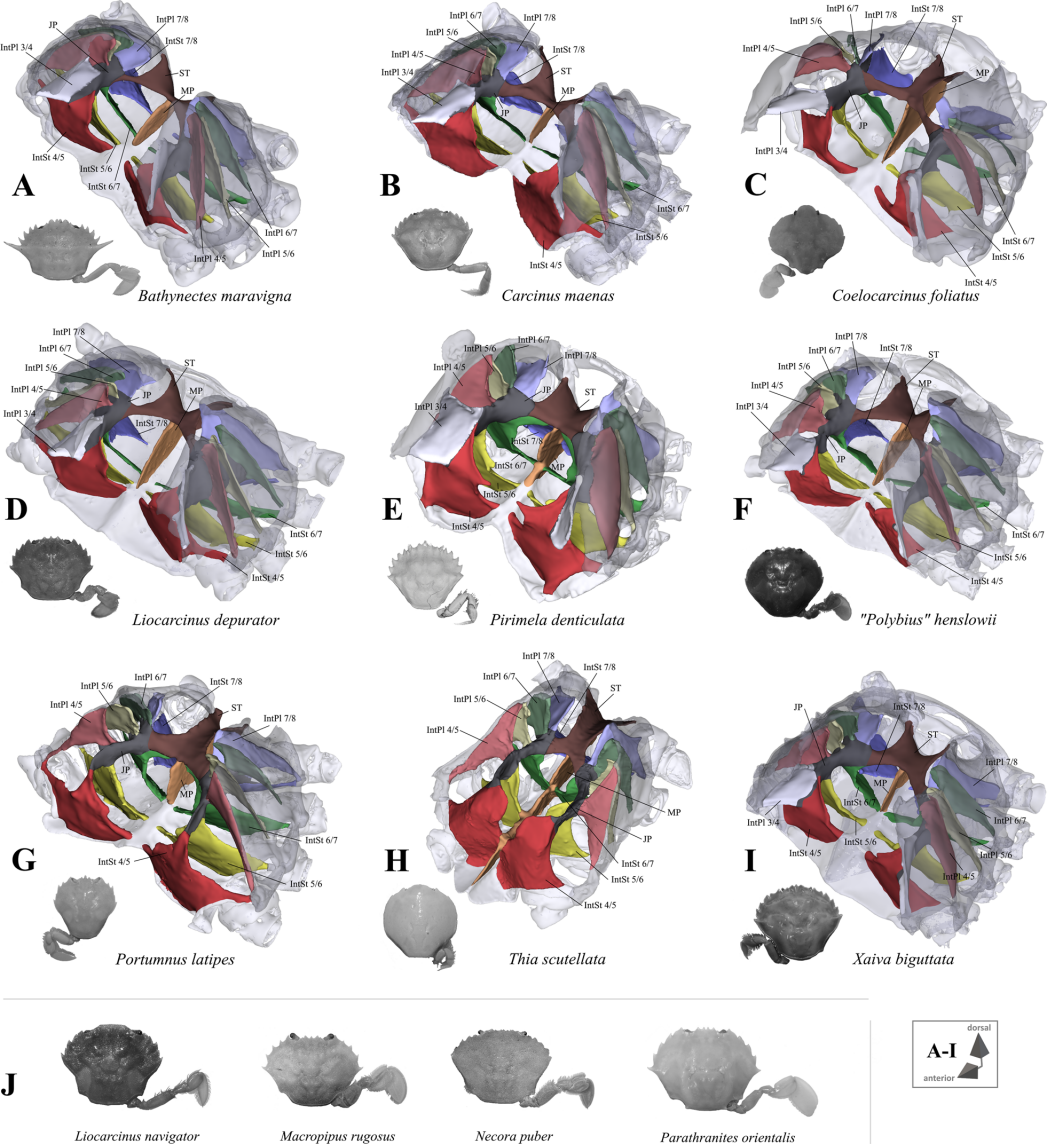
Portunoid taxa assigned to Carcinidae on the basis of Evans [12]. **A**–**I** Representations of three-dimensional (3D) data from the axial skeletons together with an image of each species showing the cephalothorax with right or left 5th pereiopod in dorsal view, 3D models are supplied in Additional files 12, 13, 14, 15, 16, 17, 18, 19, 20, **J** images (showing the cephalothorax with right 5th pereiopod in dorsal view) of species in which the axial skeleton was examined in this study but without a complete three-dimensional (3D) model being created. *IntPl* interopleurite (with number pair indicating thoracomeres between which it is situated), *IntSt* interosternite (with number pair indicating thoracomeres between which it is situated), *JP* junction plate, *ST* sella turcica

**Fig. 5:**
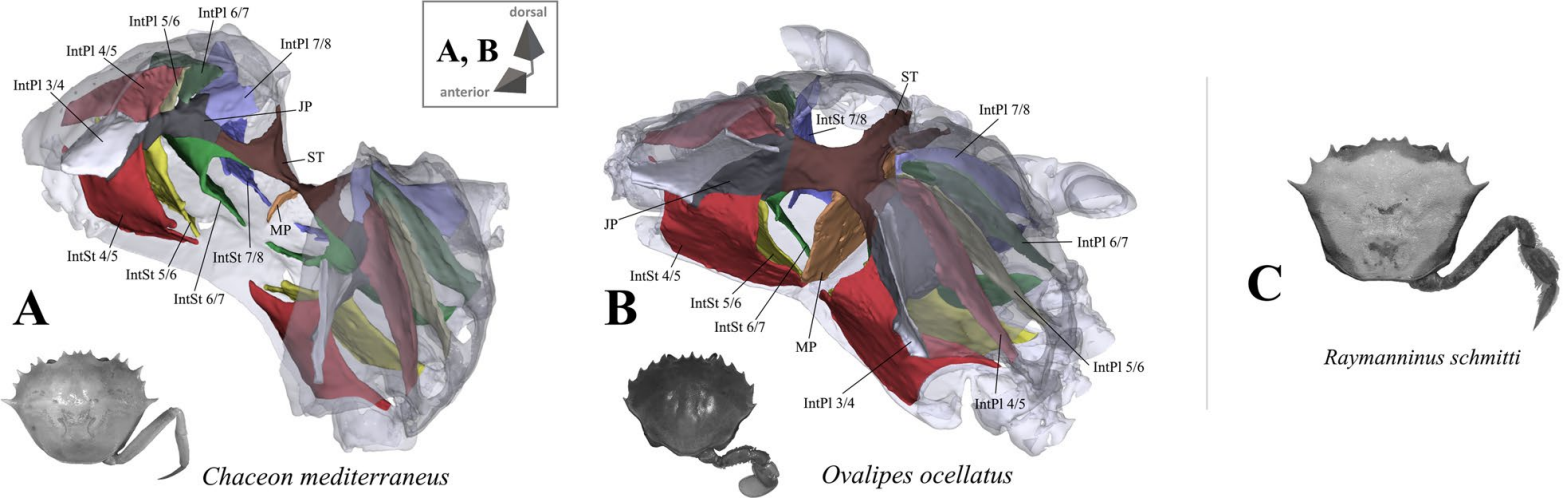
Portunoid taxa assigned to Geryonidae on the basis of Evans [12]. **A**, **B** Representations of three-dimensional (3D) data from the axial skeletons together with an image of each species showing the cephalothorax with right 5th pereiopod in dorsal view, 3D models are supplied in Additional files 21, 22, **C** image of *Raymanninus schmitti* (showing the cephalothorax with right 5th pereiopod in dorsal view). The axial skeleton of this species was examined in this study but without a complete three-dimensional (3D) model being created. *IntPl* interopleurite (with number pair indicating thoracomeres between which it is situated), *IntSt* interosternite (with number pair indicating thoracomeres between which it is situated), *JP* junction plate, *ST* sella turcica

**Fig. 6:**
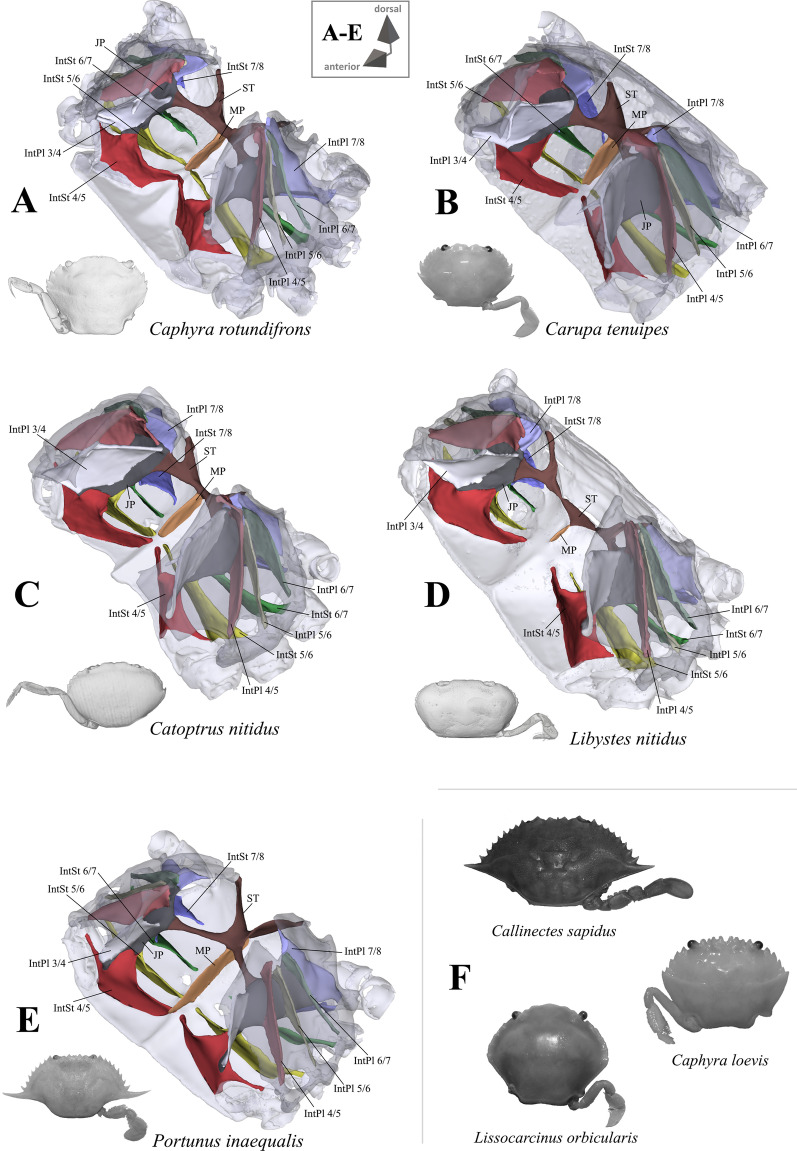
Portunoid taxa assigned to Portunidae on the basis of Evans [12]. **A**–**E** Representations of three-dimensional (3D) data from the axial skeletons together with an image of each species showing the cephalothorax with right or left 5th pereiopod in dorsal view, 3D models are supplied in Additional files 23, 24, 25, 26, 27, **F** images (showing the cephalothorax with right 5th pereiopod in dorsal view) of species in which the axial skeleton was examined in this study but without a complete three-dimensional (3D) model being created. *IntPl* interopleurite (with number pair indicating thoracomeres between which it is situated), *IntSt* interosternite (with number pair indicating thoracomeres between which it is situated), *JP* junction plate, *ST* sella turcica

**Fig. 7:**
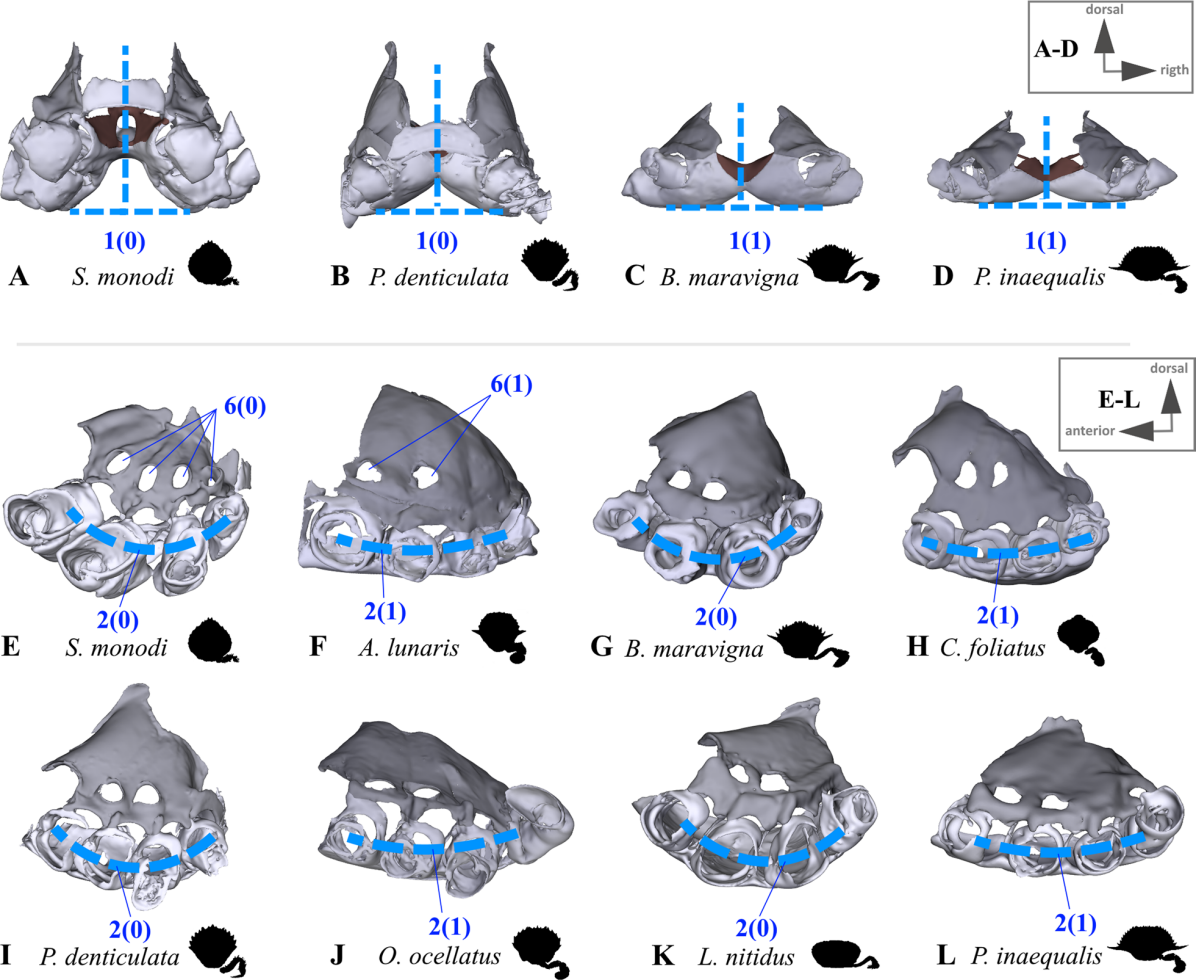
Examples showing character states concerned with axial skeleton shape and axial skeleton proportion. **A**–**D** Posterior view indicating sternum width in relation to axial skeleton height (character 1), **E**–**L** lateral view indicating degree of sternum curving (character 2) and number of gill openings (character 6)

**Fig. 8:**
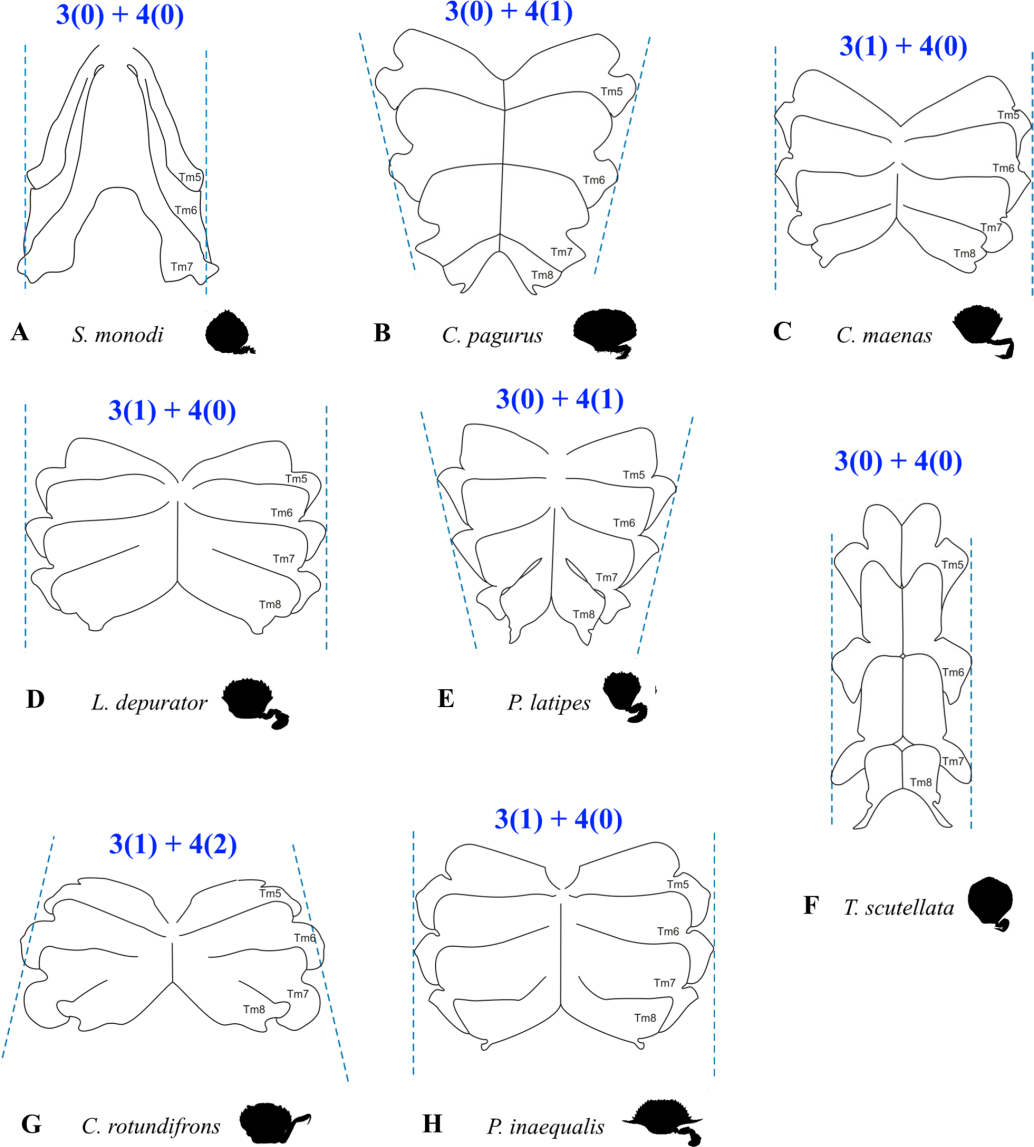
Drawings showing variability in sternum shape seen from ventral, with character states concerned with sternum proportion (character 3) and sternum shape (character 4). **A** Dromiidae (Podotremata), **B** Cancridae (Cancroidea), **C-F** Carcinidae (Portunoidea), **G, H** Portunidae (Portunoidea). *Tm* thoracomere (with number)

**Fig. 9:**
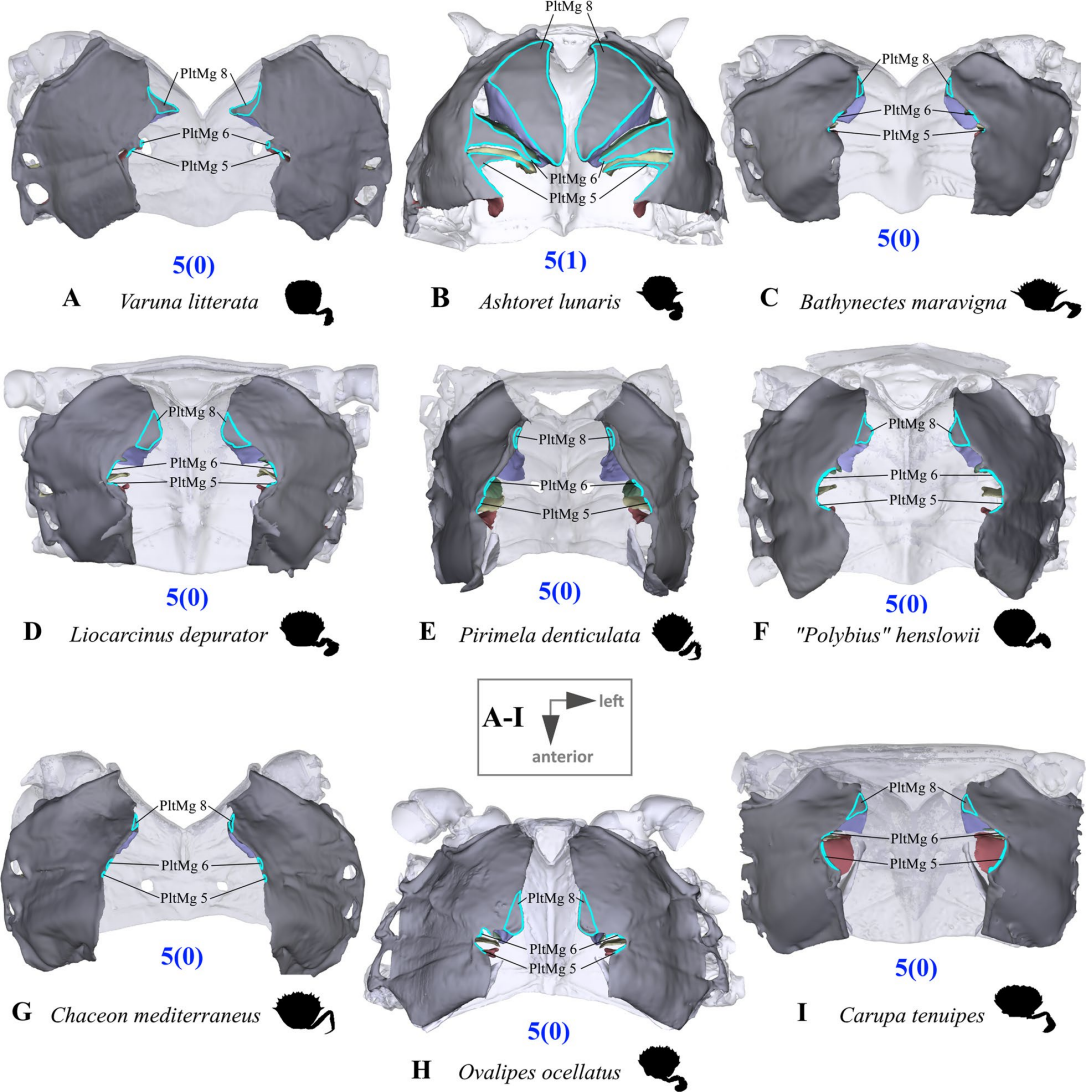
Examples showing variability in the pleural medial margins of thoracomeres 5–6 and 8 (character 5) seen from drosal. Note the prominent pleural expansions in *Ashtoret lunaris* (**B**). **A** Varunidae (Thoracotremata), **B** Matutidae (Calappoidea), **C-F** Carcinidae (Portunoidea), **G, H** Geryonidae (Portunoidea), **I** Portunidae (Portunoidea). *PltMg* pleural medial margin (with number indicating thoracomere)

*Shape and proportion of overall axial skeleton* An overview of axial skeleton morphology in most of the taxa is given in Figs. 1, 2, 3, 4, 5 and 6. High resolution 3D models of these species’ axial skeletons including some of the extrinsic musculature of the pereiopods are supplied in the “Appendix” in Additional files 2, 3, 4, 5, 6, 7, 8, 9, 10, 11, 12, 13, 14, 15, 16, 17, 18, 19, 20, 21, 22, 23, 24, 25, 26, 27. Low resolution 3D models of some of the species’ axial skeletons (*Medorippe lanata*, “*Polybius*” *henslowii*, *Thia scutellata*, *Ovalipes ocellatus*, *Libystes nitidus*) without extrinsic musculature are supplied in the “Appendix” in Additional files 28, 29, 30, 31, 32. No apparent differences in the shape of the axial skeleton or the extrinsic musculature of the pereiopods are found between sexes, except in *Medorippe lanata* (see below). No distinct differences were found between a juvenile and adult specimen of *Necora puber* either. The axial skeleton in all taxa consists of a ventral sternum, a left and right latero-dorsal pleurum, and infoldings deriving from them. Interosternites and interopleurites are, respectively, pairwise sternal and pleural infoldings between thoracomeres. The interosternites and interopleurites of each lateral body side are fused to form the two junction plates of the endophragmal system, the two lateral halves of which are connected by the sella turcica, which also seperates the cephalothorax from the pleon. Most species have a median plate, which is a medially uprising endophragma of the sternum (for more information on the terminology of morphemes, see [22]). Examples of the states concerned with axial skeleton proportions (character 1) are shown in Fig. 7A–D. The degree of dorsoventral sternal curving, which is conceptualized as two discrete states herein (character 2), can be seen in Fig. 7E–L. Characters concerned with sternum proportions (character 3) and shape (character 4) are shown in Fig. 8. Characters 1–3 are conceptualized with respect to thoracomeres 5–8 only, since the shape of thoracomere 4 depends on the size of the cheliped, which is not included here. *Ashtoret lunaris* is the only taxon in which the pleurites of thoracomeres 5, 6 and 8 expand noticeably in a medial direction, with interopleurites (= infoldings of the pleurum) 5/6 and 6/7 almost reaching the median plane of the axial skeleton. Although this is restricted to a single taxon, we conceptualize this as a character here (character state 5(1); Fig. 9). A minor medial expansion of pleurite 8 is also present in P5-swimming crabs (Fig. 9D, F, H). However, we do not conceptualize this as a character state here since variation in this morphological feature is very high between species, with a more or less distinct medial expansion of pleurite 8 also being present in several non-P5-swimmers (Fig. 9A, G).

1. (Fig. 7A–D) Thoracomeres 5–8, axial skeleton, maximum height relative to maximum width: higher than wide or about as high as wide (0); wider than high (1).
2. (Fig. 7E–L) Thoracomeres 5–8, sternum, dorsoventral curving: distinct (0); indistinct (1).
3. (Fig. 8) Thoracomeres 5–8, sternum, maximum length relative to maximum width: longer than wide (0); wider than long (1).
4. (Fig. 8) Thoracomeres 5–8, sternum, shape: more or less straight anterior-posteriorly (0); narrowing anterior-posteriorly (1); widening anterior-posteriorly (2).
5. (Fig. 9) Thoracomeres 5–6 and 8, pleurum, medial margin, shape: relatively straight (0); with prominent expansions (1).
6. (Fig. 7E–L) Thoracomeres 5–8, pleurum, gill openings, number and positions: 4 in pleurites 5–8 (0); 2 only in pleurites 5–6 (1).

**Fig. 10:**
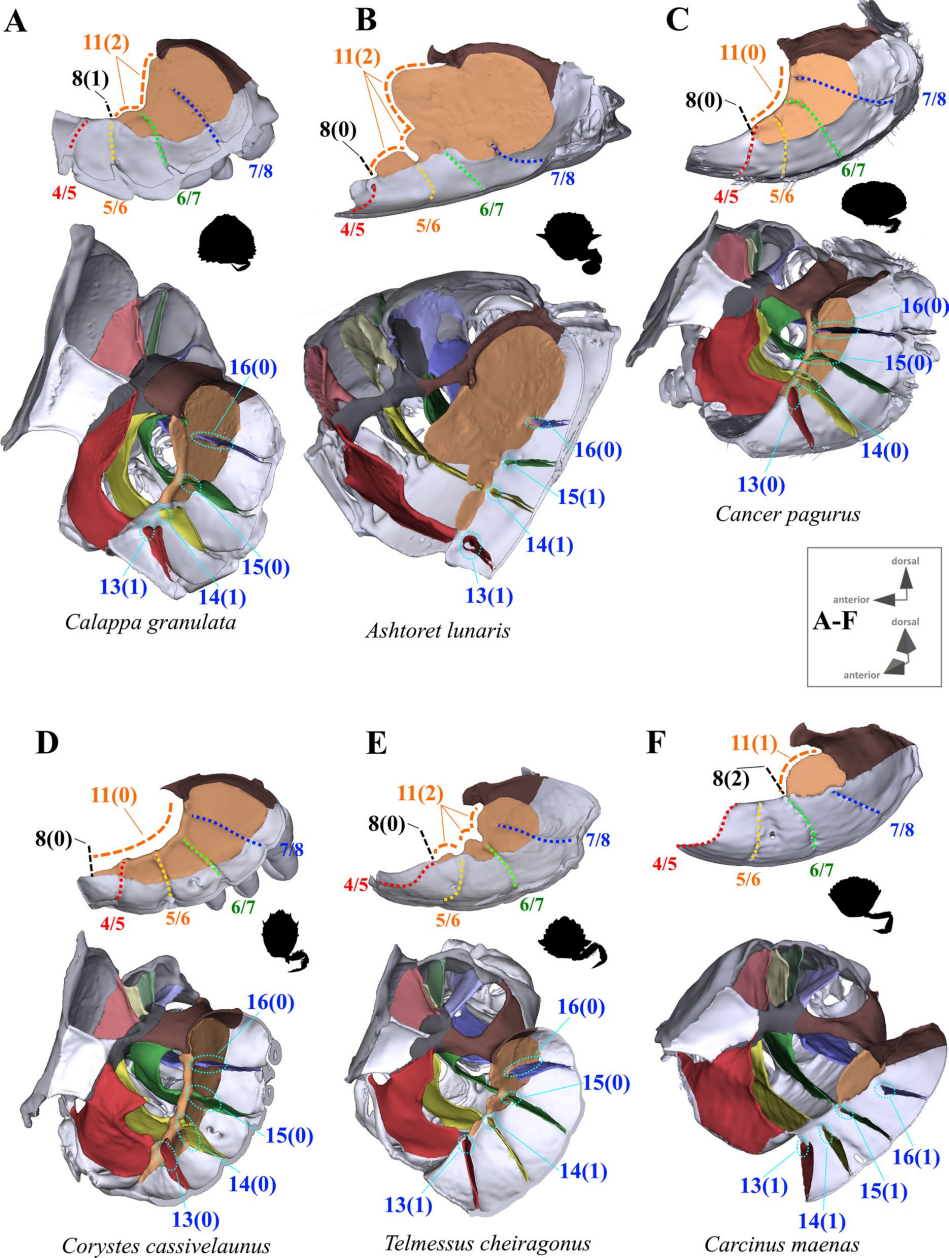
Examples showing characters concerned with anterior median plate extension (character 8), shape of dorsal median plate margin (character 11) and absence or presence of connections between interosternites and the median plate (characters 13–16) in axial skeletons seen from lateral (upper images) and from anterio-dorsal (lower images). In lateral view, number pair separated by backslash indicates thoracomeres between which the respective interosternite is situated. **A** Calappidae (Calappoidea), **B** Matutidae (Calappoidea), **C** Cancridae (Cancroidea), **D** Corystidae (Corystoidea), **E** Cheiragonidae (Corystoidea), **F** Carcinidae (Portunoidea)

**Fig. 11:**
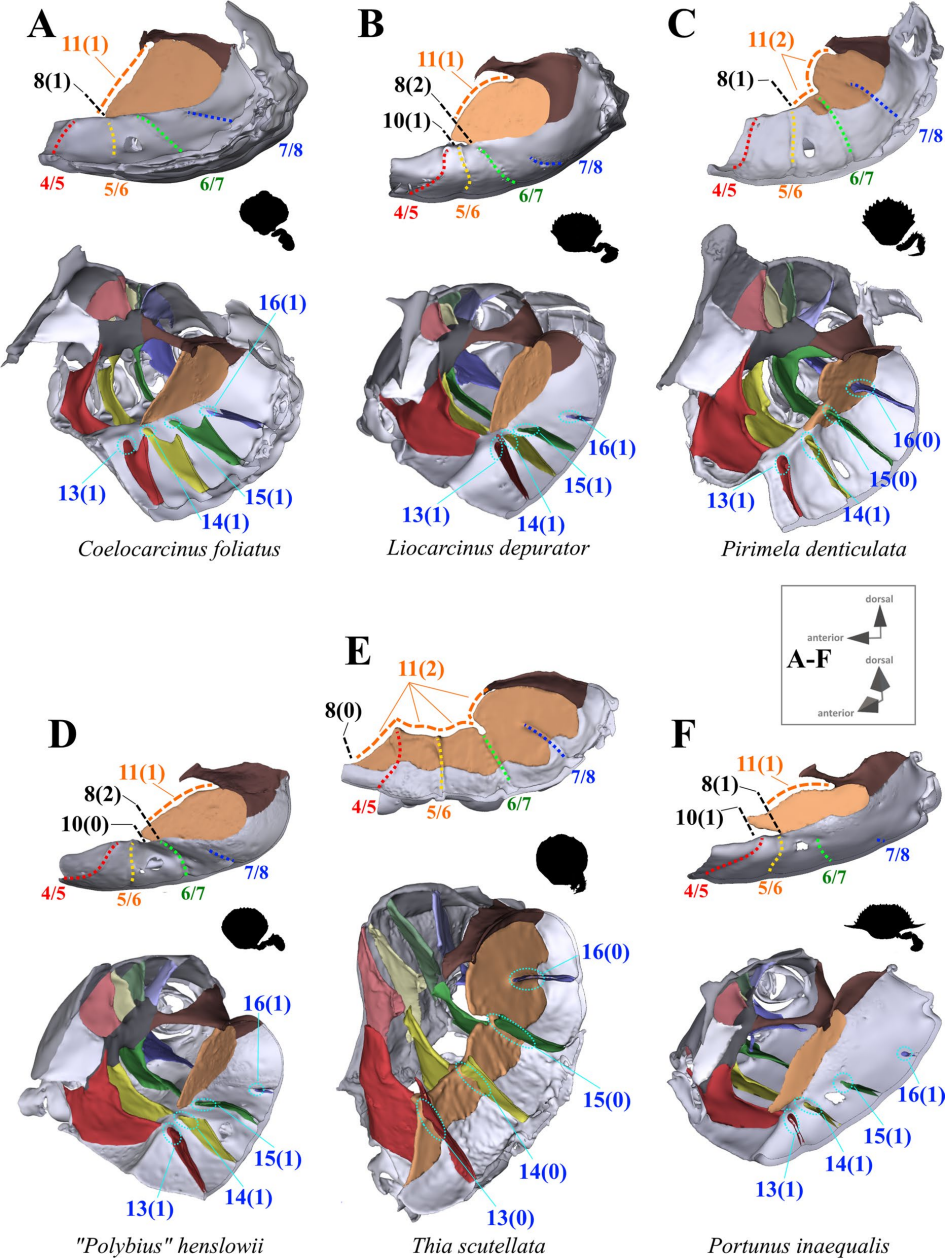
Examples showing characters concerned with anterior median plate extension (character 8), length of median plate anterior process (if present; character 10), shape of dorsal median plate margin (character 11) and absence or presence of connections between interosternites and the median plate (characters 13–16) in axial skeletons seen from lateral (upper images) and from anterio-dorsal (lower images). Number pair separated by backslash in lateral view indicates thoracomeres between which the respective interosternite is situated. **A**–**E** Carcinidae (Portunoidea), **F** Portunidae (Portunoidea)

**Fig. 12:**
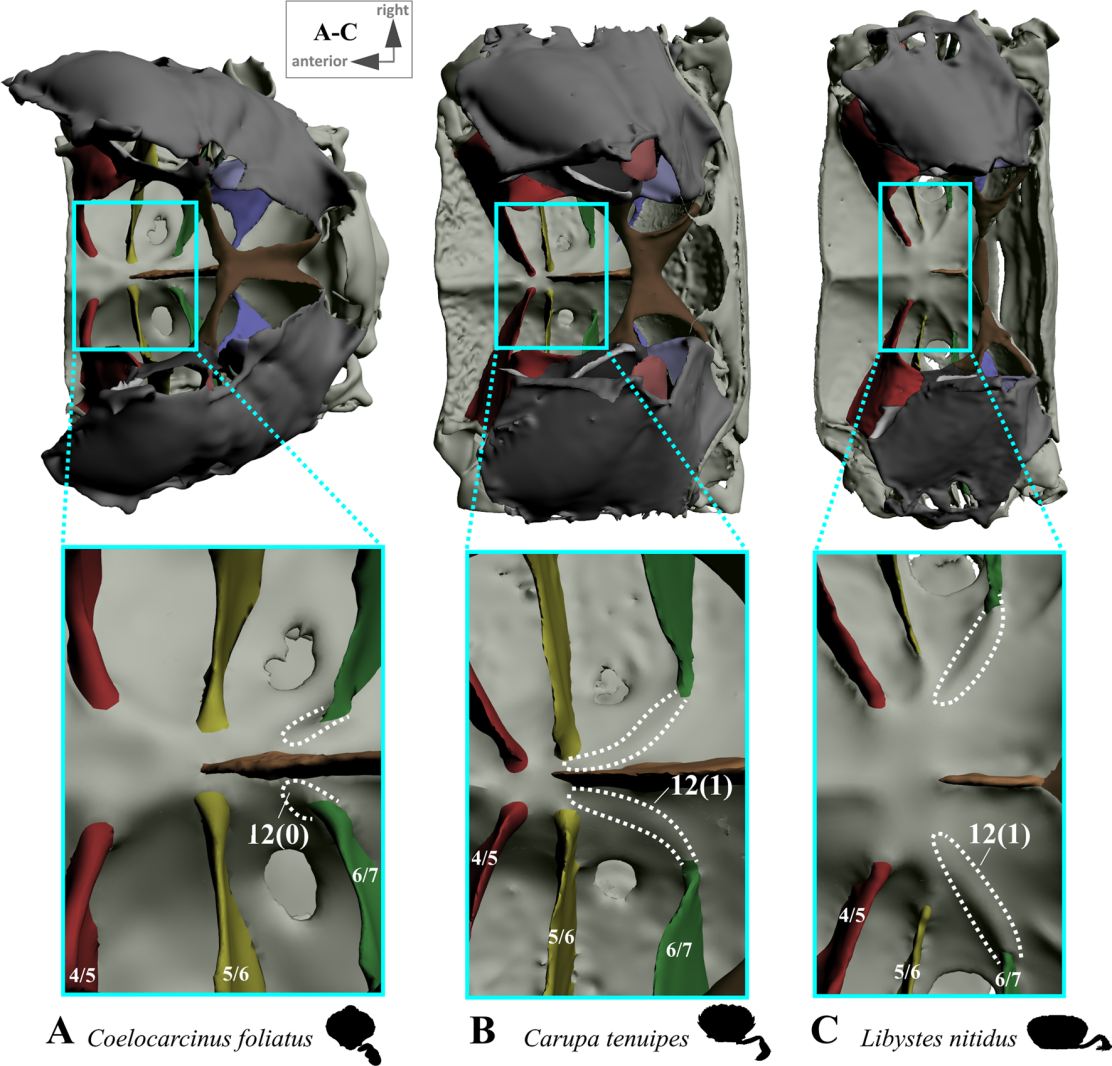
Examples showing character 12, which is concerned with the absence or presence of a transverse sternal ridge from interosternite 6/7 to the anterior end of the median plate in axial skeletons seen from dorsal. Number pair separated by backslash indicates thoracomeres between which the respective interosternite is situated. **A** Carcinidae (Portunoidea), **B**, **C** Portunidae (Portunoidea)

*Extension and shape of median plate* In the outgroup taxon *Sternodromia monodi,* a median plate is absent (Fig. 1A). This is also the case in *Medorippe lanata* (Fig. 3), which differs from all other taxa in the males exhibiting sternal infoldings which rise up medially in thoracomeres 5, 6 and 7 to 8 (not connected to the sella turcica). These infoldings are absent in female specimens due to the difference in sternum breadth between the sexes. Males have a narrower sternum than females, with the P2 and P3 ventral basi-ischium muscles originating at the medial sternal infoldings, while in females, the broad sternum offers an area large enough on its own to serve as an attachment site for these muscles. In all other taxa, the median plate is present in thoracomere 8 of both female and male specimens, and posteriorly connected to the sella turcica (the degree of anterior extension varies; see for example Figs. 1, 2, 3, 4, 5, 6). Therefore, we consider the median sternal infolding in *M. lanata* males (termed “median plate” by [19]) not to be homologous to the median plate in the remaining taxa (i.e. the median plate in *M. lanata* is scored as absent; character state 7(0)). Character 8 refers to the maximal extension of the median plate in an anterior direction, not including the extension (“anterior process”) formed by the anterior margin of the median plate (character 9, 10; Fig. 11B, D, F). The degree of anterior extension of the median plate can affect the shape of its dorsal margin. In taxa that exhibit a median plate reaching interosternite 4/5 (character state 8(0); Figs. 10B–E, 11E) the dorsal margin may be concave and without indentations and/or gaps (character state 11(0); Figs. 10C, D) or irregular in shape, with indentations and/or gaps between thoracomeres (character state 11(2); Figs. 10E, 11E). Taxa with a median plate reaching interosternite 5/6 (character state 8(1); Figs. 10A, 11A, C, F) can exhibit a more or less convex margin with no indentations and/or gaps (character state 11(1); Fig. 11A, F), or one that is irregularly shaped (with indentations and/or gaps; character state 11(2); Figs. 10A, 11C). Only in taxa with a median plate extending to interosternite 6/7 is the median plate margin always convex without indentations and/or gaps between thoracomeres (character states 8(2), 11(1); Figs. 10F, 11B, D). In several taxa in which the median plate reaches interosternite 5/6 (character state 8(1)), has a convex dorsal margin (character state 11(1)), and no connection to interosternite 6/7 (character state 15(1); see below), a transverse sternal ridge runs from the lower medial edge of interosternite 6/7 towards the anterior end of the median plate (not including the anterior process that is present in some species; character state 12(1); Fig. 12).

7. Sternum, median plate: absent (0); present (1).
8. (Figs. 10, 11) Sternum, median plate, maximal anterior extension: up to or further than interosternite 4/5 (0); up to interosternite 5/6 (1); up to interosternite 6/7 (2); inapplicable (–) if 7(0).
9. (Figs. 10, 11) Sternum, median plate, anterior process: absent (0); present (1), inapplicable (–) if 7(0) or 8(0).
10. (Fig. 11B, D, F) Sternum, median plate, anterior process, length: overlapping up to one thoracomere (0) overlapping more than one thoracomere (1); inapplicable (–) if 7(0) or 9(0).
11. (Figs. 10, 11) Sternum, median plate, dorsal margin, shape: concave, without indentations and/or gaps between thoracomeres (0); more or less convex, without indentations and/or gaps between thoracomeres (1); irregular, with indentations and/or gaps between thoracomeres (2); inapplicable (–) if 7(0).
12. (Fig. 12) Sternum, transverse sternal ridge from interosternite 6/7 to anterior end of median plate: absent (0); present (1); inapplicable (–) if 7(0), 8(0), 8(2), 8(3) or 15(0).

**Fig. 13:**
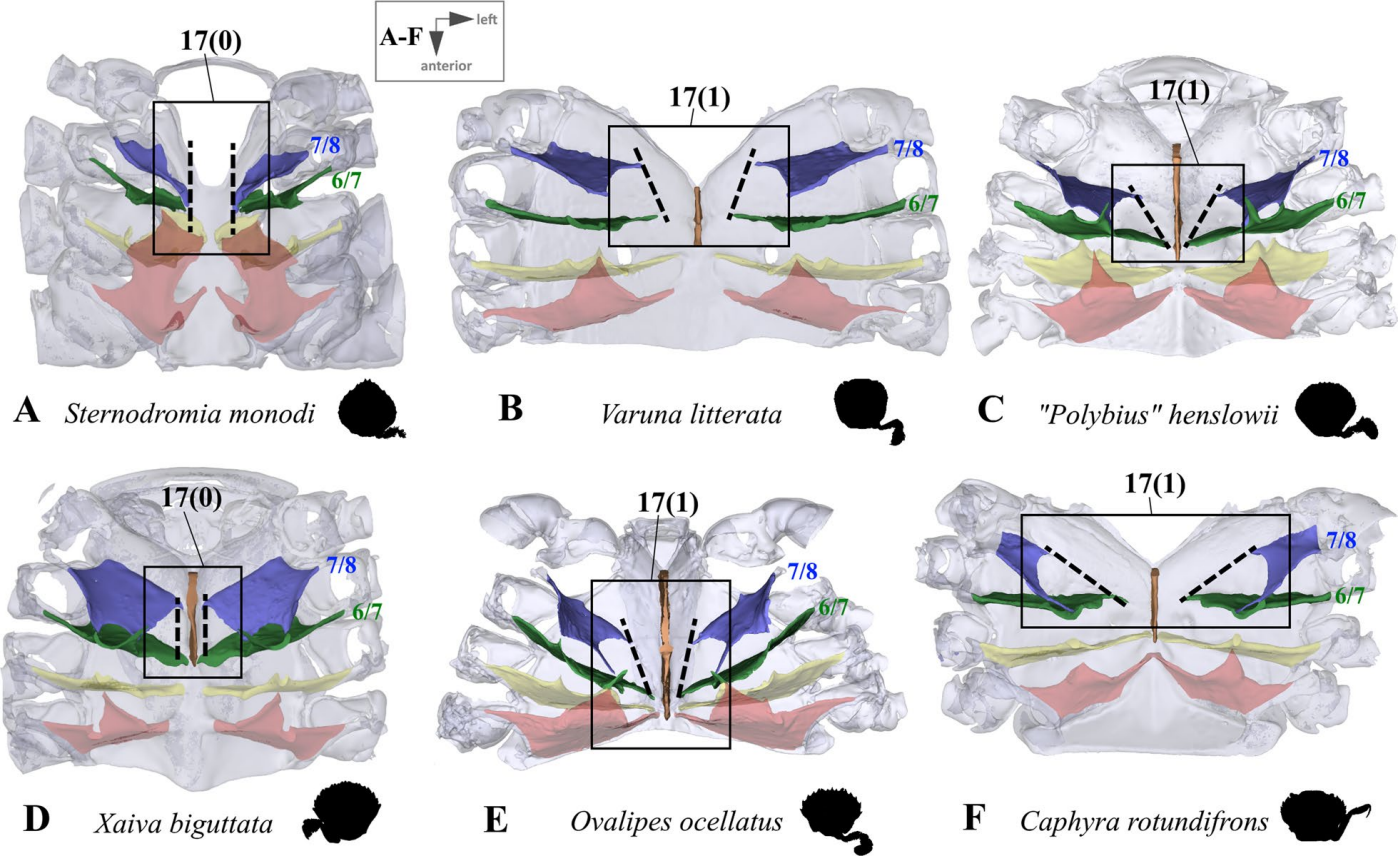
Examples showing character 17, which is concerned with the distance from medial edge to median plane in interosternite 6/7 compared to interosternite 7/8 in axial skeletons seen from dorsal. Number pair separated by backslash indicates thoracomeres between which the respective interosternite is situated. **A** Dromiidae (Podotremata), **B** Varunidae (Thoracotremata), **C**, **D** Carcinidae (Portunoidea), **E** Geryonidae (Portunoidea), **F** Portunidae (Portunoidea)

*Connection between interosternites and the median plate and distance to median plane* The interosternites which are connected to the median plate differ between some taxa (Figs. 1, 2, 3, 4, 5, 6, 10, 11; in several species, the interosternites lack any connection). In species in which the median plate only reaches interosternites 5/6 or 6/7 (character states 8(1), 8(2); Fig. 10A, F, 11A–D, F), interosternite 4/5 can never be connected to the median plate (and character 13 is thus scored as inapplicable). The same is true of interosternite 5/6 (character 14) in species in which the median plate only reaches interosternite 6/7 (character state 8(2); Figs. 10F, 11B, D). In several taxa the distance between the medial edge of interosternite 7/8 and the median plane is greater than that between interosternite 6/7 and the median plane (character state 17(1); Fig. 13). In taxa in which interosternite 7/8 is connected to the median plate (character state 16(0); Figs. 10A–E, 11C, E), the distance between the medial edge of interosternite 7/8 and the median plane is necessarily similar to that between interosternite 6/7 and the median plane (and character 17 is thus scored as inapplicable).

13. (Figs. 10, 11) Sternum, interosternite 4/5, connection to median plate: present (0); absent (1); inapplicable (–) if 7(0), 8(1) or 8(2).
14. (Figs. 10, 11) Sternum, interosternite 5/6, connection to median plate: present (0); absent (1); inapplicable (–) if 7(0) or 8(2).
15. (Figs. 10, 11) Sternum, interosternite 6/7, connection to median plate: present (0); absent (1); inapplicable (–) if 7(0).
16. (Figs. 10, 11) Sternum, interosternite 7/8, connection to median plate: present (0); absent (1); inapplicable (–) if 7(0).
17. (Fig. 13) Sternum, interosternite 7/8, distance between medial edge and median plane: similar as in interosternite 6/7 (0); greater than in interosternite 6/7 (1); inapplicable (–) if 7(0) or 16(0).

**Fig. 14:**
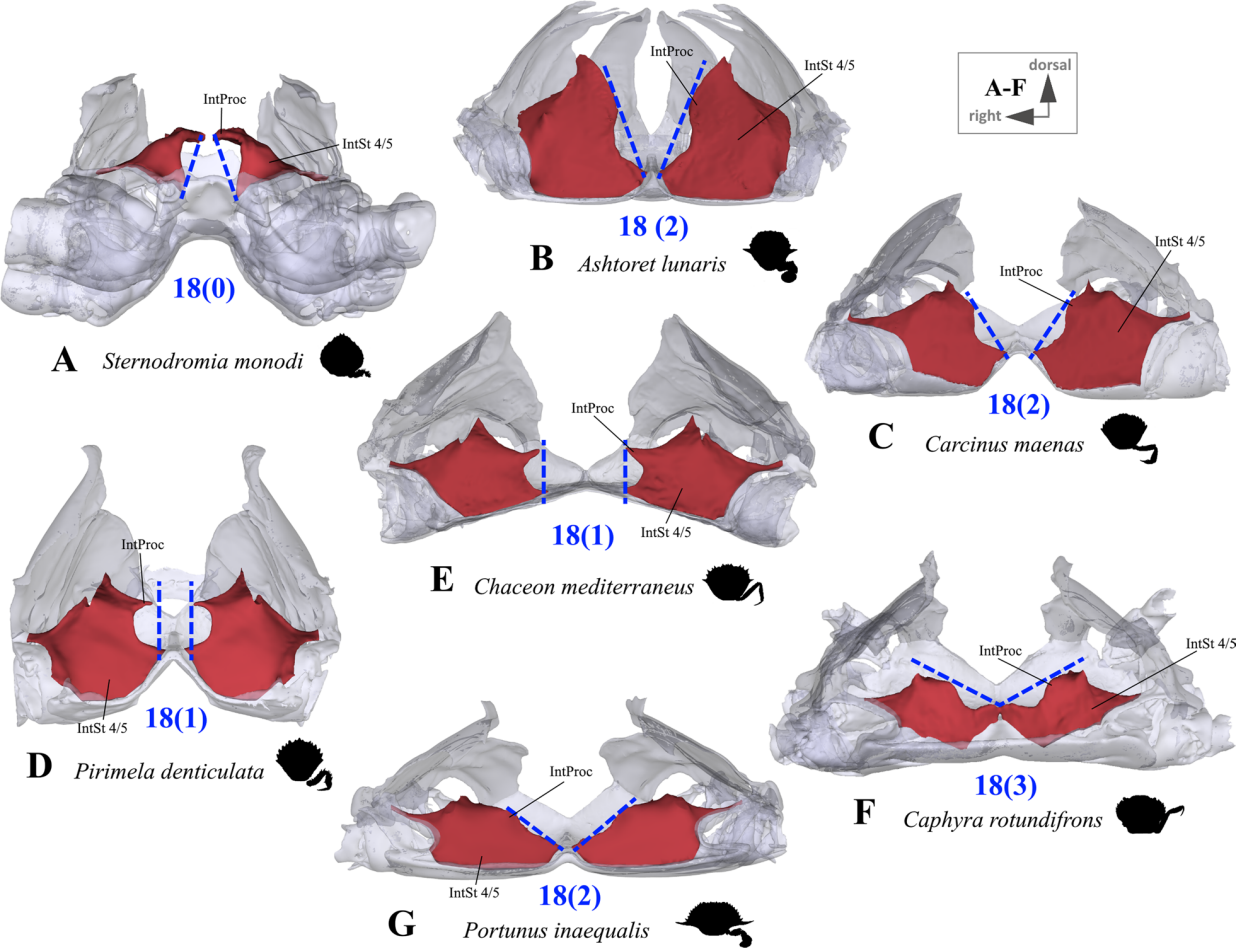
Examples showing character 18, which is concerned with the shape of the medial margin of interosternite 4/5 seen from anterior. **A** Dromiidae (Podotremata), **B** Matutidae (Calappoidea), **C**, **D** Carcinidae (Portunoidea), **E** Geryonidae (Portunoidea), **F**, **G** Portunidae (Portunoidea). *IntProc* interosternal process, *IntSt 4/5* interosternite between thoracomeres 4 and 5

*Medial margins of interosternites 4/5 to 6/7* The medial margin of an interosternite is characterised by an upper, medially directed interosternal expansion we herein term “interosternal process” (Fig. 14; for more information on the terminology of morphemes, see [22]). As it is difficult to conceptualize the morpheme variability seen in the different taxa into distinct character states, we only distinguish here between three discrete states which are not prone to subjective perception (character 18, Fig. 14). In all cases, the distinctiveness of processes decreases progressively from interosternite 4/5 to interosternite 6/7. For this reason, this character is conceptualized by the process shape of interosternite 4/5 only.

18. (Fig. 14) Sternum, interosternite 4/5, medial margin, shape: transversal with interosternal process being most medial (0); almost perpendicular (1); transversal with lower margin being most medial but not touching interosternite 4/5 of other lateral side (2); transversal with lower margin being most medial and touching interosternite 4/5 of other lateral side (3).

**Fig. 15:**
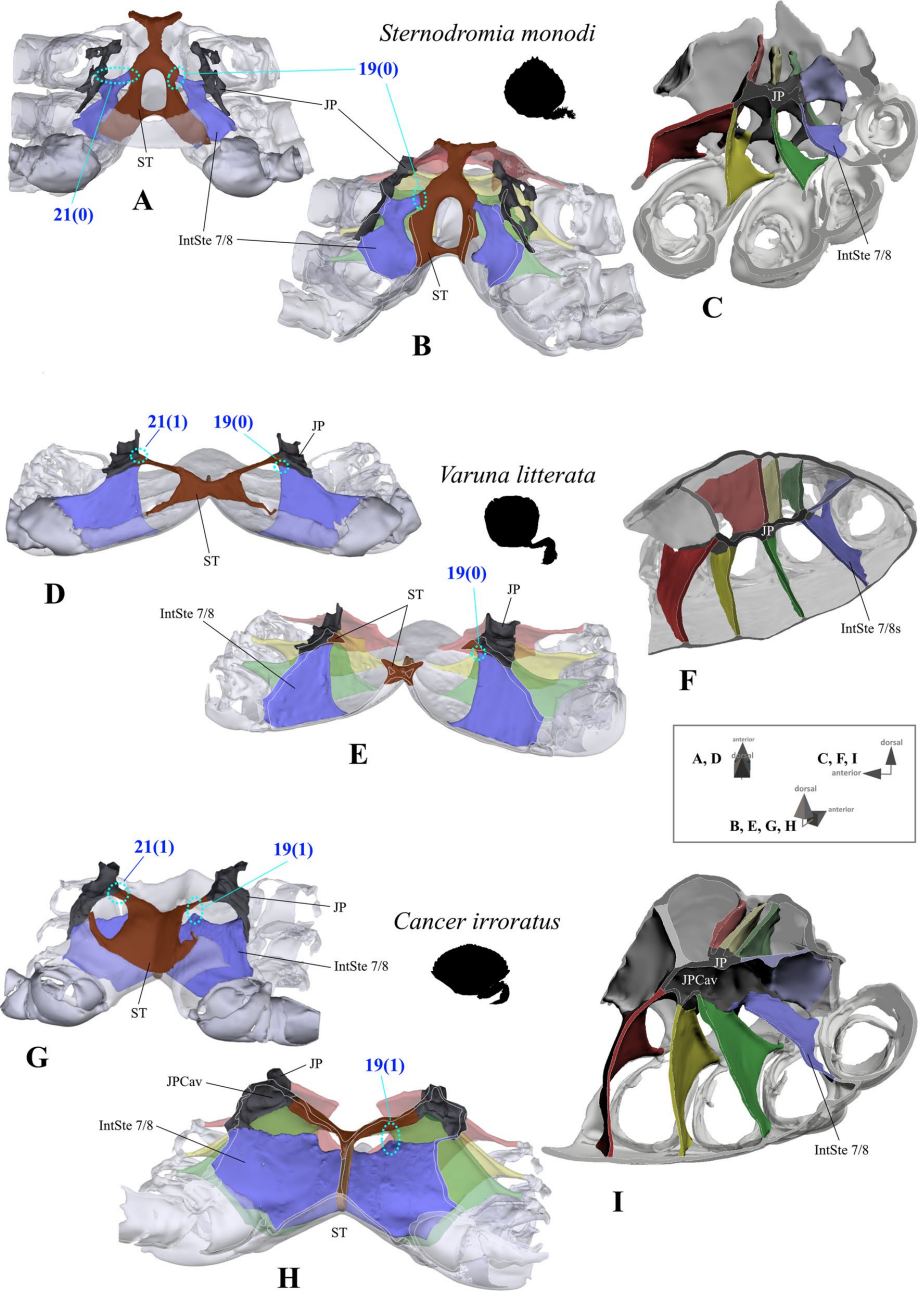
Examples showing character states concerned with the absence or presence of a connection between interosternite 7/8 and the sella turcica (character 19) and the absence or presence of a connection between the junction plate and the sella turcica (character 21) in the axial skeleton seen from posterior (**A**, **B**, **D**, **E**, **G**, **H**). Note that in the species in which interosternite 7/8 is connected to the sella turcica (character state 19(0); **B**, **E**), the junction plate does not form a cavity, while in the species without a connection (character state 19(1); **G**, **H**), a junction plate cavity is present (**I**). *IntSt 7/8* interosternite between thoracomeres 7 and 8, *JP* junction plate, *JPCav* junction plate cavity, *ST* sella turcica

**Fig. 16:**
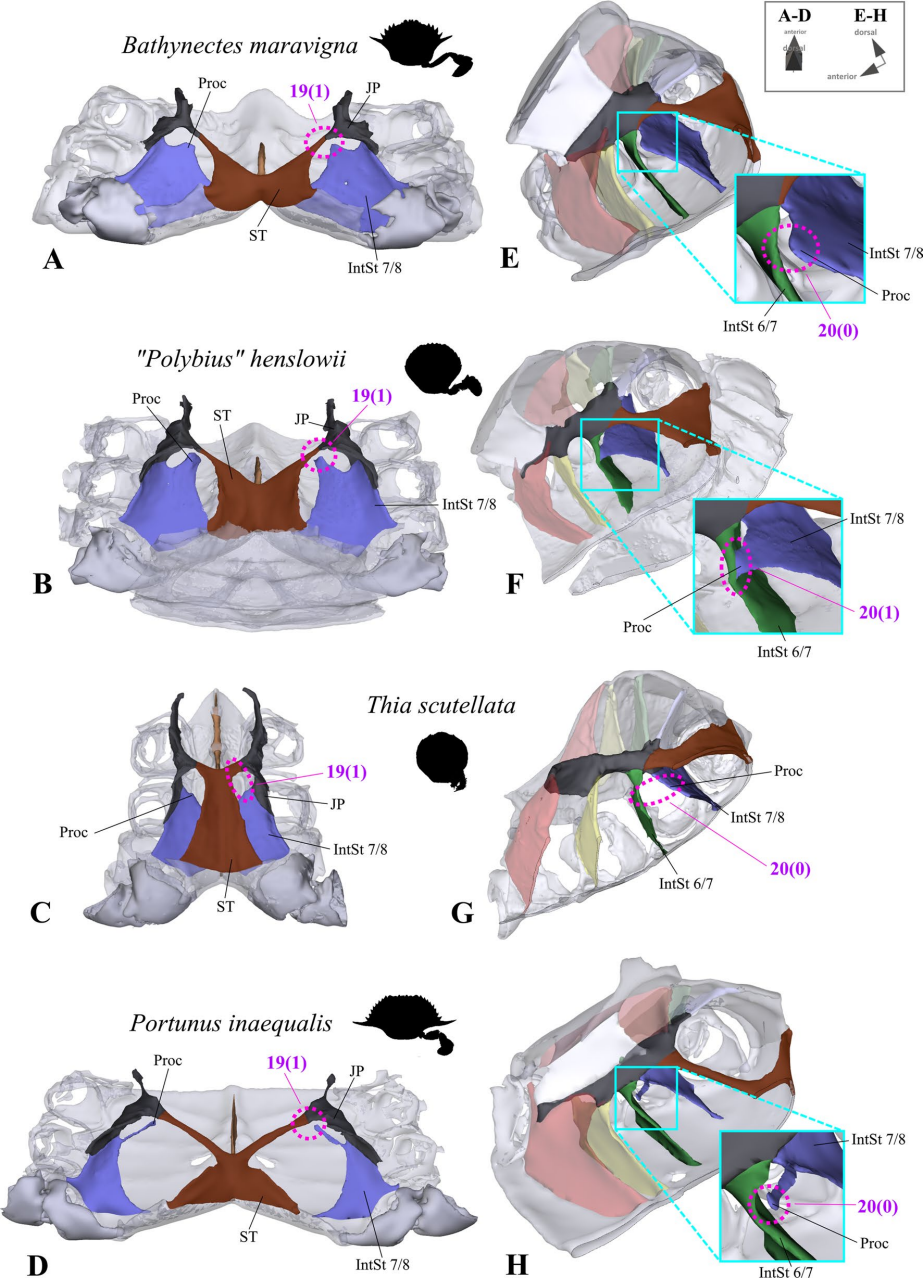
Variabilty in the shape of the process of interosternite 7/8. **A**–**D** Examples showing character states concerned with the absence or presence of a connection between interosternite 7/8 and the sella turcica (character 19) in the axial skeleton seen from postero-dorsal, **E**–**H** examples showing character states concerned with the absence or presence of a connection between interosternite 7/8 process and interosternite 6/7 (character 20) in the axial skeleton seen from the left side. *IntSt 6/7* interosternite between thoracomeres 6 and 7, *IntSt 7/8* interosternite between thoracomeres 7 and 8, *JP* junction plate, *Proc* interosternal process of interosternite 7/8, *ST* sella turcica

*Connection between interosternite 7/8 and sella turcica and interosternal process of interosternite 7/8* In non-heterotrematan outgroup taxa and *Medorippe lanata*, interosternite 7/8 is directly connected to the sella turcica (character state 19(0); Fig. 15A, B, D, E). That which we term here “interosternal process 7/8” is a medio-anteriorly directed process at the upper medial margin of interosternite 7/8, only present in all remaining taxa, in which interosternite 7/8 is not connected to the sella turcica (character state 19(1); Fig. 15G, H). Its shape can be quite variable (and is thus vulnerable to subjectivity): It may, for example, be barely visible as in *Thia scutellata* (J. C. Fabricius, 1793) (Fig. 16C, G), it may be distinct and short as in *Bathynectes maravigna* (Prestandrea, 1839) and *“Polybius” henslowii* (Fig. 16A, B, E, F), or very long as in *Portunus inaequalis* (Fig. 16D, H). However, in several species, the process touches interosternite 6/7, thus constituting an intersubjective morphological feature that can be used to conceptualize a character (character 20; Fig. 16E–H).

19. (Fig. 15A, B, D, E, G, H, 16A–D) Sternum, interosternite 7/8, connection to sella turcica: present (0); absent (1).
20. (Fig. 16E–H) Sternum, interosternite 7/8, interosternal process, contact to interosternite 6/7: absent (0); present (1); inapplicable (–) if 19(0).

**Fig. 17:**
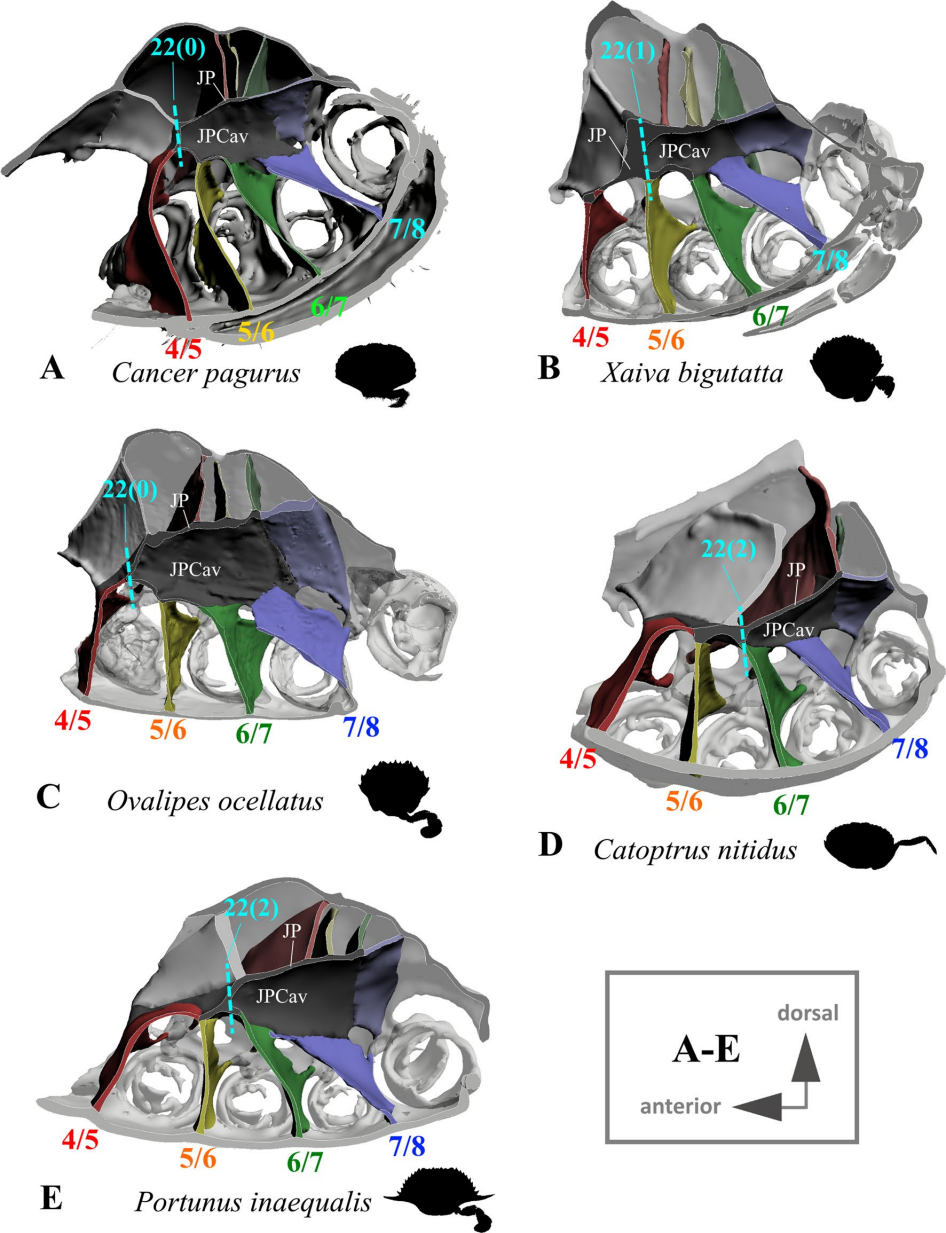
Variability in the shape of the junction plate cavity with examples showing character states concerned with the anterior extension of the junction plate (character 22). Number pair separated by backslash indicates thoracomeres between which the respective interosternite is situated. **A** Cancridae (Cancroidea), **B** Carcinidae (Portunoidea), **C** Geryonidae (Portunoidea), **D**, **E** Portunidae (Portunoidea). *JP* junction plate, *JPCav* junction plate cavity

**Fig. 18:**
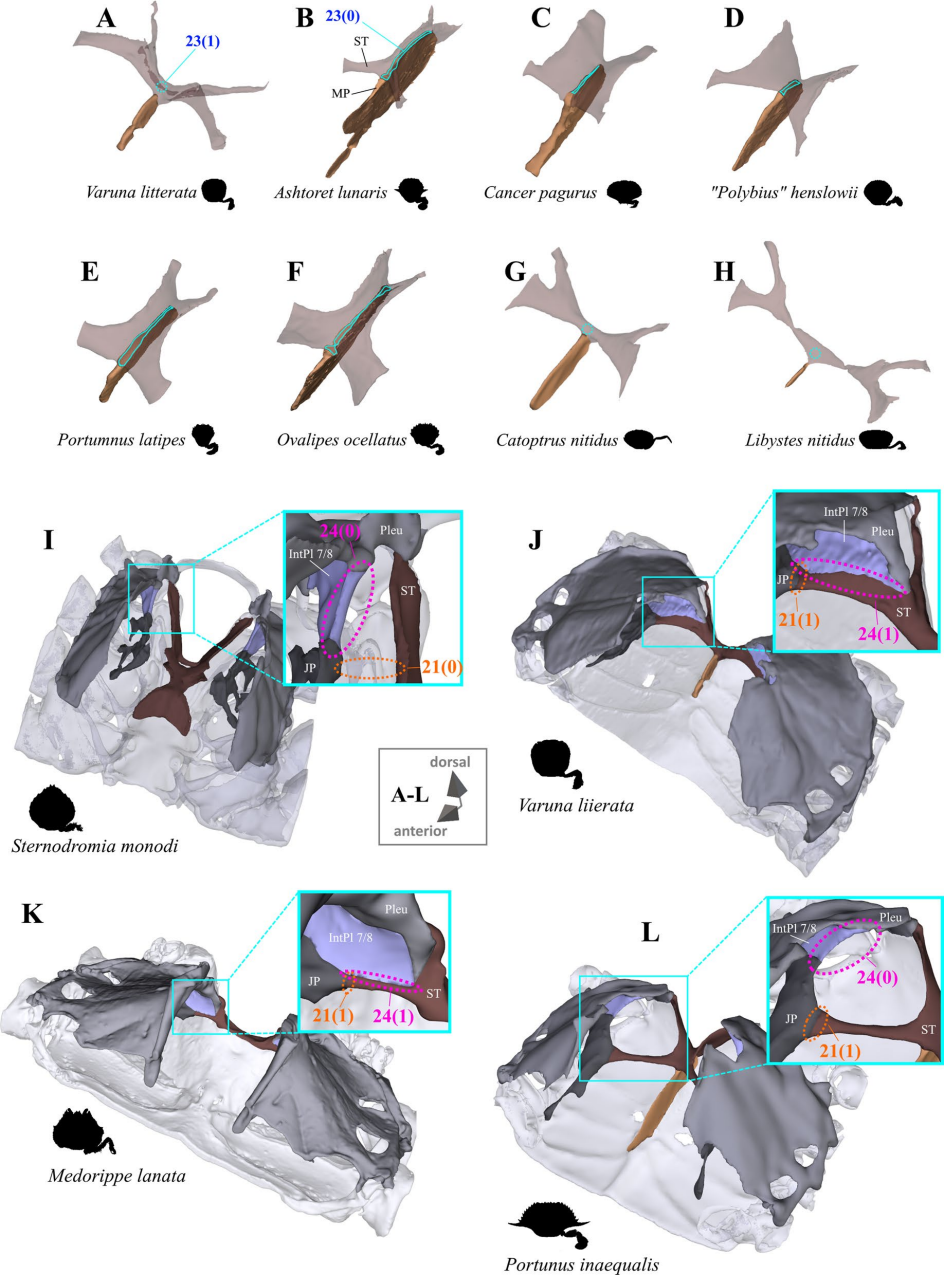
Variability in the shape of the sella turcica and in its connection with interopleurite 7/8 and the junction plate. **A**–**H** Examples showing character states concerned with the degree of coverage of the dorsal median plate margin by the sella tucica (character 23), **I**–**L** examples showing character states concerned with the presence or absence of a connection between the junction plate and the sella turcica (character 21) and character states concerning the degree of conncection between interopleutite 7/8 and the sella turcica (character 24). *IntPl 7/8* Interopleurite between thoracomeres 7 and 8, *JP* junction plate, *MP* median plate, *Pleu* pleurum, *ST* sella turcica

*Shape of junction plate and sella turcica* In all ingroup taxa (in which interosternite 7/8 is not connected to the sella turcica; character state 19(1); Figs. 15G, H, 16A–D), each junction plate forms a cavity whose extension in an anterior direction is variable (Figs. 15H, I, 17). This is absent in all non-heterotrematan outgroup taxa (in which interosternite 7/8 is connected to the sella turcica; character 19(0); Fig. 15A–F). The shape of the “junction plate cavity” (for more information on the terminology of morphemes see [22]) is very variable between taxa, in most cases forming an anteriorly closed calyx with an asymmetrical posterior margin of which the ventral part is more anteriorly situated than the dorsal part (Fig. 15H, I, 17A–C, E). In some taxa, the ventral part of the calyx is completely missing, with the junction plate cavity rather resembling a convex roof (Fig. 17D). We here conceptualize the extension of the junction plate cavity in an anterior direction as a character, with states corresponding to the interosternites which are reached by the anterior end of this cavity (character 22; Fig. 17). In contrast to Guinot et al. [19], who suggested that in Podotremata a junction plate is lacking, we found in *Sternodromia monodi* that interosternites and interopleurites are indeed fused, forming a structure we here consider as a junction plate. However, the fusion is not as complete as in the other taxa, so that the junction plate appears to have large gaps (Fig. 1A). The sella turcica in *S. monodi* differs from that in all other taxa not only by not being directly connected with the two junction plates (character 21; Figs. 15A, D, G, 16A–D, 18I–L), but also by being fused with interosternites 4/5 to 7/8 (Fig. 1A). As borders between the elements of this fusion cannot be distinguished unambiguously, they are considered as diffuse transitions in 3D reconstructions, with the fusion reconstructed as being part of the sella turcica. The shape of the sella turcica is variable between taxa, making character conceptualization difficult (Fig. 18A–H). We here conceptualize its shape in only one unambiguous character which expresses whether the sella turcica covers the dorsal median plate margin (character 23; Fig. 18A–H). In outgroup taxa *Eriocheir sinensis, Varuna litterata*, and *Medorippe lanata*, the whole medial margin of interopleurite 7/8 is confluent with the sella turcica, while in all other taxa, the medial margin of interopleurite 7/8 is not or is only partly connected to the sella turcica (character 24; Fig. 18I–L).

21. (Figs. 15A, B, D, E, G, H, 18I–L) Junction plate, connection to sella turcica: absent (0); present (1).
22. (Fig. 17) Junction plate cavity, maximal anterior extension: reaching interosternite 5/6 (0); reaching interosternite 6/7(1); reaching interosternite 7/8 (2); inapplicable (–) if 19(0) or 21(0).
23. (Fig. 18A–H) Sella turcica covering dorsal median plate margin: present (0); absent (1); inapplicable (–) if 7(0).
24. (Fig. 18I–L) Pleurum, interopleurite 7/8, medial margin, degree of connection to sella turcica: not or only partly connected (0); completely connected (1).

**Fig. 19:**
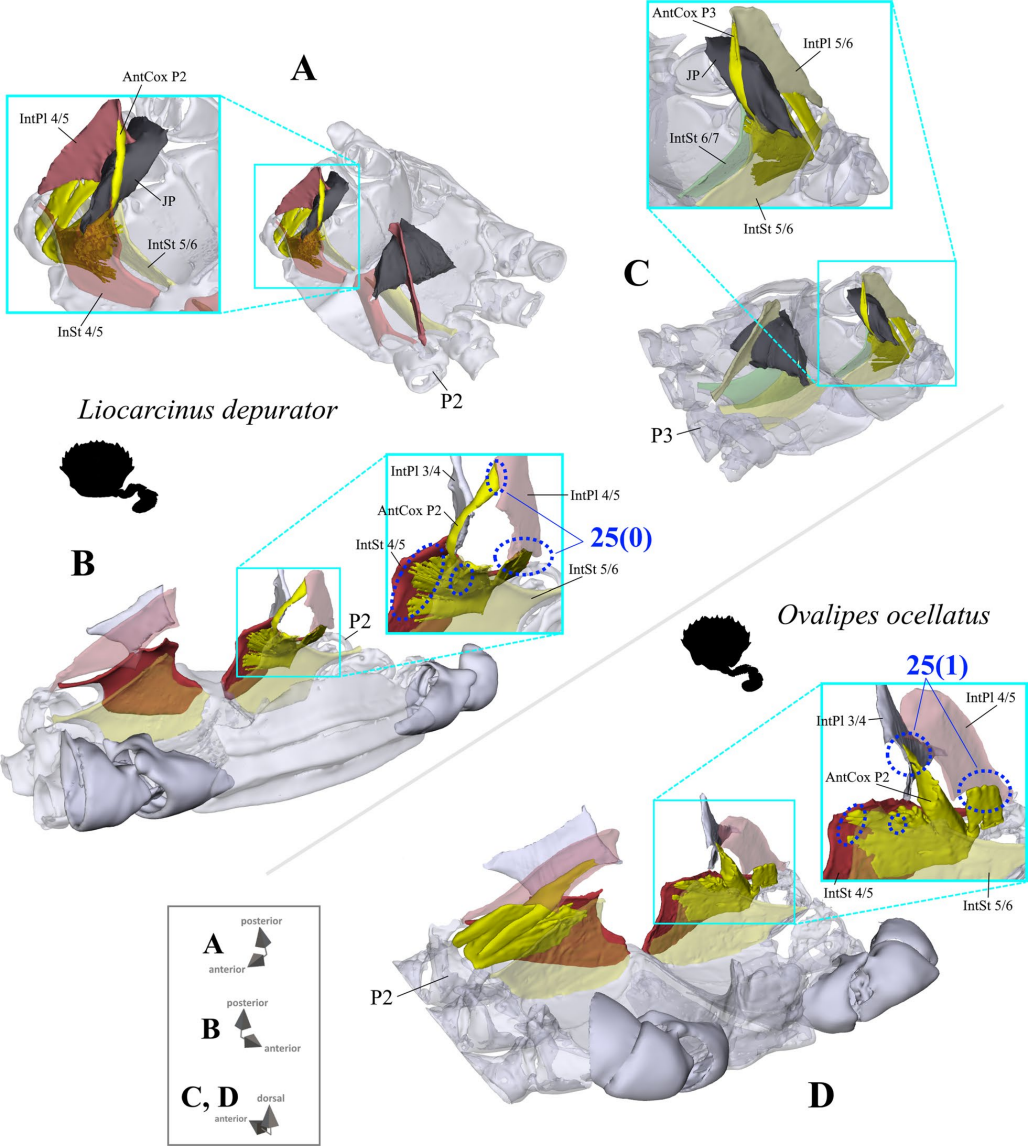
Examples showing character states concerned with the origin of the anterior coxa muscle of the 2nd pereiopod (character 25; **A**, **B**, **D**) and the shape of the anterior coxa muscle of the 3rd pereiopod (**C**) in axial skeletons seen from different perspectives. Note that in *Liocarcinus depurator*, for example, both muscles have a branch running along but not attaching at the medial side of the junction plate and originating at interopleurite 4/5 and 5/6, respectively (**A**, **C**). *AntCox P2* 2nd pereiopod anterior coxa muscle, *AntCox P3* 3rd pereiopod anterior coxa muscle, *IntPl* interopleurite (with number pair indicating thoracomeres between which it is situated), *IntSt* interosternite (with number pair indicating thoracomeres between which it is situated), *JP* junction plate, *MP* median plate, *P2* proximal 2nd pereiopod podomeres, *P3* proximal 3rd pereiopod podomeres, *ST* sella turcica

**Fig. 20:**
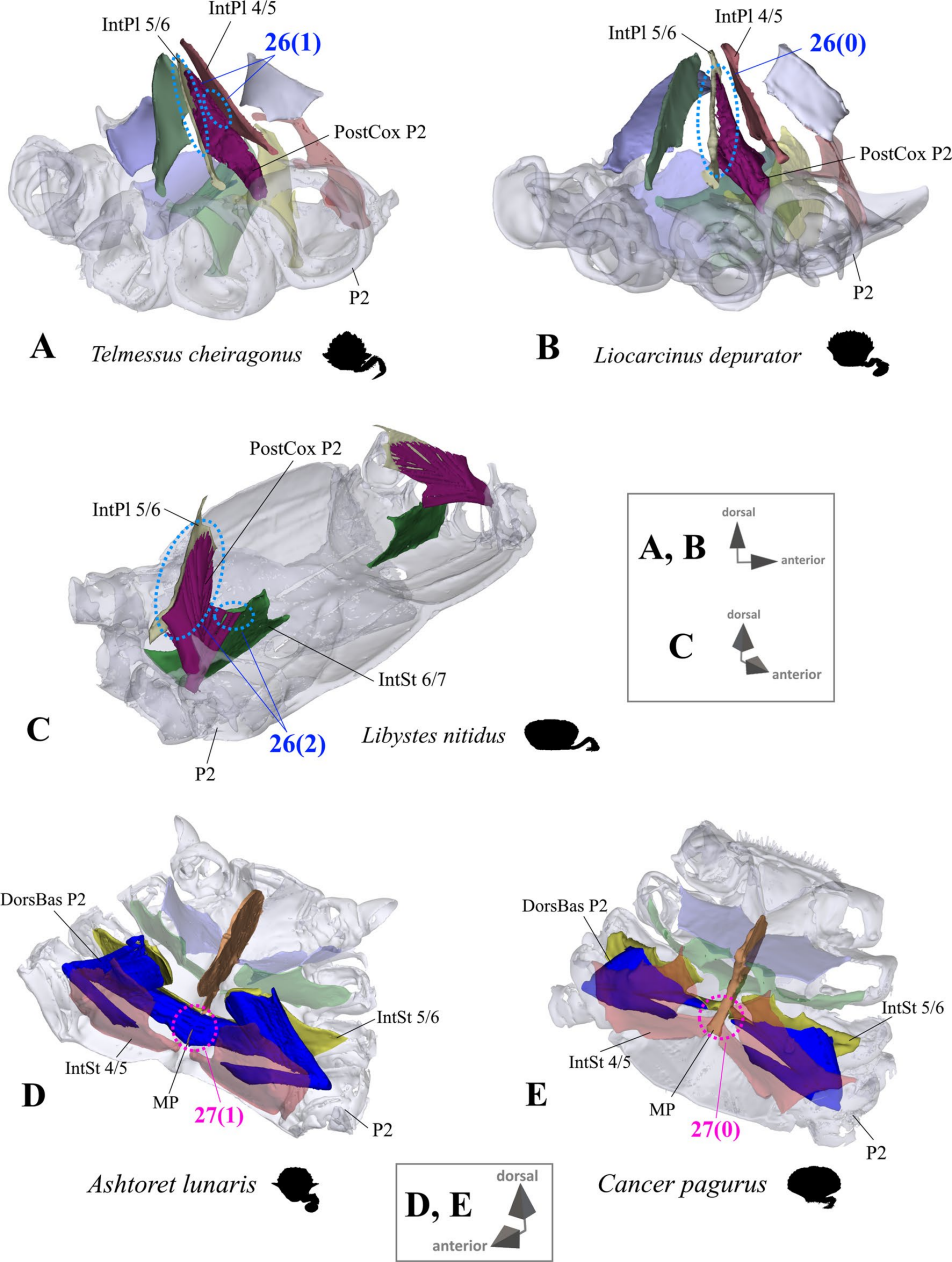
Examples showing character states concerned with the origin of the posterior coxa muscle of the 2nd pereiopod (character 26; **A**–**C**) and of the dorsal basi-ischium muscle of the 2nd pereiopod (character 27; **D**, **E**). *DorsBas P2* 2nd pereiopod dorsal basi-ischium muscle, *IntPl* interopleurite (with number pair indicating thoracomeres between which it is situated), *IntSt* interosternite (with number pair indicating thoracomeres between which it is situated), *MP* median plate, *P2* proximal 2nd pereiopod podomeres, *PostCox P2* 2nd pereiopod posterior coxa muscle, *ST* sella turcica

**Fig. 21:**
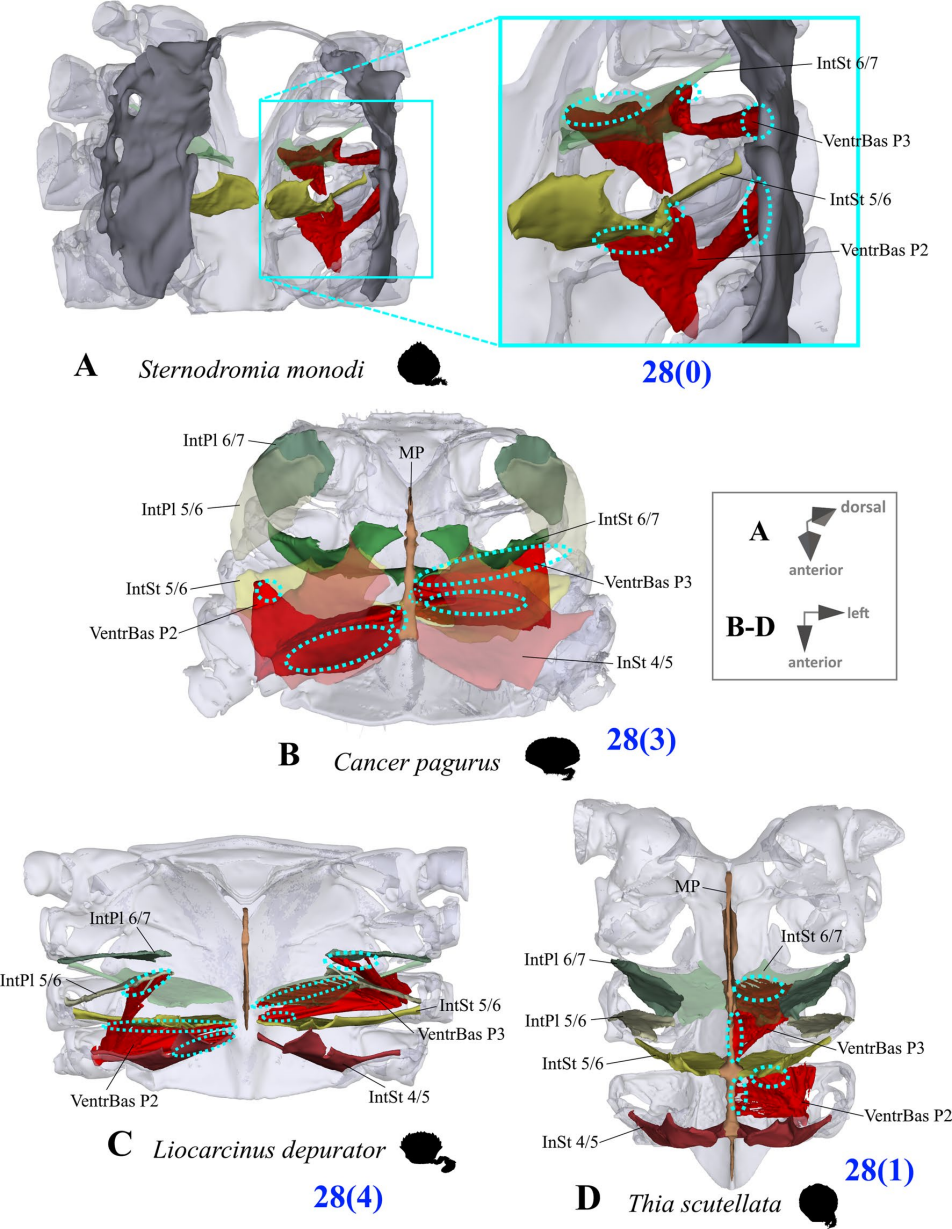
Examples showing character states concerned with the origin of the ventral basi-ischium muscles of the 2nd pereiopod and 3rd pereiopod (character 28) in axial skeletons seen from different perspectives. **A** Dromiidae (Podotremata), **B** Cancridae (Cancroidea), **C**, **D** Carcinidae (Portunoidea). *IntPl* interopleurite (with number pair indicating thoracomeres between which it is situated), *IntSt* interosternite (with number pairs indicating thoracomeres between which it is situated), *MP* median plate, *VentrBas P2* 2nd pereiopod ventral basi-ischium muscle, *VentrBas P3* 3rd pereiopod ventral basi-ischium muscle

**Fig. 22:**
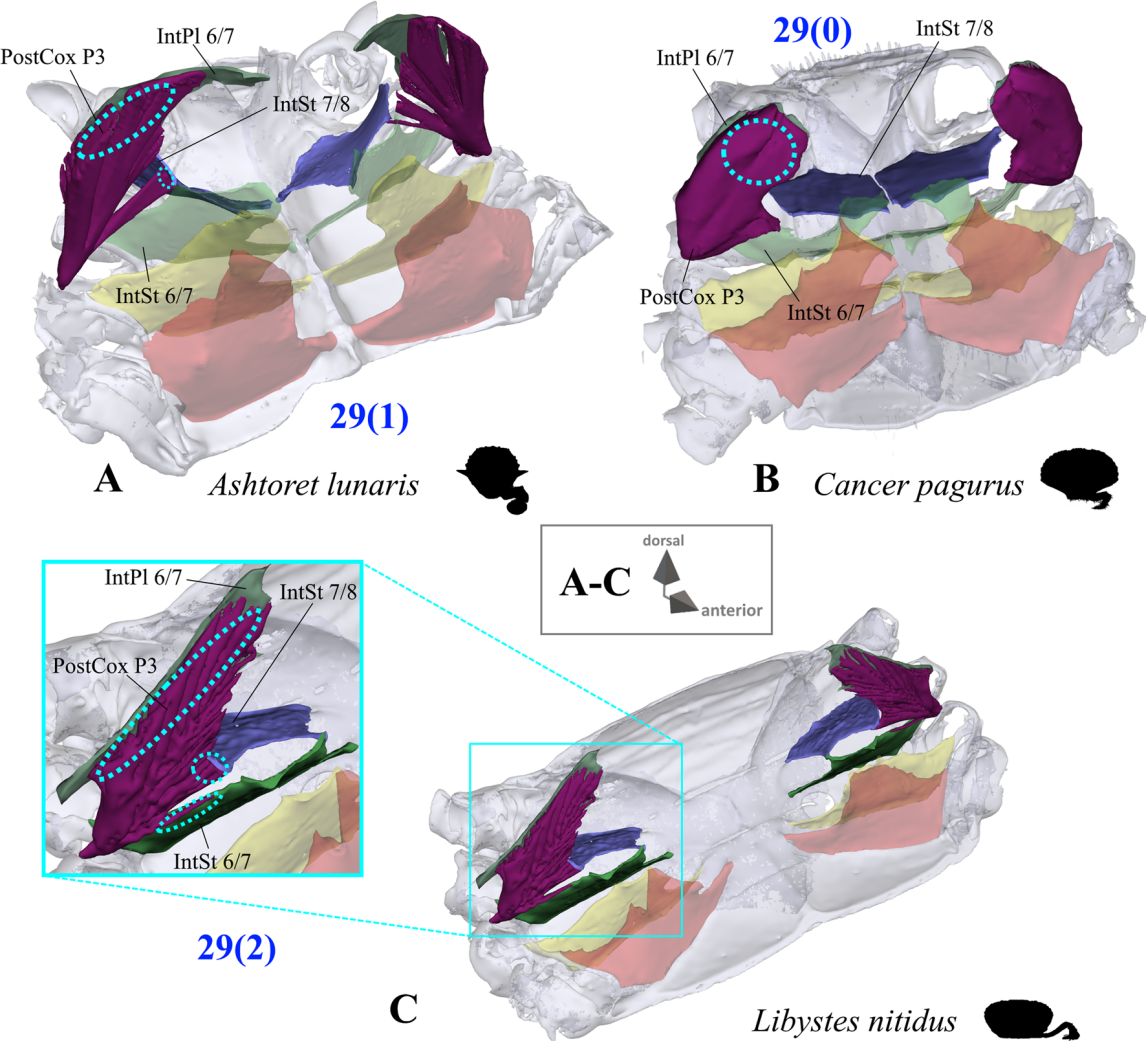
Examples showing character states concerned with the origin of the posterior coxa muscle of the 3rd pereiopod (character 29) in axial skeletons seen from anterio-dorsal. **A** Matutidae (Calappoidea), **B** Cancridae (Cancroidea), **C** Portunidae (Portunoidea). *IntPl 6/7* interopleurite between thoracomeres 6 and 7, *IntSt* interosternite (with number pair indicating thoracomeres between which it is situated), *PostCox P3* 3rd pereiopod posterior coxa muscle

**Fig. 23:**
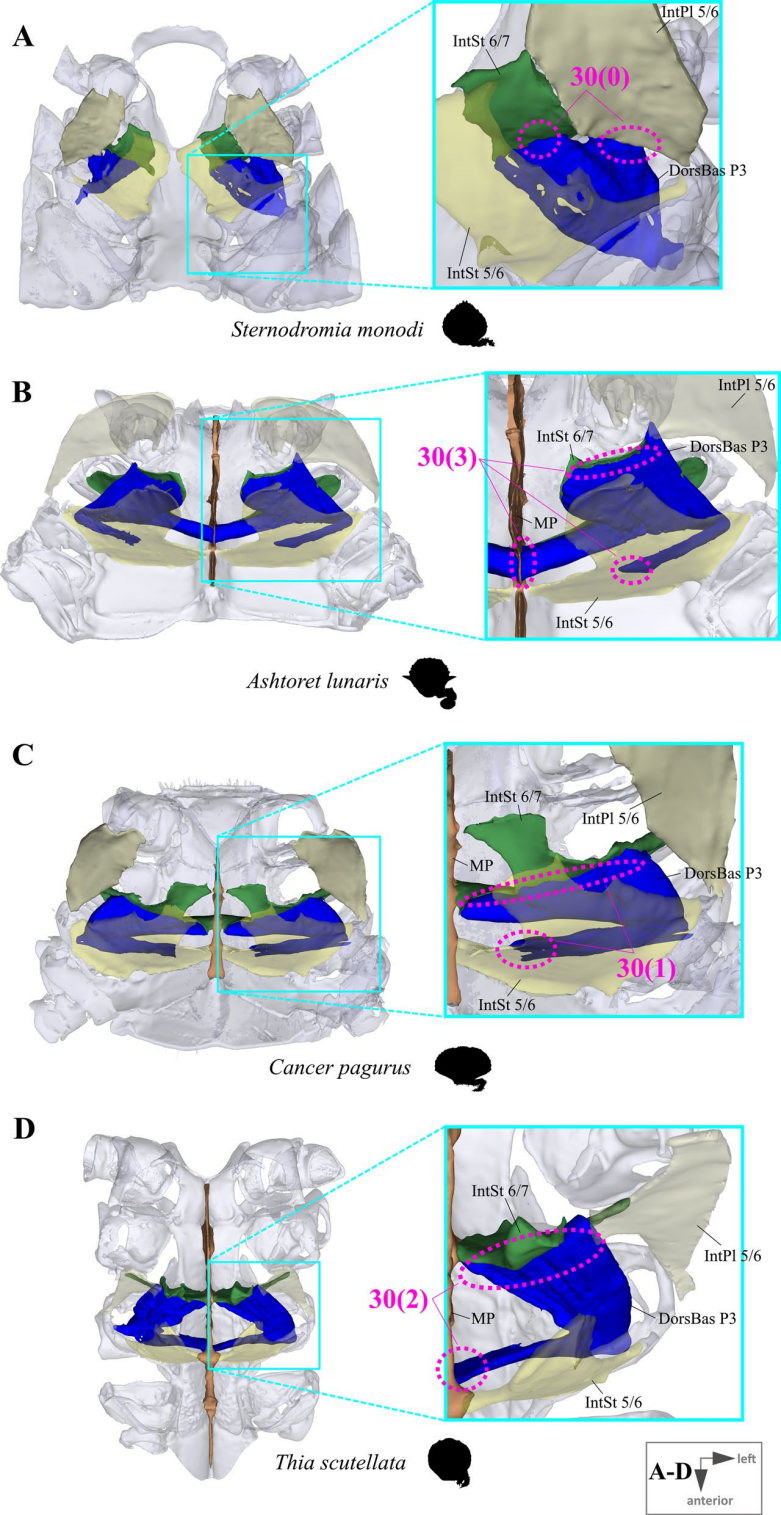
Examples showing character states concerned with the origin of the dorsal basi-ischium muscle of the 3rd pereiopod (character 30). **A** Dromiidae (Podotremata), **B** Matutidae (Calappoidea), **C** Cancridae (Cancroidea), **D** Carcinidae (Portunoidea). *DorsBas P3* 3rd pereiopod dorsal basi-ischium muscle, *IntPl 6/7* interopleurite between thoracomeres 6 and 7, *IntSt* interosternite (with number pair indicating thoracomeres between which it is situated), *MP* median plate

**Fig. 24:**
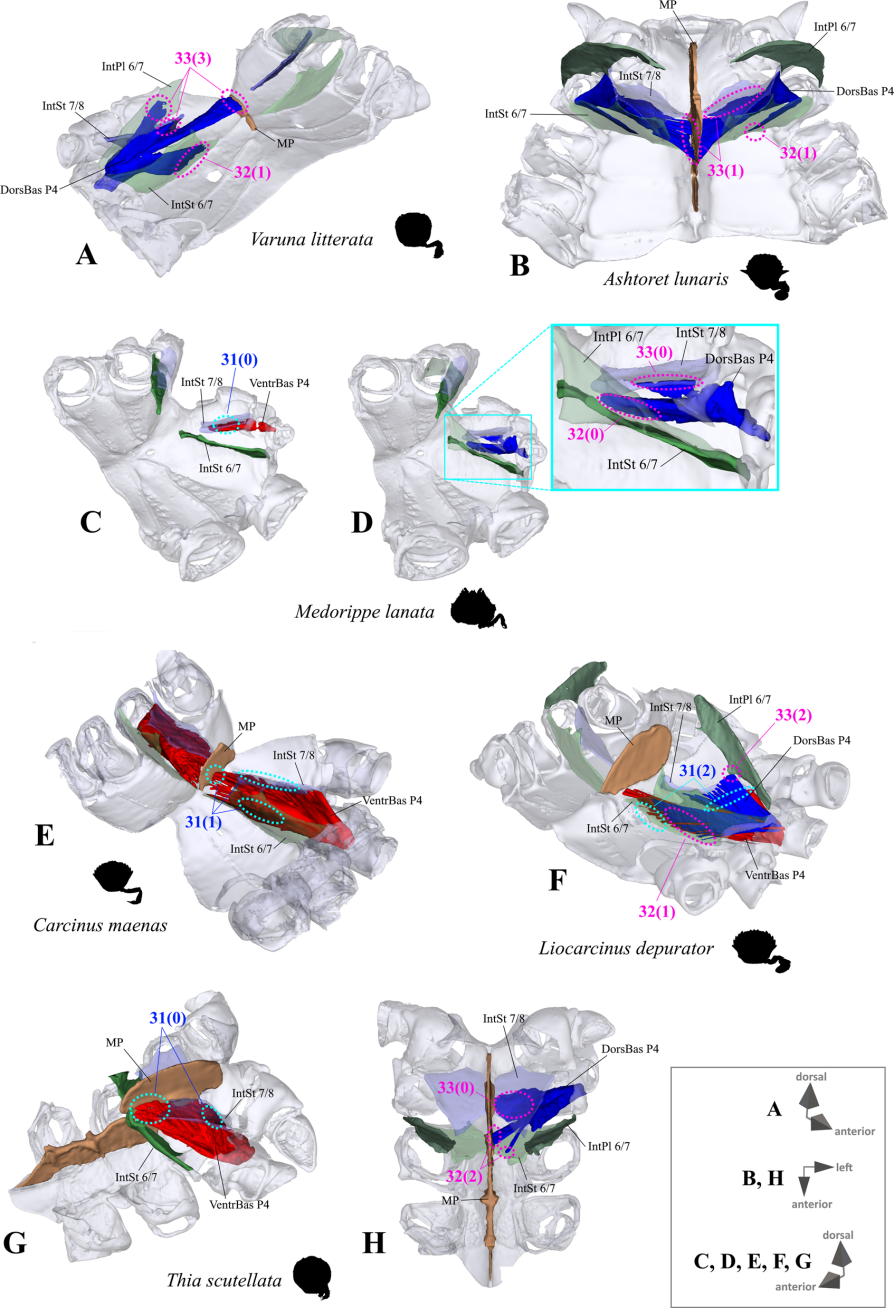
Examples showing character states concerned with the origin of the dorsal and ventral basi-ischium muscles of the 4th pereiopod (character 28) in axial skeletons seen from different perspectives. **A** Varunidae (Thoracotremata), **B** Matutidae (Calappoidea), **C, D** Dorippidae (Dorippoidea), **E–H** Carcinidae (Portunoidea). *DorsBas P4* 4th pereiopod dorsal basi-ischium muscle, *IntPl 6/7* interopleurite between thoracomeres 6 and 7, *IntSt* interosternite (with number pair indicating thoracomeres between which it is situated), *MP* median plate, *VentrBas P4* 4th pereiopod ventral basi-ischium muscle

*Extrinsic musculature of pereiopods 1–4* In contrast to the extrinsic musculature of P5 (see below), differences in the origin positions of the extrinsic musculature of P1–P4 in the taxa examined are limited in most cases to single terminals. We found no apparent differences between species in the origins of the extrinsic musculature of P1. In all taxa, the P2 anterior coxa muscle originates at interosternite 4/5, interosternite 5/6 and interopleurite 4/5 (character state 25(0); Fig. 19A, B), while the P3 anterior coxa muscle originates at interosternite 5/6, interosternite 6/7 and interopleurite 5/6 (Fig. 19C). Only in *Ovalipes ocellatus* were parts of the P2 anterior coxa muscle additionally found to originate at interopleurite 3/4 (character state 25(1); Fig. 19D). In most of the species, the P2 and P3 anterior coxa muscles have a branch which originates at interopleurite 4/5 and 5/6, respectively, and runs along (but does not attach at) the medial side of the junction plate (Fig. 19A, C). This branch was not found in some taxa (*Sternodromia monodi, Medorippe lanata, Telmessus cheiragonus, Corystes cassivelaunus, O. ocellatus, Lissocarcinus orbicularis* Dana, 1852*, Catoptrus nitidus* A. Milne-Edwards, 1870), but since it is very thin and the musculature in the voucher material representing these taxa is in parts poorly preserved, this might be artificial. We thus do not implement these findings in the character statements. In both *T. cheiragonus* and *C. cassivelaunus* the P2 posterior coxa muscle originates at interopleurite 4/5 and 5/6 (character state 26(1); Fig. 20A), while *Libystes nitidus* A. Milne-Edwards, 1867 is the only representative in which the muscle originates at interosternite 6/7 in addition to interopleurite 5/6 (character state 26(2); Fig. 20C). In all other species, it originates at interopleurite 5/6 alone (character state 26(0); Fig. 20B). The P2 dorsal basi-ischium muscle originates at interosternite 4/5 and 5/6 in all terminals, but in *Thia scutellata* and *Ashtoret lunaris*, it additionally originates at the median plate, which is conceptualized here as a neomorphic character (character 27; Fig. 20D, E) that is scored as inapplicable in taxa in which the median plate is absent or does not extend to pereiomere 2/thoracomere 5 (character states 7(0), 8(1) or 8(2)). In all taxa in which the median plate reaches thoracomere 5, the P3 and P4 ventral basi-ischium muscle originates at the median plate and interosternites 4/5 and 5/6, respectively. However, *T. scutellata* differs from the other taxa in that the P2 ventral basi-ischium muscle does not originate at interosternite 4/5 and the P3 muscle does not originate at interosternite 5/6 (the anterior interosternite of the respective thoracomere; character state 28(1); Fig. 21D). Interestingly, this is also the case in the outgroup taxon *S. monodi* (character state 28(0); Fig. 21A). Both species of *Cancer* Linnaeus 1758 and *Libystes nitidus* are the only taxa in which the P2 and P3 ventral basi-ischium muscles originate both at the anterior and posterior interosternites of the respective thoracomere (interosternites 4/5, 5/6 and 6/7), but not at interopleurite 5/6 or interopleurite 6/7, respectively, as in most other species (character states 28(3) and 28(4); Fig. 21B, D). All taxa have a P3 posterior coxa muscle which originates at interopleurite 6/7 (character state 29(0); Fig. 22B). *L. nitidus* again is the only species to have an additional branch which originates at interosternite 6/7, remotely from the junction plate (character state 29(2); Fig. 22C), while in *L. nitidus, C. cassivelaunus, A. lunaris, Macropipus rugosus* and *Bathynectes maravigna,* the P3 posterior coxa muscle also originates at interosternite 7/8 (character state 29(1); Fig. 22A). A P3 dorsal basi-ischium muscle at interosternite 6/7 only is exclusive to *S. monodi* (character state 30(0); Fig. 23A). *A. lunaris* and *T. scutellata* are the only taxa in which the P3 dorsal basi-ischium muscle originates at the median plate, with the muscle additionally originating at interosternite 5/6 in *A. lunaris* and *C. cassivelaunus* only (character states 30(2), 30(3); Fig. 23B, D). In all the other taxa the muscle originates at interosternite 5/6 and 6/7, but not at the median plate (character state 30(1); Fig. 23C), and additionally at the junction plate, near interopleurite 5/6, sometimes with some fibre bundles attached to it. However, as the attachment sites at interopleurite 5/6 are ambiguous in many specimens of different species, they are not included in the character statements. The origin positions of the P4 ventral basi-ischium muscle (character 31) in species in which the median plate reaches interosternites 5/6 or 6/7 (character states 8(1) and 8(2)) largely depend on whether the whole median plate is occupied by the P5 extrinsic musculature, which is the case in all the typical P5-swimming crabs (assigned to the morphotype on the basis of the criteria mentioned above, including a short merus; character state 31(2); Fig. 24F) except “*Polybius” henslowii*. In the latter and all the other species in which a median plate is present (character state 7(1)), part of the P4 ventral basi-ischium muscle also originates at the median plate (character state 31(1); Fig. 24E). However, in *“Polybius” henslowii*, only a small fringe of the median plate is covered by the muscle. *T. scutellata* is the only ingroup taxon in which the P4 ventral basi-ischium muscle does not originate at interosternite 6/7, which again it has in common with outgroup taxa *S. monodi* and *M. lanata* (character state 31(0); Fig. 24C, G). With regard to the P4 dorsal basi-ischium-muscle, a distinct anterior and posterior branch can be distinguished in all species. The anterior branch always originates at interosternite 6/7 only (character state 32(1); Fig. 24B, F, H), except in *S. monodia* and *M. lanata*, in which it has an origin at the sternum (character state 32(0); Fig. 24D) and *T. scutellata*, in which it additionally originates at the median plate (character state 32(2); Fig. 24H). In all taxa, the posterior branch originates at interosternite 7/8, in *Calappa granulata, A. lunaris, Cancer pagurus* and *C. cassivelaunus* it originates additionally at the median plate (character state 33(1); Fig. 24B) and in several species it originates additionally not at the median plate but at interopleurite 6/7 (character 33(2), Fig. 24F). In *Eriocheir sinensis* and *Varuna litterata* the posterior branch additionally originates at both the median plate and interopleurite 6/7 (character state 33(3); Fig. 24A).

Extrinsic musculature origins at the junction plate are not considered in characters 25–33 because, as the junction plate is a product of the fusion of interosternites and interopleurites, with no visible sutures, muscular attachment sites here are ambiguous.

25. Pereiopod 2, anterior coxa muscle, origin (Fig. 19): at interosternites 4/5 + 5/6 + interopleurite 4/5 (0); at interosternites 4/5 + 5/6 + interopleurites 3/4 + 4/5, (1).
26. Pereiopod 2, posterior coxa muscle, origin (Fig. 20A–C): at interopleurite 5/6 (0); at interopleurites 4/5 + 5/6 (1); at interosternite 6/7 + interopleurite 5/6 (2).
27. Pereiopod 2, dorsal basi-ischium muscle, origin at median plate (additional to interosternites 4/5 & 5/6; Fig. 20D, E): absent (0); present (1); inapplicable (–) if 7(0), 8(1), 8(2), 9(1) or 9(2).
28. Pereiopod 2, 3, ventral basi-ischium muscle, origin (Fig. 21): at sternum + posterior interosternite of respective thoracomere + pleurum (0); at sternum + median plate + posterior interosternite of respective thoracomere (1); at sternum + median plate + anterior interosternite of respective thoracomere, (2); at sternum + median plate (if extending up to respective thoracomere) + anterior interosternite of respective thoracomere + posterior interosternite of respective thoracomere, (3) at sternum + median plate (if present and extending up to respective thoracomere) + anterior interosternite of respective thoracomere + posterior interosternite of respective thoracomere, + posterior interopleurite of respective thoracomere, (4).
29. Pereiopod 3, posterior coxa muscle, origin (Fig. 22): at interopleurite 6/7 (0); at interosternite 7/8 + interopleurite 6/7 (1); at interosternite 6/7 + 7/8 + interopleurite 6/7 (2).
30. Pereiopod 3, dorsal basi-ischium muscle, origin (Fig. 23): at interosternite 6/7 + interopleurite 5/6 (0); at interosternites 5/6 + 6/7 (1); at median plate + interosternite 6/7 (2); at median plate + interosternites 5/6 + 6/7 (3).
31. Pereiopod 4, ventral basi-ischium muscle, origin (Fig. 24C, E, F, G): at median plate (if present) + interosternite 7/8 (0); at median plate + interosternites 6/7 + 7/8 (1); at interosternites 6/7 + 7/8 (2).
32. Pereiopod 4, dorsal basi-ischium muscle, anterior branch, origin (Fig. 24A, B, D, F, H): at sternum (0); at interosternite 6/7 (1); at median plate + interosternite 6/7 (2);
33. Pereiopod 4, dorsal basi-ischium muscle, posterior branch, origin (Fig. 24A, B, D, F, H): at interosternite 7/8 (0); at median plate + interosternite 7/8 (1); at interosternite 7/8 + interopleurite 6/7 (2); at median plate + interosternite 7/8 + interopleurite 6/7 (3).

**Fig. 25:**
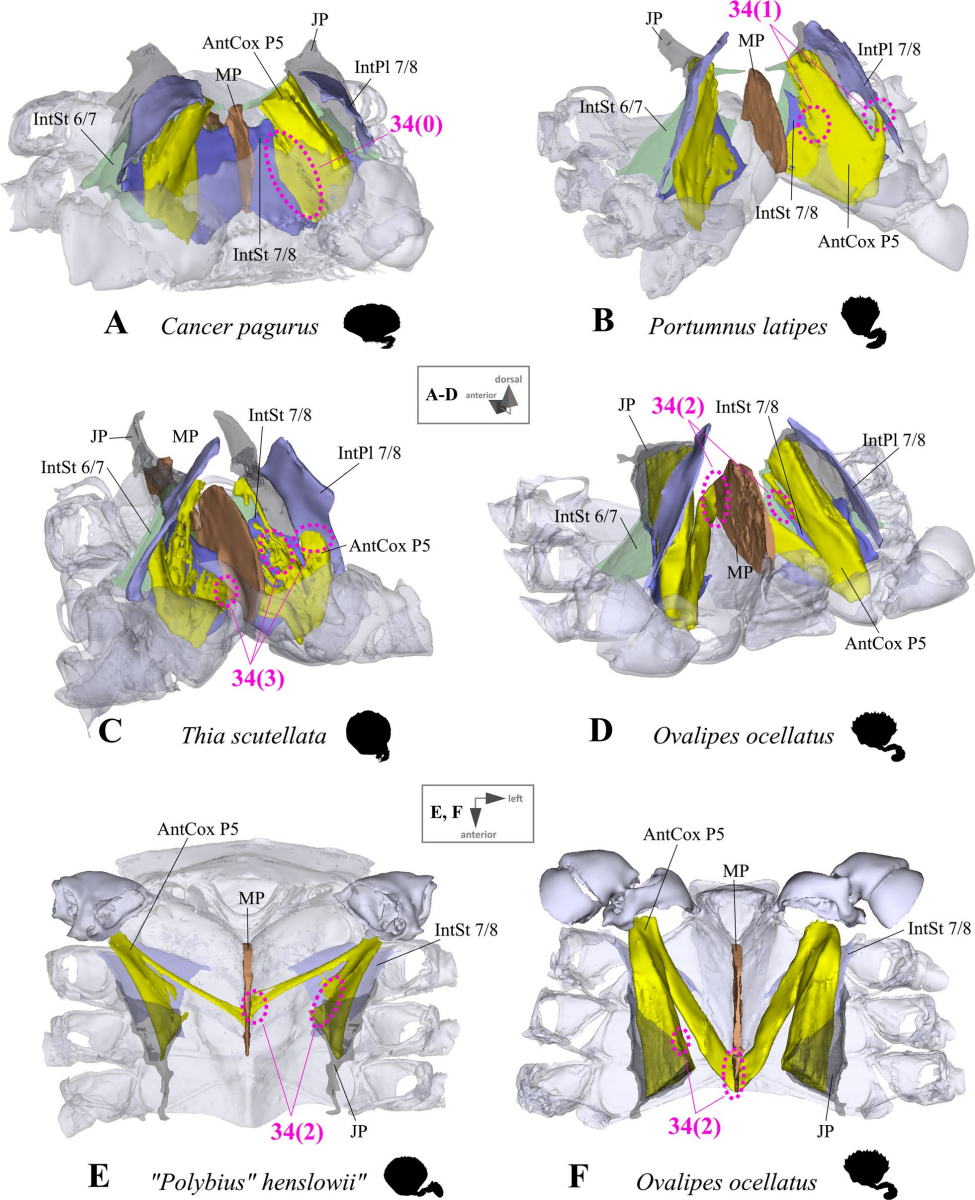
Examples showing character states concerned with the origin of the anterior coxa muscle of the 5th pereiopod (character 34) in axial skeletons seen from posterio-dorsal (**A**–**D**) and from dorsal (**E**, **F**). Note that in the P5-swimmers (**E**, **F**), long muscle fibres originate at the median plate, and that there are also shorter fibres originating at the median plate in *Thia* (**C**). *AntCox P5* 5th pereiopod anterior coxa muscle, *IntPl 7/8* interopleurite between thoracomeres 7 and 8, *IntSt* interosternite (with number pair indicating thoracomeres between which it is situated), *JP* junction plate, *MP* median plate

**Fig. 26:**
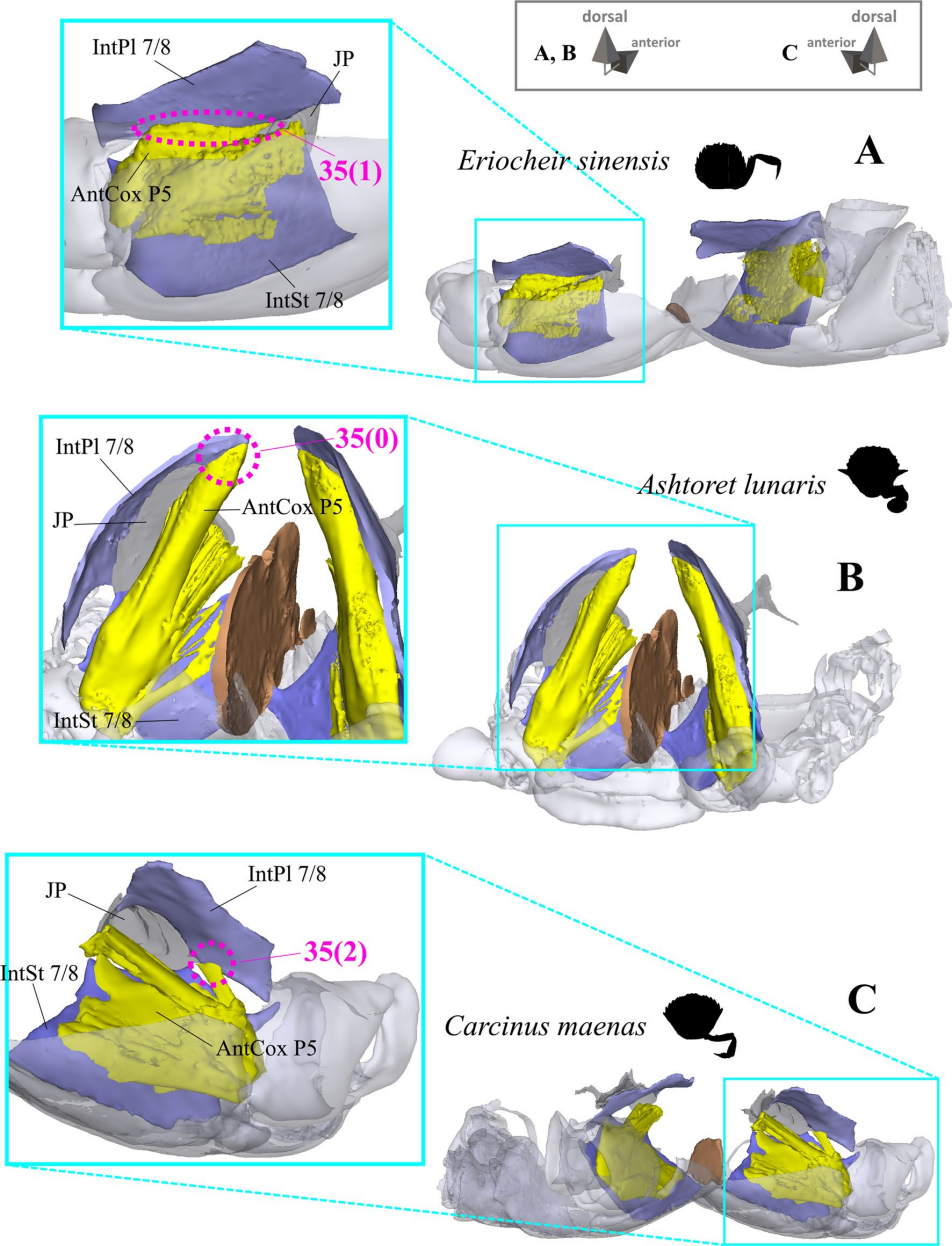
Examples showing character states concerned with the position of the origin of the anterior coxa muscle of the 5th pereiopod (character 35) in axial skeletons seen from different perspectives. **A** Varunidae (Thoracotremata), **B** Matutidae (Calappoidea), **C** Carcinidae (Portunoidea). *AntCox P5* 5th pereiopod anterior coxa muscle, *IntPl 7/8* interopleurite between thoracomeres 7 and 8, *IntSt 7/8* interosternite between thoracomeres 7 and 8, *JP* junction plate

**Fig. 27:**
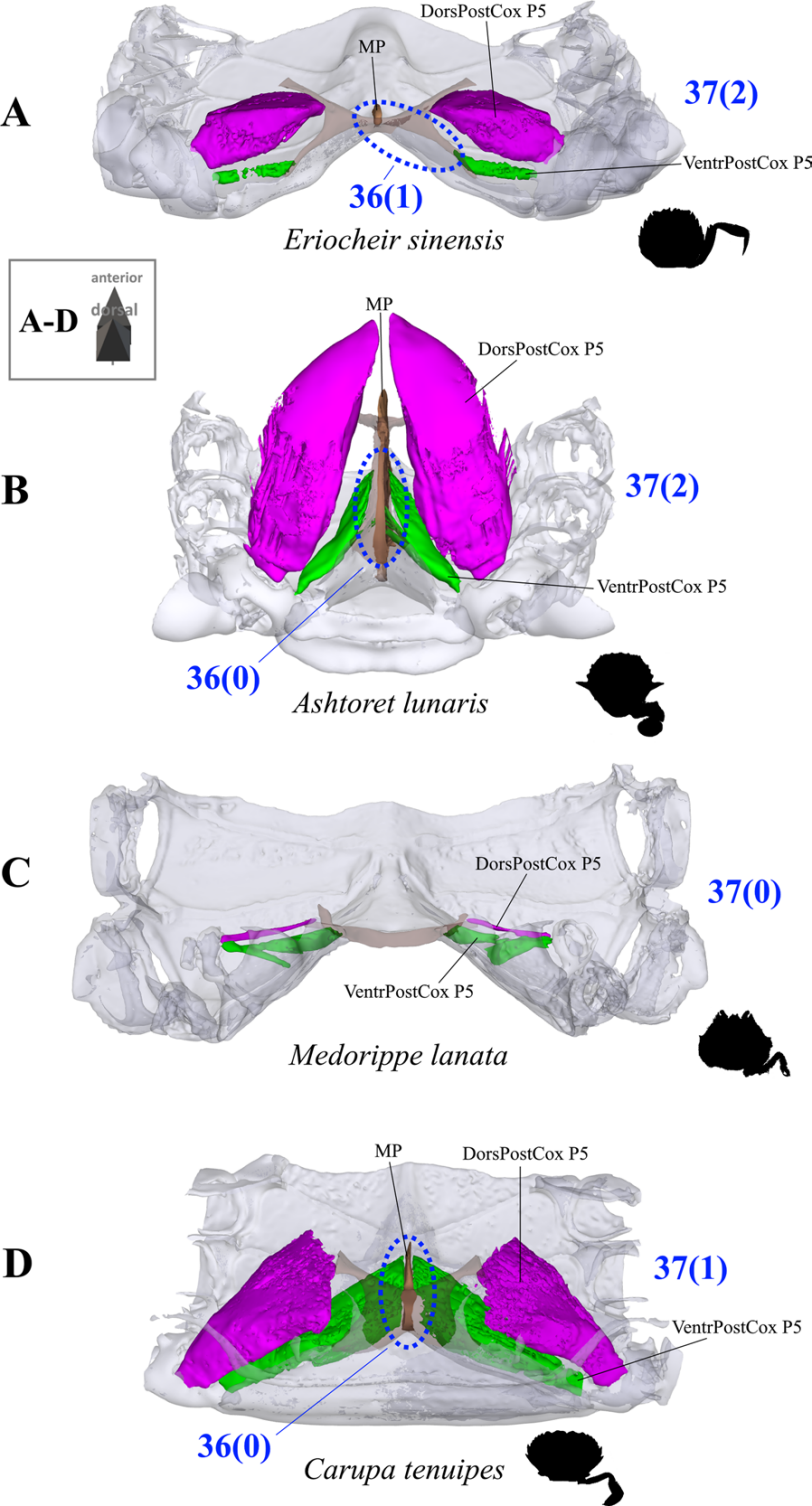
Examples showing character states concerned with the origin of the ventral posterior coxa muscle of the 5th pereiopod (character 36) and the relative volume of the dorsal posterior coxa muscle of the 5th pereiopod compared to the ventral in axial skeletons seen from posterio-dorsal. **A** Varunidae (Thoracotremata), **B** Matutidae (Calappoidea), **C** Dorippidae (Dorippoidea), **D** Portunidae (Portunoidea). *DorsPostCox P5* 5th pereiopod dorsal posterior coxa muscle, *MP* median plate, *VentrPostCox P5* 5th pereiopod ventral posterior coxa muscle

**Fig. 28:**
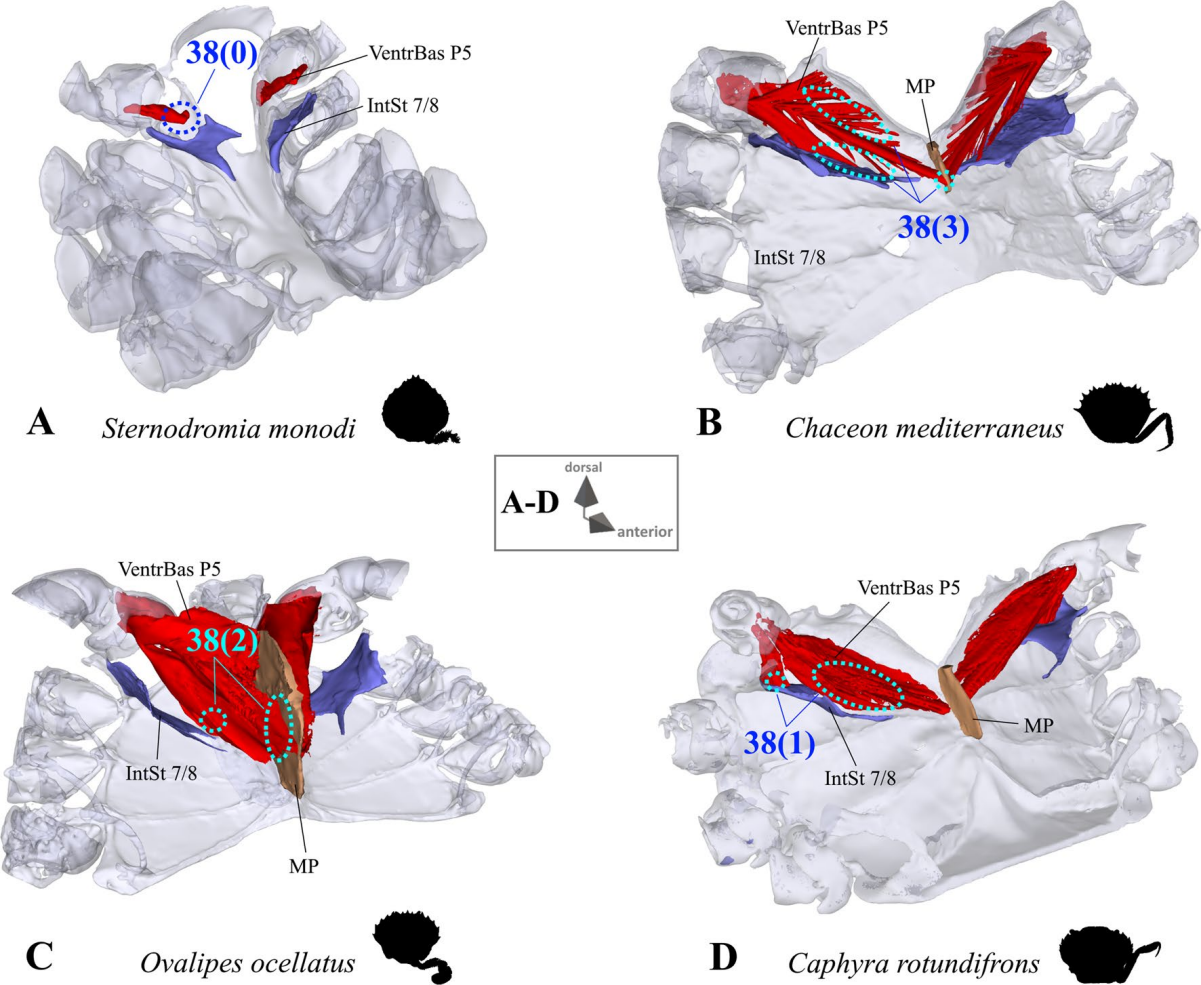
Examples showing character states concerned with the origin of the ventral basi-ischium muscle of the 5th pereiopod (character 38) in axial skeletons seen from anterio-dorsal. **A** Dromiidae (Podotremata), **B**, **C** Geryonidae, **D** Portunidae (Portunoidea). *IntSt 7/8* interosternite between thoracomeres 7 and 8, *MP* median plate, *VentrBas P5* 5th pereiopod ventral basi-ischium muscle

**Fig. 29:**
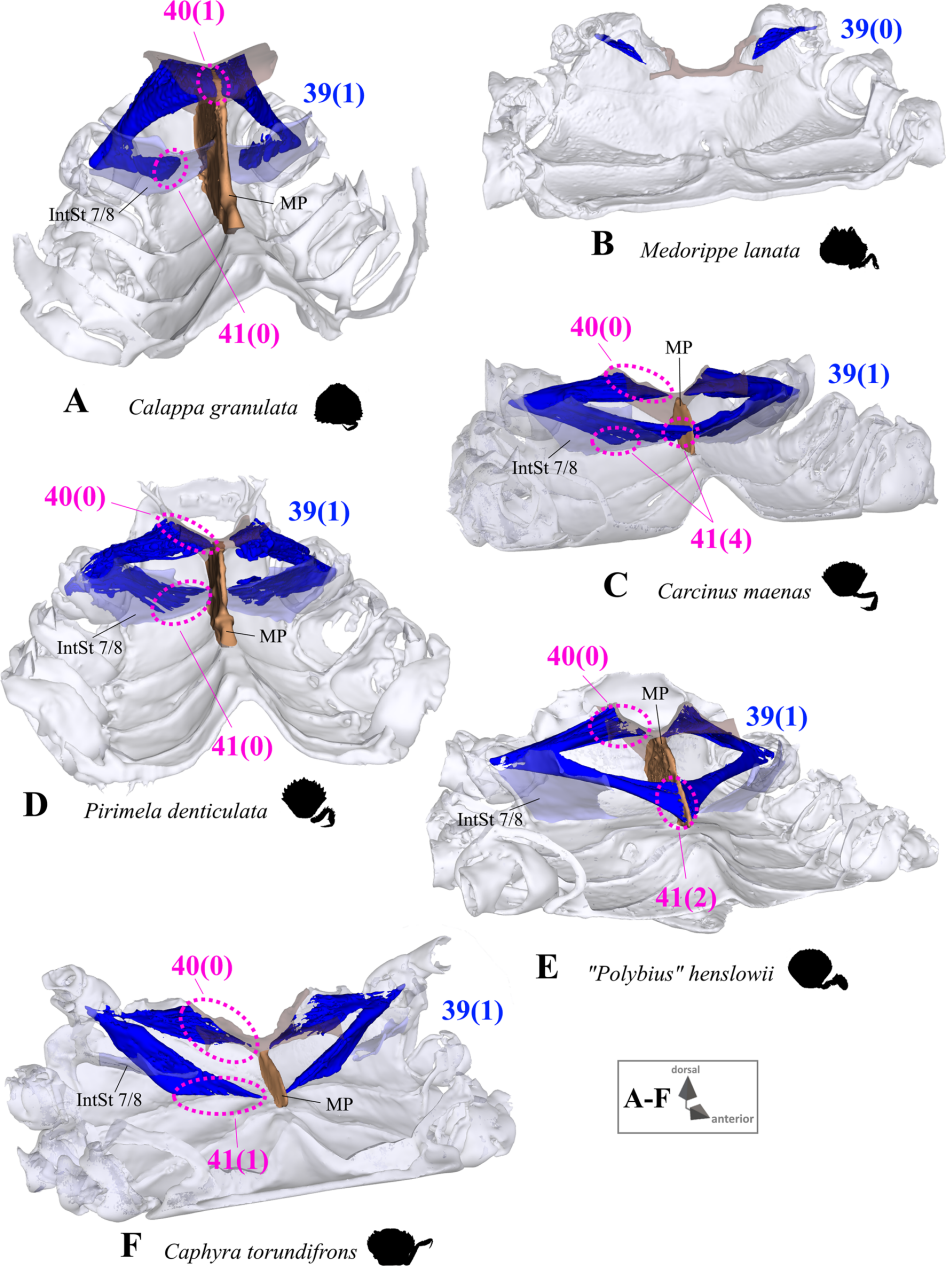
Examples showing character states concerned with the configuration of the ventral basi-ischium muscle of the 5th pereiopod (character 39), as well as the origin of its dorsal (character 40) and ventral (character 41) branch (if present) in axial skeletons seen from anterio-dorsal. **A** Calappidae (Calappoidea), **B** Dorippidae (Dorippoidea); **C**-**E** Carcinidae (Portunoidea), **F** Portunidae (Portunoidea). *DorsalBas P5* 5th pereiopod dorsal basi-ischium muscle, *IntSt 7/8* interosternite between thoracomeres 7 and 8, *MP* median plate

*Extrinsic musculature of pereiopod 5* The extrinsic musculature of P5 is especially interesting as it is responsible for moving the swimming leg in P5-swimmers. Remarkably, in all P5-swimming crabs a branch of the anterior coxa muscle originates at the median plate. The muscle fibres in this branch are long because of the distance between the insertion point at the coxa and the origin at the median plate or its anterior process (which is present in all typical P5-swimmers assigned to the morphotype on the basis of the criteria mentioned above; Fig. 25D–F). In some taxa, several distal fibre bundles of the anterior coxa muscle can be distinguished as having their origin at interopleurite 7/8 (character states 34(1), 34(3); Figs. 25B, C, 26). However, the exact attachment positions vary between the species. They may be at the upper end of interopleurite 7/8 as in *Medorippe lanata, Calappa granulata* and *Ashtoret lunaris* (in which the fibre bundles form a voluminous branch; character state 35(0); Fig. 26B), in the centre of interopleurite 7/8 as in *Eriocheir sinensis* and *Varuna litterata* (character state 35(1); Fig. 26A), or at the lower end as in the other species (character state 35(2); Fig. 26C). A ventral posterior coxa muscle originating at the median plate is present in all the taxa in which a median plate is present (character state 36(0); Fig. 27B, D) except *E. sinensis* and *V. litterata* (character state 36(1); Fig. 27A). The volume of the ventral posterior coxa muscle differs significantly from that of the dorsal posterior coxa muscle in several species (character states 37(0), 37(2); Fig. 27A–C). In most species, the ventral basi-ischium muscle originates at the sternum, the median plate and interosternite 7/8 (character state 38(3); Fig. 28B). In *Thia scutellata* and *Ovalipes ocellatus,* it originates solely at the sternum and the median plate (character state 38(2); Fig. 28C), while in *Libystes nitidus* and both species of *Caphyra* Guérin, 1832*,* it originates at the sternum and interosternite 7/8 only (character state 38(1); Fig. 28D). In outgroup taxa lacking a median plate, the ventral basi-ischium muscle only originates at the sternum (character state 38(0); Fig. 28A). With regard to the dorsal basi-ischium muscle, in all taxa but *Sternodromia monodi* and *M. lanata*, two distinct branches can be distinguished (character 39; Fig. 29), with the dorsal branch always originating at the sella turcica and in some species additionally at the sternum near the border between the sella turcica and the sternum (as the border is indistinct this is not considered a separate character state). In *C. granulata* and *A. lunaris* only, the dorsal branch also originates at the median plate (character state 40(1); Fig. 29A). The ventral branch can originate at interosternite 7/8 only (character state 41(0); Fig. 29A, D), at the median plate only (state 41(2); Fig. 29E), and at both interosternite 7/8 and the median plate (state 41(4); Fig. 29C). In *L. nitidus,* the ventral branch originates at the sternum and interosternite 7/8 (character state 41(3)), while in both species of *Caphyra,* it only originates directly at the sternum (state 41(1); Fig. 29F).

In typical P5-swimming crabs (assigned to the morphotype on the basis of the criteria mentioned above), the extrinsic musculature of P5 is considerably more voluminous than that of P2–P4. Voluminous musculature is also present in *A. lunaris* and *Coelocarcinus foliatus* Edmondson, 1930*,* though here it is similarly expanded anteriorly and dorsally, while in the P5-swimmers it is mainly expanded anteriorly. The expansion of musculature is associated with specific features of the axial skeleton, such as the anteriorly expanding process of the median plate (characters 9 and 10) in P5-swimmers. The dimension of the extrinsic musculature of P5 is thus not considered an independent character here.

As is the case with the extrinsic musculature associated with P2–P4, origins at the junction plate are not considered in characters 34–41.

34. Pereiopod 5, anterior coxa muscle, origin (Fig. 25): at interosternite 7/8(0); at interosternite 7/8 + interopleurite 7/8 (1); at median plate + interosternite 7/8 (2); at median plate + interosternite 7/8 + interopleurite 7/8 (3).
35. Pereiopod 5, anterior coxa muscle, origin at interopleurite 7/8, position (Fig. 26): at upper end of interopleurite 7/8 (0); at centre of interopleurite 7/8 (1); at lower end of interopleurite 7/8 (2); inapplicable (–) if 34(0) or 34(2).
36. Pereiopod 5, ventral posterior coxa muscle, origin at median plate (additional to sternum/sella turcica; Fig. 27): present (0); absent (1), inapplicable (–) if 7(0).
37. Pereiopod 5, ventral posterior coxa muscle, volume compared to that of dorsal posterior coxa muscle (Fig. 27): greater (0); similar (1); smaller (2).
38. Pereiopod 5, ventral basi-ischium muscle, origin (Fig. 28): at sternum (0); at sternum + interosternite 7/8(1); at sternum + median plate (2); at sternum + median plate + interosternite 7/8 (3).
39. Pereiopod 5, dorsal basi-ischium muscle, configuration (Fig. 29): without distinct branches (0); separated into a ventral and a dorsal branch (1)
40. Pereiopod 5, dorsal basi-ischium muscle, dorsal branch, origin at median plate (in addition to sternum/sella turcica; Fig. 29): absent (0); present (1); inapplicable (–) if 7(0) or 39(0).
41. Pereiopod 5, dorsal basi-ischium muscle, ventral branch, origin (Fig. 29): at interosternite 7/8 (0); at sternum (1); at median plate (2); at sternum + interosternite 7/8 (3); at median plate + interosternite 7/8 (4) inapplicable (–) if 39(0).

#### Characters concerned with the external morphology of pereiopod 5

**Fig. 30:**
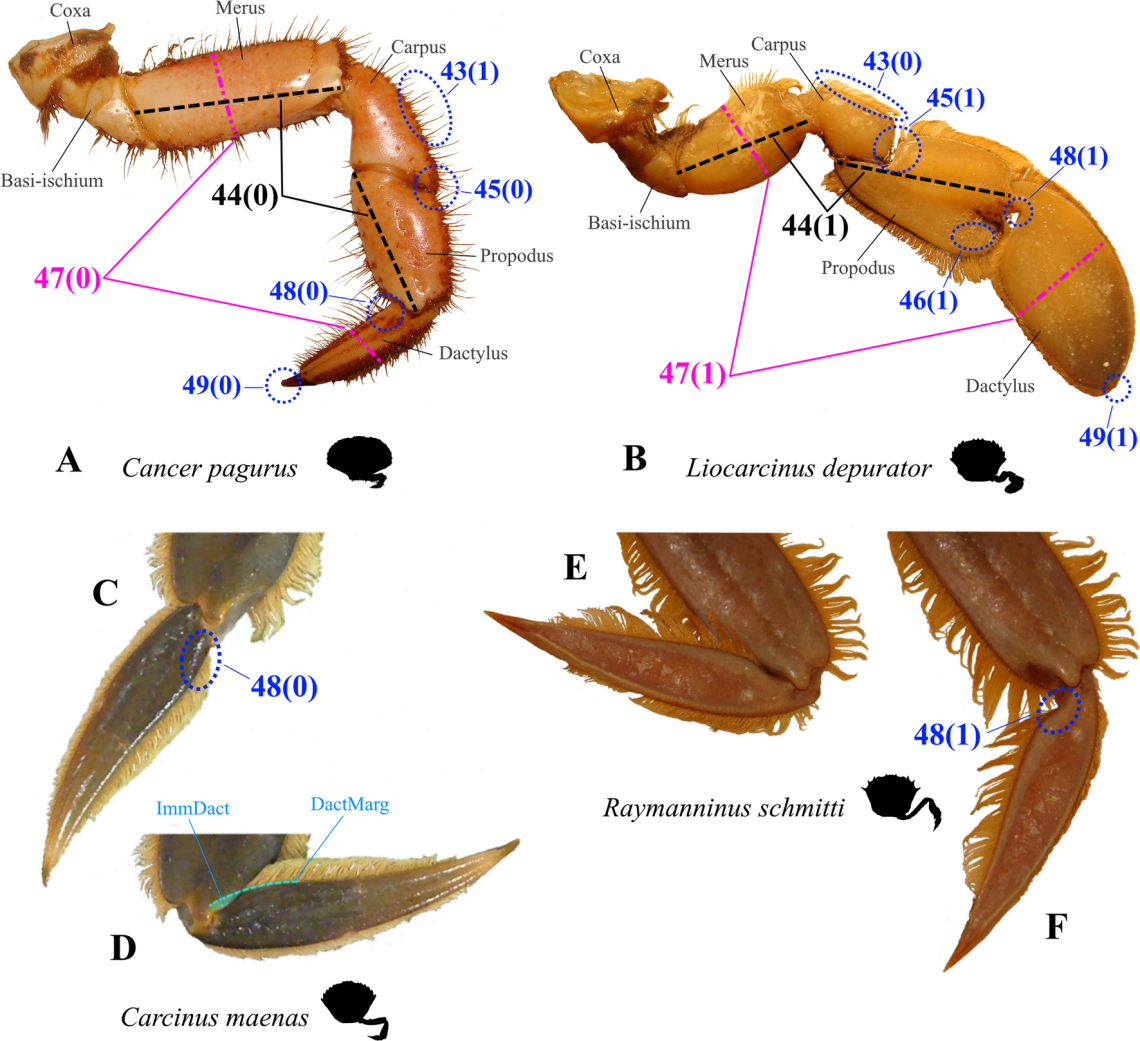
Examples showing character states concerned with the exterior of the 5th pereiopod. **A**, **B** Comparison between walking leg-shaped 5th pereiopod (**A**) and swimming leg-shaped 5th pereiopod (**B**). Note that character state 43(1) shown here which is concerned with the arrangement of long setae is not necessarily typical of a walking leg, and those concerning merus length (44(1)) and shape of the dactylus tip (49(1)) are not necessarily typical of a swimming leg (see also Figs. 34, 35, and “Characterising the P5-swimming crab morphotype” section). **C**–**F** Examples showing character states concerned with the shape of the margin of the proximo-ventral dactylus of the 5th pereiopod (character 48). Note that when the dactylus is adducted (**D**, **E**) and the proximo-ventral dactylus margin is convex (or straight) (**C**), the dactylus is partly immersed in the arthrodial cavity of the propodus (**D**), while it is not immersed (**E**) when the proximo-ventral dactylus margin is concave (**F**). *DactMarg* proximo-ventral dactylus margin, *ImmDact* area of the dactylus that is immersed in the arthrodial cavity of the propodus when the dactylus is adducted

There is much variability between species in the external features of pereiopod 5. The occurrence and modes of arrangement of long setae on P5 can be seen in Figs. 1, 2, 3, 4, 5, 6 and 30A, B. In most species, long setae are arranged in dense fringes along podomere margins, as is also the case in a typical swimming leg (character state 43(0); Fig. 30B). We here express the shortened merus of a typical swimming leg through a comparison to P5 propodus length (character state 44(1); Fig. 30B). The latter has been shown to be relatively independent of merus length [52]. The insertion of the propodus in the disto-dorsal carpus margin is associated with an alteration in the position of the carpal-propodal articulation axis relative to the longitudinal propodus axis (character state 45(1); Fig. 30B; see also [22, 29]). This state is present in all typical P5 swimmers, but also (with varying distinctness) in some other taxa.

**Fig. 31:**
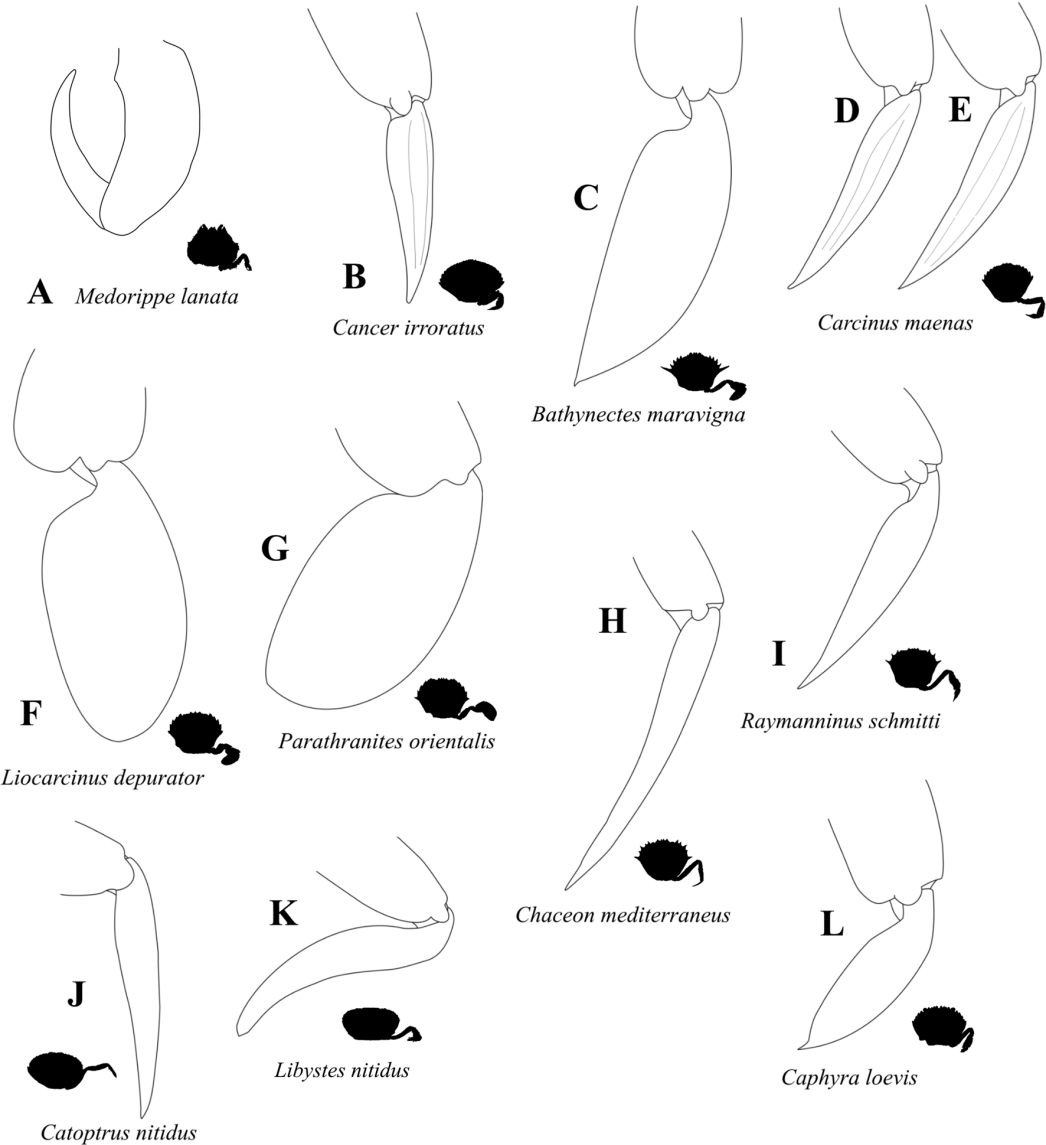
Drawings showing variability in the shape of the dactylus of the right 5th pereiopod. **A** Dorippidae (Dorippoidea), **B** Cancridae (Cancroidea), **C**-**G** Carcinidae (Portunoidea), **H**, **I** Geryonidae (Portunoidea), **J**-**L** Portunidae (Portunoidea)

With regard to P5 dactylus shape, we here prefer a character concept with fewer states than in Karasawa et al. [26] as the variability of this character can be very high not only between species (Fig. 31) but also within species, implying that it is prone to subjectivity. For example, we found in *Carcinus maenas* that there were specimens that could be assigned to the state “ensiform” (Fig. 31D) based on Karasawa et al. [26], while others exhibited a “lanceolate” dactylus shape (Fig. 31E). Consequently, the character we conceptualize here has only two states reflecting a clearly apparent, intersubjective difference: whether or not the P5 dactylus is broader than the P5 merus (a broad “paddle-shaped” dactylus being character state 47(1); Figs. 30B, 31C, F, G). A second character related to dactylus shape concerns its proximo-ventral margin. In species in which the margin is concave, the disto-ventral propodus area is not immersed in the arthrodial cavity when adducted (character state 48(1); Fig. 30B, E, F; see also [22]). This is the case in all the broad, paddle-shaped dactyli, but also in some of the narrow ones (see below).

**Fig. 32:**
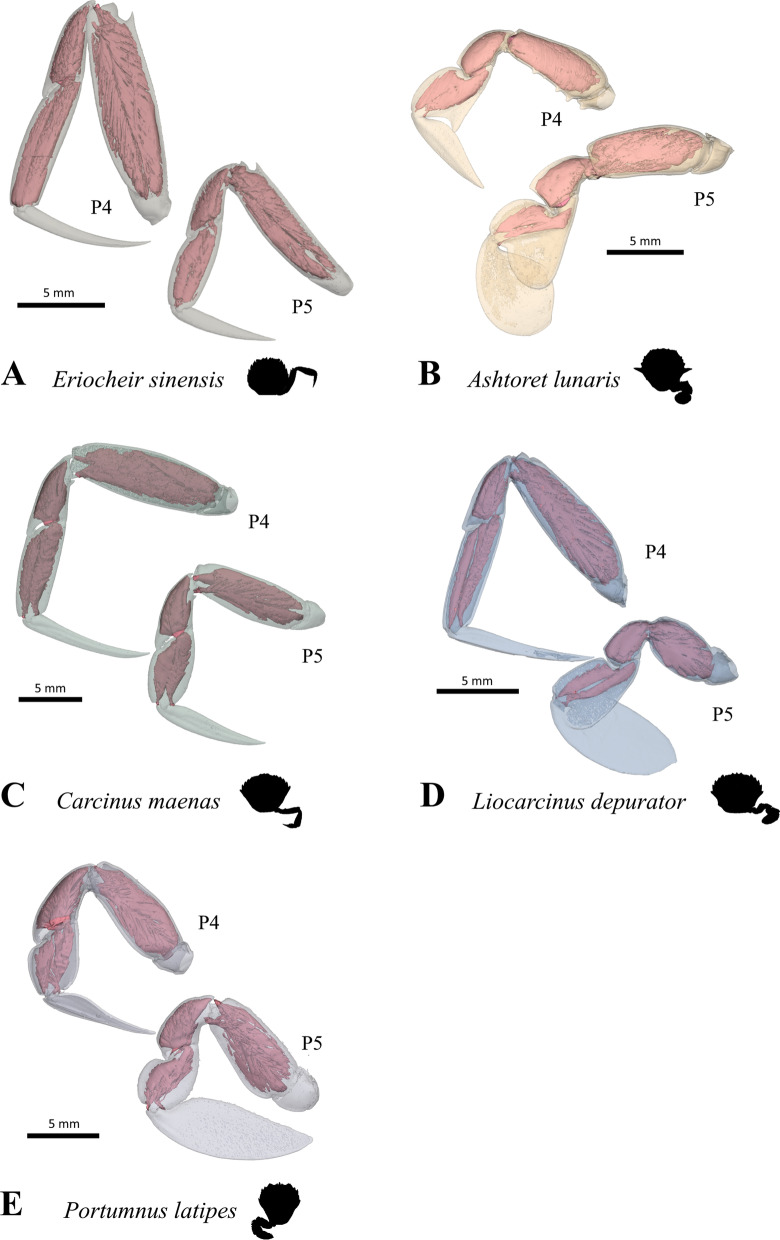
Representations of three-dimensional (3D) data showing variability in the shape of the left 4th (P4) and 5th (P5) pereiopods and their intrinsic musculature. **A** Varunidae (Thoracotremata), **B** Matutidae (Calappoidea), **C**-**E** Carcinidae (Portunoidea)

The P5 propodus and dactylus are broader than the respective P4 podomeres not only in taxa with a broad “paddle-shaped” dactylus*,* but also in *C. maenas* (albeit only slightly; Fig. 32C; see also for example [21, 22]). However, we do not conceptualize the P5 propodus and dactylus being broader than the respective P4 podomeres as an additional character herein. This is because in P5-swimming crabs and several other taxa with a paddle-like dactylus, the extreme broadening of the propodus is formed by a large lobe-like projection of the disto-ventral propodus margin (character state 46 (1); Figs. 30B, 32B, D) which is absent in *C. maenas*. In *C. maenas,* the broader propodus correlates with the relative volume of the musculature inserting at the dactylus, while in taxa with the propodus projection, this is not the case; i.e., the dactylus musculature is small in relation to propodus width (Fig. 32B, C, D). Furthermore, the P5 proximo-ventral dactylus margin in *C. maenas* is that of a normal walking leg, which means that the broader dactylus is not associated with a concave dactylus margin (Fig. 30C, D, 31D, E). For this reason, we consider the broader distal P5 podomeres in *C. maenas* not to be homologous to those in P5-swimmers (i.e. neither an identical character state nor a different state of the same character—in other words, not part at all of the transformation series suggested by Hartnoll [21]. Schäfer [51] also noted the P5 carpus in *C. maenas* to be broader than that of the P4, which again is the consequence of the relative volume of the musculature inserting at the propodus (Fig. 32C). We found slightly broader distal P5 podomeres (even less pronounced than in *C. maenas*) associated with relatively more voluminous intrinsic musculature also to be present in *Eriocheir sinensis* and *Cancer irroratus* (Fig. 32A). An examination of external morphological features in another species of *Eriochier* (*Eriocheir japonica* (De Haan 1835)) even showed distal P5 podomeres there to be broader (compared to P4 podomeres) to a smiliar degree to those in *C. maenas* (Fig. 36 in “Appendix”).

*Ashtoret lunaris, Bathynectes maravigna*, *Liocarcinus navigator, Carupa tenuipes* and *Lissocarcinus orbicularis* have a paddle-like dactylus like that found in typical P5-swimming crabs (character states 46(1), 47(1)), but a longer merus (character state 44(0); Figs. 2B, 4A, J, 6B, F, 32B)*. Coelocarcinus foliatus* has a paddle-shaped dactylus and a short P5 merus, (character state 44(1); Fig. 4C), but the merus is not significantly shorter than those of the other pereiopods (which are relatively short, too), and P5 lacks the long setae along the podomere fringes typical of a swimming leg. *Portumnus latipes* (Pennant, 1777) and *Xaiva biguttata* (Risso, 1816) are the only taxa in which the P5 dactylus is about as broad as in P5-swimmers (in *P. latipes* broader than in *X. biguttata*; character state 47(1)) without exhibiting a ventral propodus projection (character state 46(0); Figs. 4G, I, 32E). In *Caphyra loevis* (A. Milne-Edwards, 1869)*,* the dactylus is narrow (character state 47(0)) but has a concave proximo-ventral dactylus margin (character state 48(1)), while the propodus has a (small) ventral lobe-like projection (character state 46(1); Figs. 6F, 31L). *Raymanninus schmitti* (Rathbun, 1931) has a P5 propodus and dactylus that are not much broader than those of P4, but the dactylus has a concave proximo-ventral margin, a configuration which resembles that of a paddle-shaped dactylus (character state 48(1); Figs. 5C, 30E, F, 31I). A similar configuration is present in *Libystes nitidus* (Figs. 6D, 31K). All these taxa, and *Varuna litterata* (Fig. 1C), have a P5 disto-dorsal carpus insertion which receives the propodus (like in typical P5-swimmers; character state 45(1); Fig. 30B).

In *Sternodromia monodi*, *Medorippe lanata* and *Caphyra rotundifrons* (= *Trierarchus rotundifrons,* comb. nov. (A. Milne-Edwards, 1869))*,* the P5 propodus and dactylus form a subchela (Figs. 1A, 3, 6A, 31A). However, as its shape varies significantly between species it is not conceptualized as a united character (state) herein.

42. Pereiopod 5, long setae: present (0); absent (1).
43. Pereiopod 5, long setae, arrangement (Fig. 30A, B): arranged in dense fringes along podomere margins (0); arranged rather irregularly (1); inapplicable (–) if 42(1).
44. Pereiopod 5, merus, length relative to propodus (Fig. 30A, B): longer (0); equal or shorter (1).
45. Pereiopod 5, carpus, disto-dorsal margin, propodus insertion (Fig. 30A, B): absent (0); present (1).
46. Pereiopod 5, propodus, lobe-like expansion of postero-ventral margin (Fig. 30A, B): absent (0); present (1).
47. Pereiopod 5, dactylus, maximum width in relation to maximum width of P5 merus (Fig. 30A, B): smaller or equal (0); larger (1).
48. Pereiopod 5, dactylus, proximo-ventral margin, shape (Fig. 30): convex or straight (0); concave (1)).
49. Pereiopod 5, dactylus, tip, shape (Fig. 30A, B): pointed (0); rounded (1).

*Remarks on some morpheme properties mentioned by Hazerli and Richter* [22] Some unambiguous morphological differences in axial skeleton and external P5 morphology found by Hazerli and Richter ([22]; therein termed “morpheme properties”) of the non-swimmers *Cancer pagurus* and *Carcinus maenas* and the P5-swimming crab *Liocarcinus depurator* were not implemented in our character conceptualization here. The reason lies in the great morphological variety found in the larger taxon sampling of this work, which sometimes makes it difficult to determine discrete character states covering more than one species. The differences in question relate to the shape of the P5 basi-ischium, the size of the P5 thorax-coxa arthrodial cavity, the general volume of the extrinsic musculature of P5 (see also *Extrinsic musculature of pereiopod 5* in “Characters concerned with the axial skeleton and extrinsic musculature of pereiopods 2–5” section), the general width of thoracomere 8 together with the presence of an aliform pleural expansion of thoracomere 8 in *Liocarcinus depurator* (which is absent in *C. pagurus* and *C. maenas*), and the distances between the medial edges of interosternites 4/5 to 7/8 in a longitudinal plane (see morpheme properties 3, 4, 5, 11, 13, 14 and 23 in [22]). As mentioned above, we do not conceptualize the general broadening of the P5 propodus and dactylus as an additional character either (morpheme property 2 in [22]). This broadening is found in both *C. maenas* and *L. depurator* (in *C. maenas* to a much lesser degree; for a detailed explanation, see “Characters concerned with the external morphology of pereiopod 5” section). Measuring the angle of inclination of the P5 meral-carpal articulation and that between the P1 and P5 thorax-coxa joints (morpheme properties 6, 32) were beyond the scope of this work and thus not implemented either.

An extrinsic P5 anterior coxa muscle originating at interopleurite 6/7 (morpheme property 26 in [22]) is not considered in our conceptualization either since borders between the junction plate, interopleurite 6/7 and the sella turcica are not as apparent in many taxa as in those examined by Hazerli and Richter [22].

#### Other external characters

Several morphological features used in taxonomic approaches are not conceptualized as characters here because of their high variability between species. A discussion of this variability is beyond the scope of this paper, but suffice to say it makes the distinction of discrete states covering more than one species difficult (for a detailed discussion of many of the features in question, see [64]). They are concerned with the shape of the carapace front, the number of spines and fissures on the ventral orbit margin, the presence or absence of a spine or lobe at the inner angle of the dorsal orbit margin, the shape and number of anterolateral carapace spines, the presence or absence and configuration of carapace tubercles and ridges, the shape of the basal antenna article, the presence and shape of a “portunid lobe” on maxillipede 1 (touched upon by [66]) and the overall shape of the male pleon. Thus, only a few previously used external morphological characters are considered herein. Spiridonov et al. [63], who considered morphological features of the chelae an important character complex, described them in detail in many portunoid species but deduced no character concepts. We found a high degree of morphological chela variety in *Carcinus maenas* alone, with specimens exhibiting varying degrees of heterochely and heterodonty (Fig. 37A, B in “Appendix”). Homochelic specimens (homodontic or somewhat heterodontic) were observed too (Fig. 37C in “Appendix”). In *C. maenas*, this can perhaps be explained by morphological plasticity (see, for example, [3, 11]). As data concerning intraspecific variety are lacking for most of the other species examined herein, we did not implement chela morphology into the character matrix either.

Karasawa et al. [26] drew distinctions concerning not only the distinctiveness of the sutures between male pleomeres, but also concerning pleomere movability. As the movability was difficult to determine in conserved specimens, we only conceptualize suture distinctiveness here (character 53). We determined character states that were different from Karasawa et al. [26] regarding the mode of connection of the basal antenna article to the suborbital region in *Caphyra loevis, Raymanninus schmitti* and *Macropipus rugosus* (character 54)*.* The presence or absence of a subterminal spine on the first gonopod is adapted from Karasawa et al. [26], if data for the species were available (character 58).

Although we do not claim to present an exhaustive list of external morphological characters, we nevertheless conceptualize a few other conspicuous morphological differences as characters herein. They include (1) a putative respiratory canal formed by the prolongated endopodite of maxillipede 1 (character state 55; Fig. 38A–C in “Appendix”), which occurs in *Ashtoret lunaris*, *Calappa granulata* and *Medorippe lanata* (and constitutes a morphological feature that once was used to assign these species to Oxysytomata, a taxon that is widely refuted nowadays; [4, 10, 13]), and (2) the degree to which maxillipede 3 obscures the mandibles when adducted to cover the buccal cavern. This latter character constitutes an apparent morphological difference that separates the thoracotrematan from the podotrematan and heterotrematan taxa examined here (character 56; Fig. 38E, F in “Appendix”). With regard to external features of pereiopods 2–4, *Sternodromia monodi* and *M. lanata* differ from all other taxa by having a P4 which is less than half as long as P2 and P3 (with a coxa that is situated subdorsally; character state 57(0)). Gonopore positions are conceptualized and assigned to taxa on the basis of statements by Guinot et al. ([19]; character 59).

Images showing several of the characters listed as characters 50–59 and their states are supplied in the “Appendix”.

50. Carapace, maximum width in relation to maximum length: larger or equal (0); smaller (1).
51. Carapace, surface structure: smooth or only thinly covered with soft setae (0); velvety, densely covered with rigid setae (1).
52. Carapace, orbit, dorsal margin, surface structure (Fig. 39 in “Appendix”): with one fissure (0); with two fissures (1), with distinct spine (2); smooth (3).
53. Male pleon, distinctiveness of sutures: with distinct sutures between all pleomeres (0) with indistinct or no sutures between pleomeres 3–5 (1); with interrupted suture between pleomere 3 & 4 (2).
54. Antenna, basal article, mode of connection to suborbital region and epistome: articulated (0); confluent (1).
55. Maxillipede 1, endopodite (adducted), degree of closing of exhalant aperture (Fig. 38A–D in “Appendix”): not closing (0); completely closing except for small distal opening (1).
56. Maxillipede 3 (adducted), degree to which mandibles are covered (Fig. 38E, F in “Appendix”): completely covered, mandibles not visible (0); not completely covered, mandibles visible (1).
57. Pereiopod 4, length: less than half as long as P3 (0); similar to in P3 (1).
58. Gonopod 1, subterminal spines: absent (0); present (1).
59. Gonopore, position: coxal in females and males (0); sternal in females, coxal in males (1); sternal in both females and males (2).

## Results and discussion of phylogenetic analysis

**Fig. 33:**
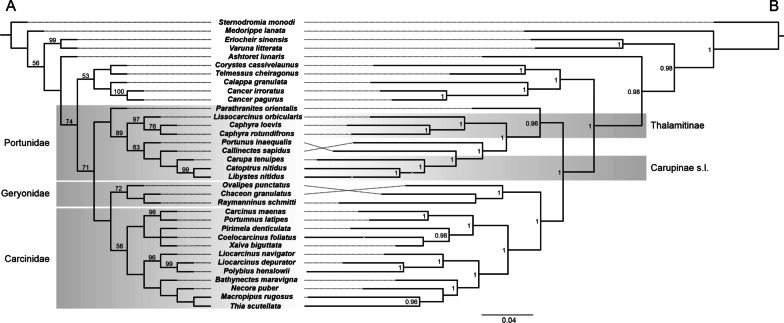
Phylograms of the combined data set of the 34 OTUs with higher classification and *Sternodromia monodi* set as outgroup. **A** Maximum parsimony (MP) phylogram calculated with TNT 1.5; strict consensus of the two most parsimonious trees; tree length: 6029; bootstrap values over 50 (1000 bootstrap replicates) are shown on the respective branch, **B** Bayesian inference (BI) phylogram calculated with MrBayes 3.2.7a; posterior probabilities > 0.95 are shown for the respective branches

Both phylogenetic analyses of the combined data set (BI and MP) resulted in nearly the same topologies (Fig. 33). In both analyses, the Portunoidea are recovered as monophyletic and composed of three distinct clades we herein name according to the classification system established by Evans [12]: Portunidae is the sister group to a taxon comprising the monophyletic Carcinidae and Geryonidae. The position of Portunidae deviates from the phylogenetic hypothesis put forward by Evans [12], in which Geryonidae is the sister group to Carcinidae and Portunidae.

In our analysis, *Parathranites orientalis* is part of the Portunidae, being a sister taxon to the remaining portunid representatives which is s consistent with morphological data showing that all representatives of Portunidae including *Parathranites orientalis* have a median plate extending up to interosternite 5/6 (character state 8(1)), including a transverse sternal ridge from interosternite 6/7 to the anterior end of the median plate (character state 12(1); Fig. 12). In contrast, Evans [12], assigned this species to Carcinidae, though with the caveat that the support was not strong. As a personal communication Nathanial Evans offered some doubt about the information content of the 16S rRNA, particularly that Tsang et al. [70] excluded some hypervariable regions because of their focus on Brachyura in general, which nevertheless might have been informative for the relationships of Portunoidea. In conclusion, the placement of *P. orientalis* remains problematic and should be revised taking new genetic data into account.

The only species with this combination of character states not assigned to Portunidae was *Macropipus rugosus* (Carcinidae in both our analyses and that of [12]; see below). We further recovered monophyletic Thalamitinae (represented by *Caphyra loevis*, *Caphyra rotundifrons* and *Lissocarcinus orbicularis*) and monophyletic Carupinae Paulson, 1875 *sensu lato* (represented by *Carupa tenuipes, Catoptrus nitidus* and *Libystes nitidus*) following the nomenclature of Evans [12]. Only the positions of *Callinectes sapidus* and *Portunus inaequalis* differ between our MP and BI analyses (Fig. 33).

In accordance with Evans [12], the Geryonidae of our taxon sampling include *Chaceon mediterraneus* RB Manning & Holthuis, 1989, *Ovalipes ocellatus* and *Raymanninus schmitti*. The relationships between them differ depending on the analysis (Fig. 33). Within Carcinidae, the status of *Carcinus maenas* and *Portumnus latipes* as sister taxa is consistent with previous molecular-based phylogenies [12, 54, 63]. The positioning of the carcinid *Pirimela denticulata* (Montagu, 1808) as more closely related to *Coelocarcinus foliatus* is unexpected, as *P. denticulata* has repeatedly been identified as the sister taxon to the *Carcinus*-*Portumnus* clade [12, 54, 63]. Actually, the phylogenetic placement of *C. foliatus* has repeatedly caused problems due to the unusual external morphology of this species (*“unusual portunid crab”*—[37]; *“certainly not a portunoid”*—[26]), but Evans [12] placed it as a basal carcinid taxon on the basis of molecular data. Interestingly, the identification of *C. foliatus* as the sister taxon to carcinid *Xaiva biguttata* was based herein solely on morphological data, since no genetic data were available for this rare European species. However, *C. foliatus* lacks the P5 anterior coxa muscle originating at interopleurite 7/8 (character states 34(1), 34(3); Fig. 25) which is present in all other taxa of the *Carcinus-Portumnus-Pirimela-Coelocarcinus-Xaiva* clade, and finally, it is the only species of this clade not native to Europe [37]. *Liocarcinus navigator*, *Liocarcinus depurator* and “*Polybius” henslowii* form a monophyletic clade, but with *L. depurator* being more closely related to *“Polybius” henslowii* than to *L. navigator*, which is consistent with earlier findings recognizing the genus *Liocarcinus* to be polyphyletic [12, 40, 54]. In accordance with Evans [12], *Macropipus rugosus* and *Thia scutellata* form a monophyletic clade within Carcinide (Fig. 33). Interestingly for carcinid species, both taxa have an unusual axial skeleton configuration: in *M. rugosus*, the transverse sternal ridge runs from interosternite 6/7 to the anterior end of the median plate (character state 12(1); Fig. 12, otherwise only present in Portunidae), and in *T. scutellata* the median plate extends further than interosternite 4/5 (character state 8(0)), with all interosternites connected to the median plate (character states 13(0), 14(0), 15(0), 16(0); Fig. 11E), a configuration otherwise only present in the heterotrematan outgroup. The latter was represented by a clade comprising all non-portunoid Heterotremata but not *Medorippe lanata* (Fig. 33), which is positioned as sister to a clade comprising Heterotremata and Thoracotremata (not as a heterotrematan taxon as suggested by [25]). However, this and the positon of *Ashtoret lunaris* as the sister taxon to all remaining Heterotremata (Fig. 33; instead of being sister to *Calappa* Weber 1795; [31]) should be taken with caution due to the limited outgroup taxon sampling.

## Discussion of the evolution of the P5-swimming crab morphotype

### Characterising the P5-swimming crab morphotype

**Fig. 34:**
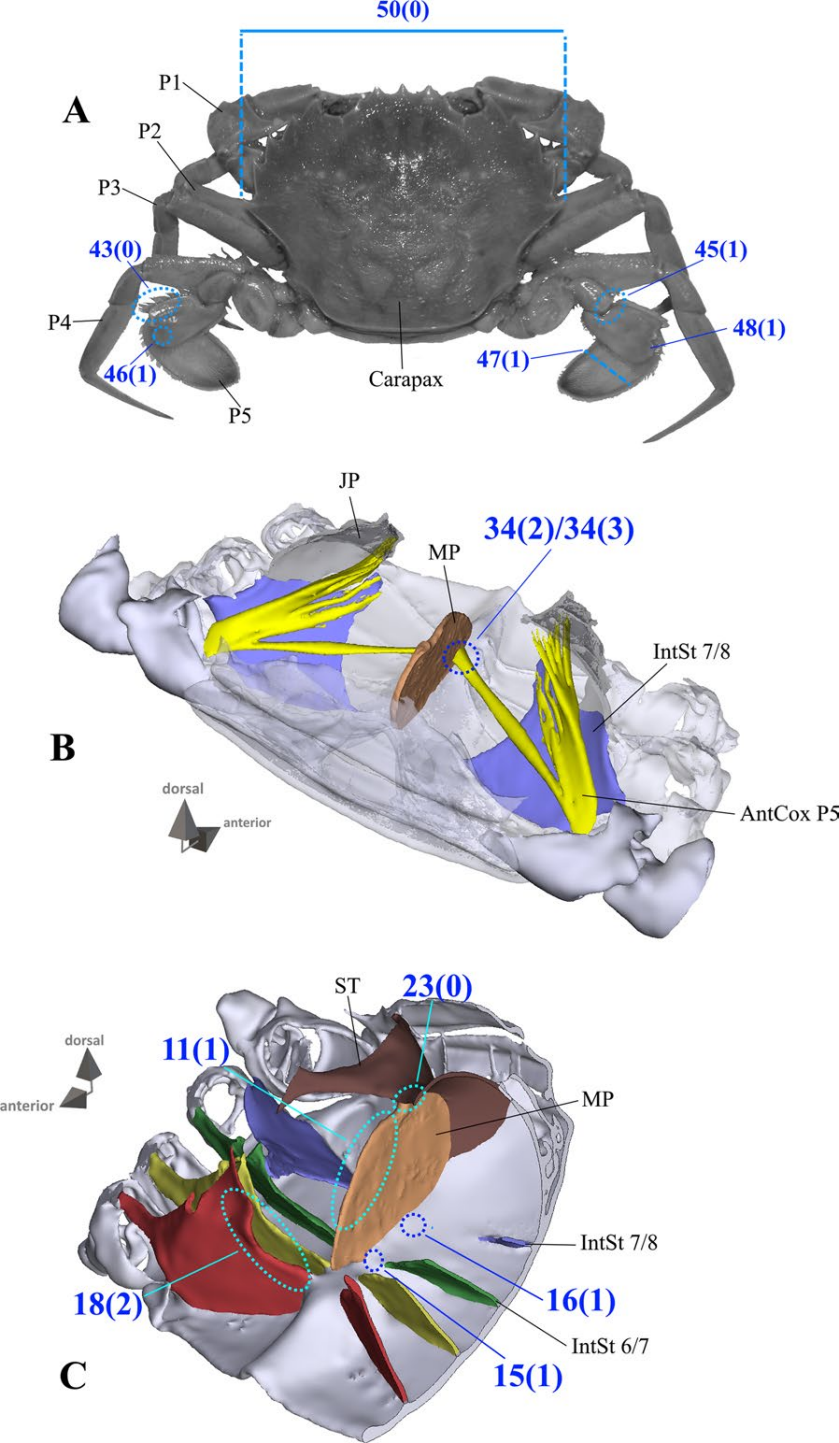
Character states that were found in all examined P5-swimming crabs, exemplified by *Liocarcinus depurator*. **A** Dorsal view of whole body, **B** anterior view of three-dimensional (3D) data representation showing axial skeleton, **C** anterio-lateral view of three-dimensional (3D) data representation showing axial skeleton. *AntCox P5* 5th pereiopod anterior coxa muscle, *IntPl 6/7* interopleurite between thoracomeres 6 and 7, *IntSt* interosternite (with number pair indicating thoracomeres between which it is situated), *MP* median plate

**Fig. 35:**
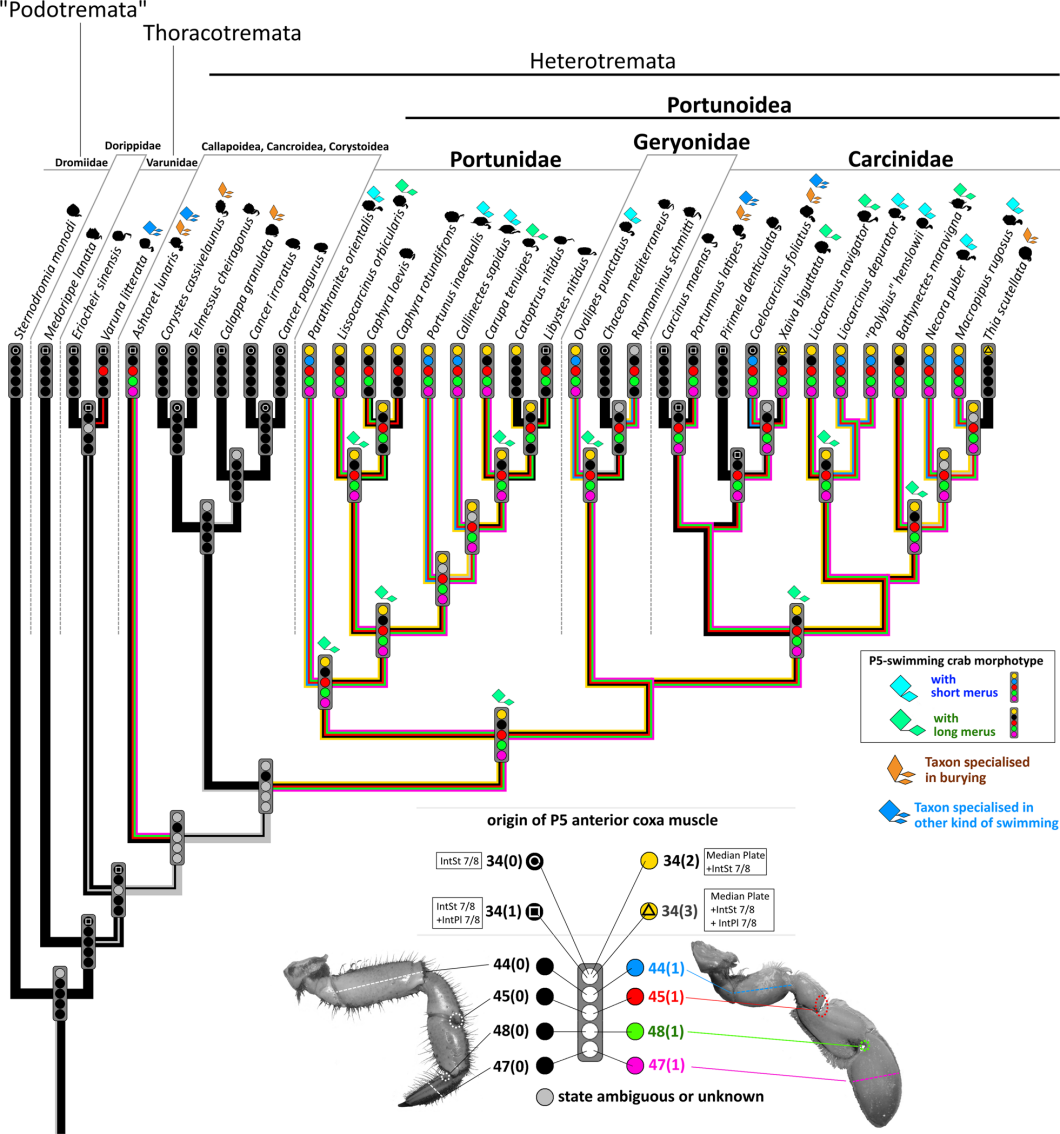
Character states and ancestral state reconstructions of states that unambiguously show whether a taxon is a P5-swimming crab. Character state combinations of each extant species and several ancestral stem species are indicated by different coloured dots, with dot position representing the character and colour (sometimes with symbol) representing the states. Note that symbols above taxa names indicate whether an extant species is assigned to the P5-swimming crab morphotype or considered to be specialised in burying and/or swimming that is different from P5-swimming and that symbols above the nodes of the cladogram indicate whether any reconstructed stem species can be assigned to the P5-swimming crab morphotype

**Fig. 36:**
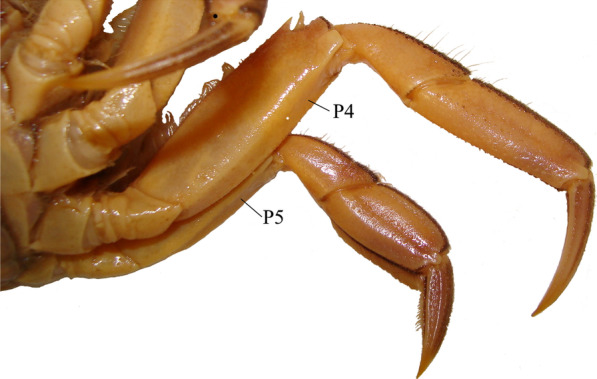
Image showing the 4th pereiopod (P4) and 5th pereiopod (P5) in *Eriocheir japonica* (ventral view)

**Fig. 37:**
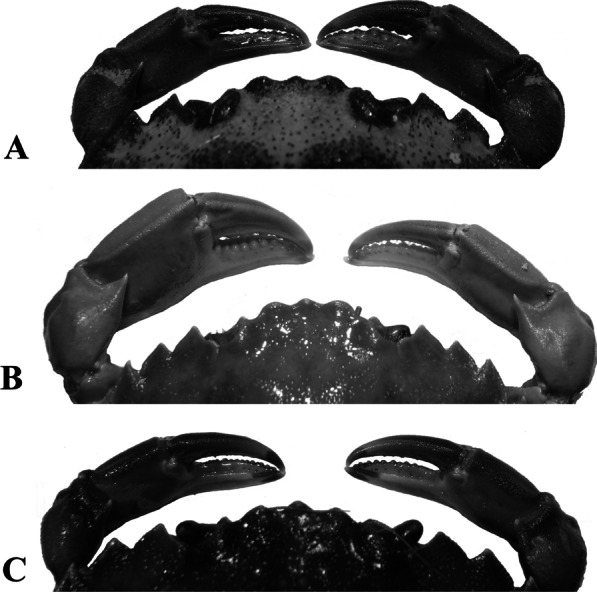
Variability in the degree of heterochely in *Carcinus maenas* (dorsal view). **A** Specimen showing moderate heterochely and heterodonty with right crusher chela being somewhat larger than the left and with molariform denticles, **B** specimen showing pronounced heterochely and heterodonty with left crusher chela being distinctly larger than the right and with molariform denticles, **C** specimen showing homochely and homodonty with no molariform denticles

**Fig. 38:**
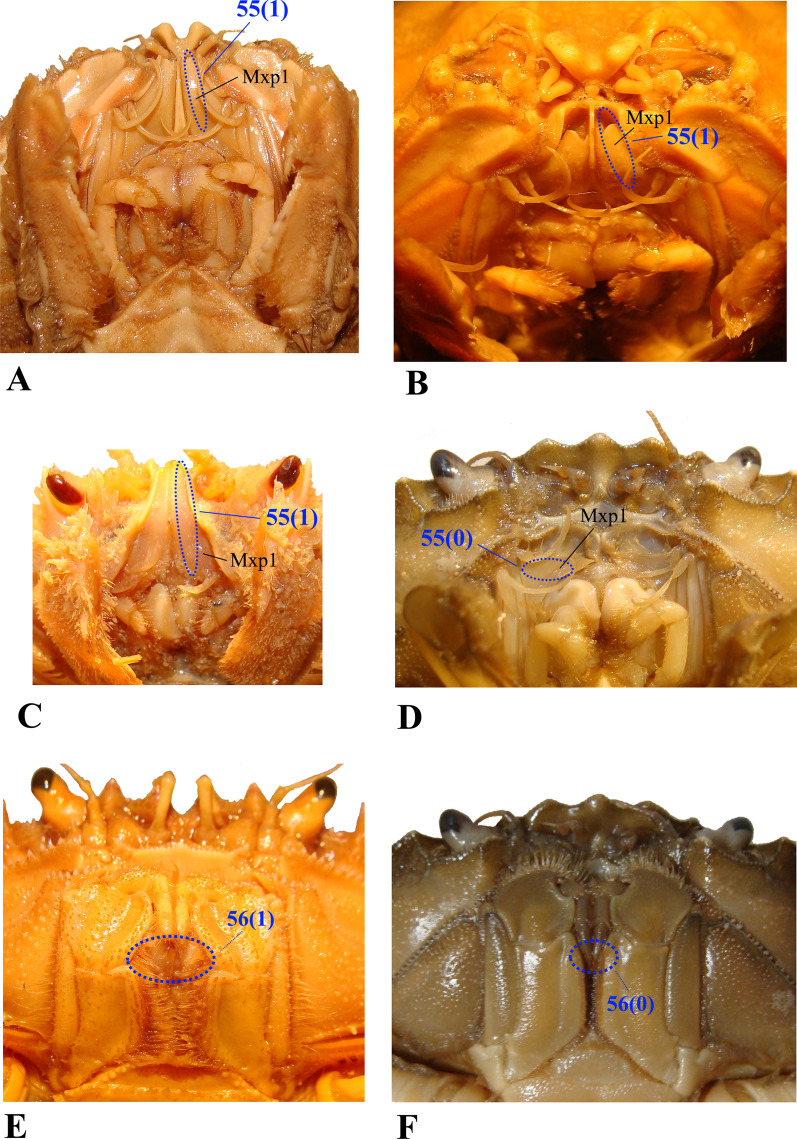
Images indicating character states concerned with degree of closure of exhalant aperture in maxillipede 1 (**A**–**D**) and degree to which maxillipede 3 covers mandibles (**E**, **F**). **A**, **B** *Calappa granulata* seen from anterio-ventral (**A**) and anterio-dorsal (**B**). **C** *Medorippe lanata*, **D** *Carcinus maenas*, **E** *Eriocheir sinensis*, **F** *Carcinus maenas*

**Fig. 39:**
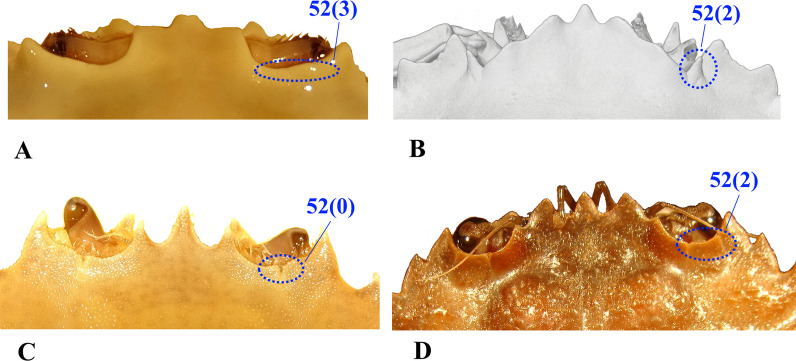
Images indicating character states concerned with surface structure of dorsal orbit margin. **A** *Ashtoret lunaris*, **B** *Xaiva biguttata*, **C** *Ovalipes ocellatus*, **D** *Liocarcinus depurator*

Using our analysis, the P5-swimming crab morphotype characterised by Herter [24], Kühl [29], Schäfer [51] and Hartnoll [21] can now be evaluated by checking it for the character states that all P5-swimmer species in our taxon sample share. Character states occurring in all P5-swimming crabs are summarised in Fig. 34 and Table 2 (the character matrix for the entire taxon sampling is included in the Additional file 1). It is interesting to note that P5-swimmers only occurred in Portunoidea. With respect to how merus length (character 44) should be interpreted, Steudel [66] noted that species displaying the P5-swimming leg with a long merus were generally able to perform the same swimming movements as P5-swimmers with a short merus. This ties in with findings by Schmidt et al. [52], who discovered that theoretical ranges of motion in P5 articulations in *Carupa tenuipes* with a long merus were similar to those in P5-swimming crabs with a short merus. In *Liocarcinus pusillus* (Leach, 1816), a species morphologically similar to *Liocarcinus depurator* but with a longer merus, swimming movements were principally the same as in *L. depurator* (although somewhat less effective; see high-speed recordings of Additional file 34: Video S1, Additional file 35: Video S2, Additional file 36: Video S3 in Supplementary Information). *Liocarcinus navigator*, in which the merus is even longer, also swam in a similar way to typical P5-swimmers, although not as fast or with as much agility (personal observation). In all these species, the P5 anterior coxa muscle originates at the median plate, as is also the case in all typical P5-swimmers with a short merus (character state 34(2)), corroborating the hypothesis by Hazerli and Richter [22] that this is crucial for P5-swimming. Consequently, we do not consider a short merus mandatory in the identification of a swimming leg, although we are well aware that as a result of the variability of merus length there may be high morphological variation in species assigned to the morphotype, presumably resulting in a high level of variation in the effectiveness of P5-swimming ability. In summary, P5-swimming crabs can unambiguously be identified by the character states shown in Fig. 35, with merus length (character 44) representing a state that “merely” influences the effectivity of P5-swimming. The position of the P5 coxa (subdorsal or ventral, see for example [21]) doubtlessly also influences P5-swimming effectivity, but it was not considered here because of the difficulty of conceptualising discrete character states due to high interspecific variability. We further consider *Xaiva biguttata* an (albeit unusual) P5-swimmer since it displays the swimming leg with a long merus (but with no propodus lobe) and its P5 anterior coxa muscle originates at the median plate, though unlike in all other P5-swimmers it additionally originates at interopleurite 7/8 (as in, but not homologously to *Thia*; character state 34(3); Figs. 25C, 35). It should also be mentioned that with merus length, dactylus width (character 47) also varies between species (Figs. 4, 5, 6). In *Raymanninus schmitti* and *Caphyra loevis* we even found species that exhibited all the unambiguous P5-swimmer states mentioned above except for a broad paddle-like dactylus. In *R. schmitti,* however, the origin of the P5 anterior coxa muscle at the median plate needs to be confirmed in other specimens because the musculature was poorly preserved in the only specimen available (Additional file 31).

**Table 2:** Character states present in all P5-swimming crabs

Character	State	Statement
11	1	Sternum, median plate, dorsal margin, shape: more or less convex, without indentations and/or gaps between thoracomeres
15	1	Sternum, interosternite 6/7, connection to median plate: absent
16	1	Sternum, interosternite 7/8, connection to median plate: absent
18	2	Sternum, interosternite 4/5, medial margin, shape: transversal with lower margin being most medial, but not touching interosternite 4/5 of other lateral side
23	0	Sella turcica, covering of dorsal median plate margin: present
34	2 OR 3	Pereiopod 5, anterior coxa muscle, origin: at median plate + interosternite 7/8 OR at median plate + interosternite 7/8 + interopleurite 7/8
43	0	Pereiopod 5, long setae, arrangement: arranged in dense fringes along podomere margins
45	1	Pereiopod 5, carpus, disto-dorsal margin, propodus insertion: present
46	1	Pereiopod 5, propodus, lobe-like expansion of posterio-ventral margin: present
47	1	Pereiopod 5, dactylus, maximum width in relation to maximum width of P5 merus: larger
48	1	Pereiopod 5, dactylus, proximo-ventral margin, shape: concave
50	0	Carapace, maximum width in relation to maximum length: larger or equal

The morphological features characterising the P5-swimming morphotype which Hazerli and Richter [22] identified included muscular features, features of the axial skeleton and also external features of *L. depurator*. However, the greater taxon sampling in the current study revealed that many axial skeleton features in particular mentioned by Hazerli and Richter [22] differ between P5-swimmer taxa. These include the shape and configuration of the interosternite 7/8 process (character 20; morpheme 25 in [22]), the presence and extension of the median plate process (characters 9, 10; morpheme property 18 in [22]), and the distance from the medial edge of interosternite 7/8 to the median plane (character 17; morpheme propertiy 22 in [22]). However, it should be mentioned that all P5-swimmer taxa with a short merus, at least, have the same character states with regard to the latter two characters. Apart from that, it is conspicuous that all unambiguous P5-swimming crab character states shown in Fig. 35 solely refer to features of the P5, including the origination of the P5 anterior coxa muscle at the median plate.

### Ancestral state reconstructions and evolutionary transformations of P5-swimmer character states

Figure 35 shows ancestral state reconstructions and transformations of character states that unambiguously characterise P5-swimmers (raw Mesquite data that show transformations of all morphological character states are supplied in the “Appendix” in Additional file 33). The most important finding is that the P5-swimming morphotype (with a long merus) already evolved in the stem species of Portunoidea (Fig. 35). Starting from this condition, evolutionary transformations proceeded into two general directions. One direction represents evolution into a more efficient P5-swimmer with a short merus. Based on our data, this happened several times independently within Portunoidea (at least twice each in Portunidae and Carcinidae, once in Geryonidae). The second direction is characterised by the loss of P5-swimming crab character states to varying degrees. A complete reversal of the swimming leg into a walking leg occurred independently in *Chaceon mediterraneus*, *Carcinus maenas*, and *Pirimela denticulata* (provided that the position of *P. denticulata* as sister taxon to the clade *Coelocarcinus-Xaiva* is correct; see “Results and discussion of phylogenetic analysis” section), from ancestors that had already “lost” one or several P5-swimming leg states (Fig. 35). *Catoptrus nitidus* evolved a P5 walking leg but retained the origin of the anterior coxa at the median plate and interosternite 7/8 (typical of P5-swimmers; character state 34(2)) from an ancestor that had already “lost” its short merus and broad dactylus (Fig. 35). Generally, within Portunidae, P5-swimmer character states were reversed to varying degrees, but noticeably, in all taxa except for *Libystes nitidus* (which has a significantly smaller median plate), this anterior coxa muscle configuration is retained.

It is interesting to note that a broad paddle-like dactylus (character state 47(1); Fig. 30B) is always associated (= coherent; see for example [48]) with a carpal propodus insertion (character state 45(1); Fig. 30B) and a concave proximo-ventral dactylus margin (character state 48(1); Fig. 30B, F), but not vice versa (Fig. 35). Furthermore, if a paddle-like dactylus (character state 47(1); Fig. 30B) evolved back to a slender dactylus (state 47(0); Fig. 30A) and the carpal propodus insertion was retained (state 45(1); Fig. 30B), a concave proximo-ventral dactylus margin (state 48(1); Fig. 30B, F) is retained, too (which also represents some sort of coherence). This is the case in the common ancestor of both *Caphyra* species, the common ancestor of *C. nitidus* and *L. nitidus*, and in *Raymanninus schmitti* (Fig. 35). *Caphyra rotundifrons*, in which the proximo-ventral margins of the P5 propodus and dactylus form a subchela, is an exception.

**Fig. 40:**
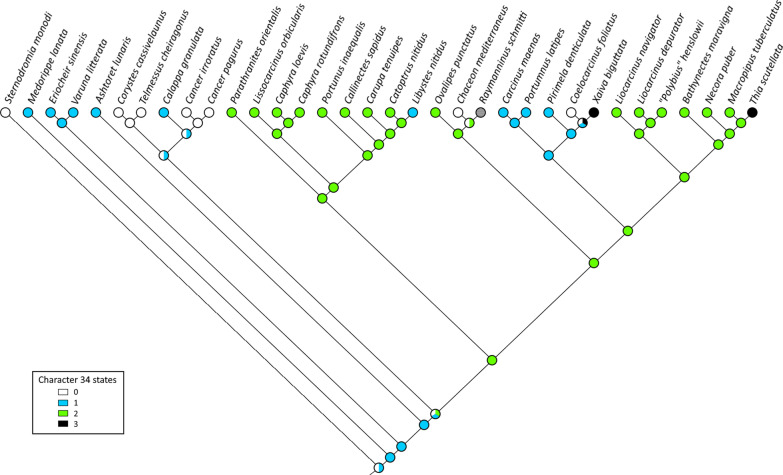
Character states and ancestral state reconstructions of character concerned with origin of the anterior coxa muscle of the 5th pereiopod (character 34)

**Fig. 41:**
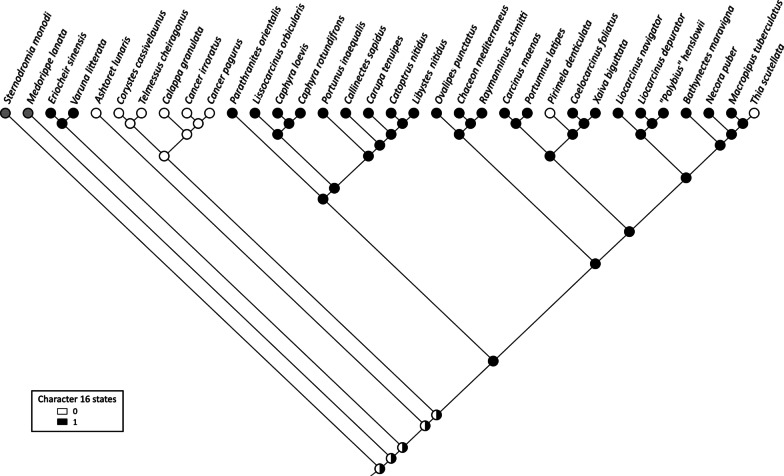
Character states and ancestral state reconstructions of character concerned with the connection between interosternite 7/8 and the median plate (character 16)

The site of origin of the P5 anterior coxa muscle is certainly one of the key characters in the evolution of P5-swimming. However, it is difficult to interpret how this site of origin evolved. Our data show that the conditions typical of a P5-swimmer (origin at median plate and interosternite 7/8; state 34(2); Fig. 25D, E, F) form the plesiomorphic state in Portunoidea (Fig. 35), but it is unclear from which state this evolved (Fig. 40 in “Appendix”). Either it evolved from an origin at interosternite 7/8 only (state 34(0); Fig. 25A) or from an origin at both interosternite 7/8 and interopleurite 7/8 (state 34(1); Figs. 25B, 26). In this case, assuming that an alteration in muscle origin took place during the course of evolution by a gradual shift of one (or several) muscle fibre(s) in its proximal attachment site(s), the connection between interosternite 7/8 and the median plate has to be considered in evolutionary scenarios in order to be able to interpret muscle origin transformations. It is well thinkable that during the course of evolution, one or several of the fibres that originally originated at interosternite 7/8 shifted with their origin to the median plate. However, this step in evolution is only imaginable as long as interosternite 7/8 was connected to the median plate (character state 16(0)). Consequently, even if, based on our ancestral state reconstruction, the state of this character 16 is ambiguous for the ground pattern of Heterotremata (Fig. 41 in “Appendix”), we prefer a scenario in which interosternite 7/8 was connected to the median plate in the heterotrematan stem species (i.e. character state 16(0)). A reversal of the P5 anterior coxa muscle originating at both the median plate and interosternite 7/8 (state 34(2); Fig. 25D, E, F) back to it originating at interosternite 7/8 (state 34(0); Fig. 25A) or at interosternite 7/8 and interopleurite 7/8 (state 34(1); Figs. 25B, 26) in taxa in which a connection between interosternite 7/8 and the median plate was absent is imaginable if a degeneration of the muscle fibres attached to the median plate is assumed. This must have happened in the stem species of the *Carcinus-Portumnus-Pirimela-Coelocarcinus-Xaiva* clade (Fig. 35). If this was the case, *Xaiva biguttata* regained a P5 anterior coxa muscle origin at the median plate, but its phylogenetic positon should be treated with caution as it is only based on morphological data.

Until now, few hypotheses have been put forward on the evolution of P5-swimming crabs. Hartnoll [21] and Steudel [66] were the first to formulate explicit hypotheses, but not on the basis of phylogenetic relationships. Hartnoll [21] suggested a transformation series to swimming crabs largely on the basis of one single morphological feature, the dactylus width of pereiopods 2–5. As far as we understand ([21] was rather imprecise in his statements), *Carcinus* was considered a “basal” genus, with its P5 podomeres which are just slightly broader than those of P4 being interpreted as a plesiomorphic character state. However, in accordance with previous phylogenies, our combined analysis identified *Carcinus maenas* as a highly derived taxon within Portunoidea (Figs. 33, 35; [12, 26, 54]). We consider the P5 podomere broadening in *C. maenas* not to be homologous to that in P5-swimmers and other taxa with a paddle-like dactylus (see also “Characters concerned with the external morphology of pereiopod 5” section). *“Polybius” henslowii*, in which Hartnoll [21] considered pereiopods 2–4 to be modified for swimming, was considered a more advanced P5-swimmer than those in which only the P5 was modified. It was assumed that *“Polybius” henslowii* evolved from an ancestor in which only the P5 was modified, which is well possible considering our data (Fig. 35).

**Fig. 42:**
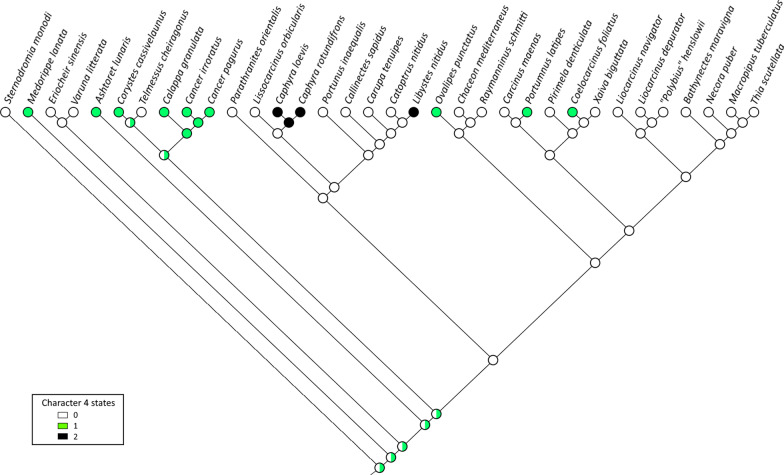
Character states and ancestral state reconstructions of character concerned with sternum shape (character 4)

Steudel [66] distinguished between several swimming crab morphotypes (or *“Konstruktionstypen”* as they were termed) denoted after distinct genera, namely the *Liocarcinus-*type, *Polybius-*type, *Ovalipes-*type and *Portumnus-*type, but she recognised that only the former three types were capable of the typical P5-swimming movements. Steudel [66] also suggested that the *Ovalipes-*type evolved from the *Portumnus-*type, independently of the *Liocarcinus-* and *Polybius-*types, an assumption based mainly on the shape of the sternum (see character state 4(1) herein; Fig. 8B, E). In contrast, our data show that the similar sternum shapes in *Ovalipes ocellatus* and *Portumnus latipes* evolved independently of each other (Fig. 42 in “Appendix”). Steudel [66] further suggested that the *Liocarcinus-* and *Polybius-*types both independently evolved from a non-swimming morphotype adapted to effective underwater running (our translation, Steudel used the term *“Unterwasserrenner”*) with a straight, broad sternum (character states 3(1) and 4(0) herein) and well-developed walking legs. Subsequent evolution into the *Liocarcinus-* and/or *Polybius-*type was simply interpreted as a further “optimization” of underwater running. However, since our analysis recovered the underwater runners *Carcinus maenas* and *Chaceon mediterraneus* as derived species within Portunoidea that lost P5-swimmer character states secondarily (Figs. 33, 35), Steudel’s assumption is herewith refuted. Spiridonov et al. [63] also briefly discussed the evolution of the P5-swimmer morphotype, taking into consideration the statements in Steudel [66] and a phylogeny deduced from molecular data but no morphological characters. In contrast to our findings, Spiridonov et al. [63] concluded that the P5-swimmer morphotype most probably evolved three times independently in Carcinidae, Geryonidae and Portunidae, respectively.

Spiridonov [64] speculated that a broad paddle-like dactylus adapted to burying represented a preadaptation to P5-swimming. Several of the species we examined are generally considered to be taxa specialised in a burying mode of life, namely *Ashtoret lunaris*, *Corystes cassivelaunus, Calappa granulata, Portumnus latipes* and *Thia scutellata* [13, 14, 46, 51, 71], and perhaps also *Coelocarcinus foliatus* [37]*.* However, of these species, only *A. lunaris, P. latipes* and *C. foliatus* have a paddle-like P5 dactylus (character state 47(1)). *A. lunaris* probably evolved this state independently of Portunoidea, and *P. latipes* and *C. foliatus* are derived portunoids whose specialisation in burying represents an apomorphy which evolved after P5-swimming (Fig. 35). In the light of these data, Spiridonov [64]’s suggestion is rejected here. As already mentioned, *Varuna litterata, A. lunaris*, and also probably the portunoid *P. latipes* (perhaps also *C. foliatus*) are known to be effective swimmers, exhibiting morphological features which facilitate swimming similar to those in P5-swimmers (like the paddle-like dactyli of pereiopods), but also some which are quite different from those of P5-swimming crabs (like the P5 anterior coxa muscle not originating at the median plate). P5-typical swimming movements above the carapace can thus not be performed by these taxa, as was shown by Schmidt et al. ([52]; in *C. foliatus*, however, this still has to be tested).

With regard to axial skeleton features, an interesting aspect of P5-swimming crab evolution is the “brachyuran sella turcica” (sensu [19]), a sella turcica connected to interosternite 7/8. However, in the species examined herein, this connection was only present in the outgroup taxa *Sternodromia monodi, Medorippe lanata*, *Eriocheir sinensis* and *V. litterata* (character state 19(0); Fig. 14A, B, D, E). In all other taxa, interosternite 7/8 is without a direct connection to the sella turcica (character state 19(1); Figs. 14G, H, 15A–D), instead possessing an interosternal process which sometimes touches interosternite 6/7. This suggests that in the ground pattern of Heterotremata, the direct connection between interosternite 7/8 and the sella turcica (the “brachyuran sella turcica”) was secondarily lost. Interestingly, in the taxa examined, the absence of a connection between interosternite 7/8 and the sella turcica was always associated with the presence of a junction plate cavity, which offers space and attachment sites for the large extrinsic P5 musculature of P5-swimmers. We thus interpret this as a preadaptation for the evolution into a P5-swimming crab, and as an explanation of why the P5-swimming crab morphotype evolved in Heterotremata only.

## Conclusions

We demonstrate that a detailed morphological examination on the basis of accurate character conceptualisation can be used in combination with genetic data (as total evidence analysis) not only to formulate a robust phylogenetic hypothesis, but also to reconstruct ancestral morphologies and evolutionary transformations. A careful selection of terminal taxa that display the morphological disparity within this group is a prerequisite for convincing statements. Our data suggest that the stem species of Portunoidea already showed the typical P5-swimming crab morphotype, but with a merus that was not as short as in the highly effective P5-swimmer morphotype that is represented by several extant species. The axial skeleton and extrinsic musculature configuration in different P5-swimming crab species can be highly diverse, although all have in common that the extrinsic anterior coxa muscle originates at the median plate. A species which shows this character state in combination with certain external states of the swimming leg (P5) can unambiguously be identified as a P5-swimming crab. The lack of a connection between interosternite 7/8 and the sella turcica is identified as an autapomorphy of Heterotremata and a possible preadaptation to the P5-swimmer morphotype. Earlier hypotheses on the evolution of the morphotype, namely that a paddle-like dactylus in Portunoidea evolved as preadaptation for burying, and that the broader P5 podomeres relative to the P4 podomeres in *Carcinus maenas* (which is well-known for its lack of P5-swimmer features) were homologuous to the broader P5 podomeres in P5-swimming crabs are rejected on the basis of morphological and phylogenetic data. The phylogenetic positions of *Coelocarcinus foliatus, Parathranites orientalis* and *Xaiva biguttata* remain uncertain and need revision.

### Supplementary Information

**Additional file 1.** Character state data matrix showing character states of all characters in the species examined.

**Additional file 2.** Three-dimensional (3D) model of *Sternodromia monodi* showing the axial skeleton, proximal podomeres of thoracomeres 4–8 and P5 extrinsic musculature. Use model hierarchy to show extrinsic musculature.

**Additional file 3.** Three-dimensional (3D) model of *Eriocheir sinensis* showing the axial skeleton, proximal podomeres of thoracomeres 4–8 and P5 extrinsic musculature. Use model hierarchy to show extrinsic musculature.

**Additional file 4.** Three-dimensional (3D) model of *Varuna litterata* showing the axial skeleton, proximal podomeres of thoracomeres 4–8 and P5 extrinsic musculature. Use model hierarchy to show extrinsic musculature.

**Additional file 5.** Three-dimensional (3D) model of *Calappa granulata* showing the axial skeleton, proximal podomeres of thoracomeres 4–8 and P5 extrinsic musculature. Use model hierarchy to show extrinsic musculature.

**Additional file 6.** Three-dimensional (3D) model of *Ashtoret lunaris* showing the axial skeleton, proximal podomeres of thoracomeres 4–8, some P2–P4 extrinsic muscles and P5 extrinsic musculature. Use model hierarchy to show extrinsic musculature.

**Additional file 7.** Three-dimensional (3D) model of *Cancer irroratus* showing the axial skeleton, proximal podomeres of thoracomeres 4–8 and P5 extrinsic musculature. Use model hierarchy to show extrinsic musculature.

**Additional file 8.** Three-dimensional (3D) model of *Cancer pagurus* showing the axial skeleton, proximal podomeres of thoracomeres 4–8, P5 intrinsic basi-ischium muscles and P5 extrinsic musculature. Use model hierarchy to show musculature.

**Additional file 9.** Three-dimensional (3D) model of *Corystes cassivelaunus* showing the axial skeleton and proximal podomeres of thoracomeres 4–8.

**Additional file 10.** Three-dimensional (3D) model of *Telmessus cheiragonus* showing the axial skeleton, proximal podomeres of thoracomeres 4–8 and P5 extrinsic musculature. Use model hierarchy to show extrinsic musculature.

**Additional file 11.** Three-dimensional (3D) model of *Medorippe lanata* showing the axial skeleton, proximal podomeres of thoracomeres 4–8, some P2 and P4 extrinsic muscles and P5 extrinsic musculature.

**Additional file 12.** Three-dimensional (3D) model of *Bathynectes maravigna* showing the axial skeleton, proximal podomeres of thoracomeres 4–8 and P5 extrinsic musculature. Use model hierarchy to show extrinsic musculature.

**Additional file 13.** Three-dimensional (3D) model of *Carcinus maenas* showing the axial skeleton, proximal podomeres of thoracomeres 4–8, P5 intrinsic basi-ischium muscles and P2–P5 extrinsic musculature.

**Additional file 14.** Three-dimensional (3D) model of *Coelocarcinus foliatus* showing the axial skeleton, proximal podomeres of thoracomeres 4–8, P4 extrinsic basi-ischium muscles and P5 extrinsic musculature. Use model hierarchy to show extrinsic musculature.

**Additional file 15.** Three-dimensional (3D) model of *Liocarcinus depurator* showing the axial skeleton, proximal podomeres of thoracomeres 4–8 and P2–P5 extrinsic musculature.

**Additional file 16.** Three-dimensional (3D) model of *Pirimela denticulata* showing the axial skeleton, proximal podomeres of thoracomeres 4–8 and P5 extrinsic musculature. Use model hierarchy to show extrinsic musculature.

**Additional file 17.** Three-dimensional (3D) model of “*Polybius*” *henslowii* showing the axial skeleton, proximal podomeres of thoracomeres 4–8, P5 intrinsic basi-ischium muscles and P5 extrinsic musculature. Use model hierarchy to show musculature.

**Additional file 18.** Three-dimensional (3D) model of *Portumnus latipes* showing the axial skeleton, proximal podomeres of thoracomeres 4–8 and P2–P5 extrinsic musculature. Use model hierarchy to show extrinsic musculature.

**Additional file 19.** Three-dimensional (3D) model of *Thia scutellata* showing the axial skeleton, proximal podomeres of thoracomeres 4–8, P5 intrinsic basi-ischium muscles and P2–P5 extrinsic musculature. Use model hierarchy to show musculature.

**Additional file 20.** Three-dimensional (3D) model of *Xaiva biguttata* showing the axial skeleton, proximal podomeres of thoracomeres 4–8, P5 intrinsic basi-ischium muscles and P5 extrinsic musculature. Use model hierarchy to show musculature.

**Additional file 21.** Three-dimensional (3D) model of *Chaceon mediterraneus* showing the axial skeleton, proximal podomeres of thoracomeres 4–8 and P5 extrinsic musculature. Use model hierarchy to show extrinsic musculature.

**Additional file 22.** Three-dimensional (3D) model of *Ovalipes ocellatus* showing the axial skeleton, proximal podomeres of thoracomeres 4–8, some P2–P4 extrinsic muscles and P5 extrinsic musculature. Use model hierarchy to show extrinsic musculature.

**Additional file 23.** Three-dimensional (3D) model of *Caphyra rotundifrons* showing the axial skeleton, proximal podomeres of thoracomeres 4–8, P5 intrinsic basi-ischium muscles and P5 extrinsic musculature. Use model hierarchy to show musculature.

**Additional file 24.** Three-dimensional (3D) model of *Carupa tenuipes* showing the axial skeleton, proximal podomeres of thoracomeres 4–8 and P5 extrinsic musculature. Use model hierarchy to show extrinsic musculature.

**Additional file 25.** Three-dimensional (3D) model of *Catoptrus nitidus* showing the axial skeleton, proximal podomeres of thoracomeres 4–8 and P5 extrinsic musculature. Use model hierarchy to show extrinsic musculature.

**Additional file 26.** Three-dimensional (3D) model of *Libystes nitidus* showing the axial skeleton, proximal podomeres of thoracomeres 4–8, some P2–P4 extrinsic muscles, some P5 intrinsic muscles and P5 extrinsic musculature. Use model hierarchy to show musculature.

**Additional file 27.** Three-dimensional (3D) model of *Portunus inaequalis* showing the axial skeleton, proximal podomeres of thoracomeres 4–8 and P5 extrinsic musculature. Use model hierarchy to show extrinsic musculature.

**Additional file 28.** Low-resolution three-dimensional (3D) model of *Medorippe lanata* showing the axial skeleton and proximal podomeres of thoracomeres 4–8.

**Additional file 29.** Low-resolution three-dimensional (3D) model of “*Polybius*” *henslowii* showing the axial skeleton and proximal podomeres of thoracomeres 4–8.

**Additional file 30.** Low-resolution three-dimensional (3D) model of *Thia scutellata* showing the axial skeleton and proximal podomeres of thoracomeres 4–8.

**Additional file 31.** Low-resolution three-dimensional (3D) model of *Ovalipes ocellatus* showing the axial skeleton and proximal podomeres of thoracomeres 4–8.

**Additional file 32.** Low-resolution three-dimensional (3D) model of *Libystes nitidus* showing the axial skeleton and proximal podomeres of thoracomeres 4–8.

**Additional file 33.** Raw Mesquite data that show morphological character states in the species examined and those parsimonily reconstructed in their ancestors.

**Additional file 34: Video S1.** High-speed camera recordings showing swimming behaviour in *Liocarcinus depurator* (1000 fps). Recordings conducted with REDLAKE MotionXtra HG-100 K (using the software MotionCentral v2.7.5 from Redlake MASD, LLC) in combination with a Nikon objective AF-S VR Micro-Nikkor 105 mm f/2l8G IF-ED and various Polaroid 62 mm macro-lenses (+1, +2, +4 and +10 diopter filters)).

**Additional file 35: Video S2.** High-speed camera recordings showing swimming behaviour in *Liocarcinus depurator* (1000 fps). Recordings conducted with REDLAKE MotionXtra HG-100 K (using the software MotionCentral v2.7.5 from Redlake MASD, LLC) in combination with a Nikon objective AF-S VR Micro-Nikkor 105 mm f/2l8G IF-ED and various Polaroid 62 mm macro-lenses (+1, +2, +4 and +10 diopter filters)).

**Additional file 36: Video S3.** High-speed camera recordings showing swimming behaviour in *Liocarcinus pusillus* (1000 fps). Recordings conducted with REDLAKE MotionXtra HG-100 K (using the software MotionCentral v2.7.5 from Redlake MASD, LLC) in combination with a Nikon objective AF-S VR Micro-Nikkor 105 mm f/2l8G IF-ED and various Polaroid 62 mm macro-lenses (+1, +2, +4 and +10 diopter filters)).

## Data Availability

Data generated or analysed during this study that are not included in this published article (and its “Appendix”) are available from the corresponding author on reasonable request.

## Appendix

See Figs. 36, 37, 38, 39, 40, 41 and 42.
