# Supplementary figures and images for: New insights into the evolution of portunoid swimming crabs (Portunoidea, Heterotremata, Brachyura) and the brachyuran axial skeleton
Source: Front Zool. 2022 Oct 27;19:24. doi: 10.1186/s12983-022-00467-8 (PMC9609296; doi:10.1186/s12983-022-00467-8)

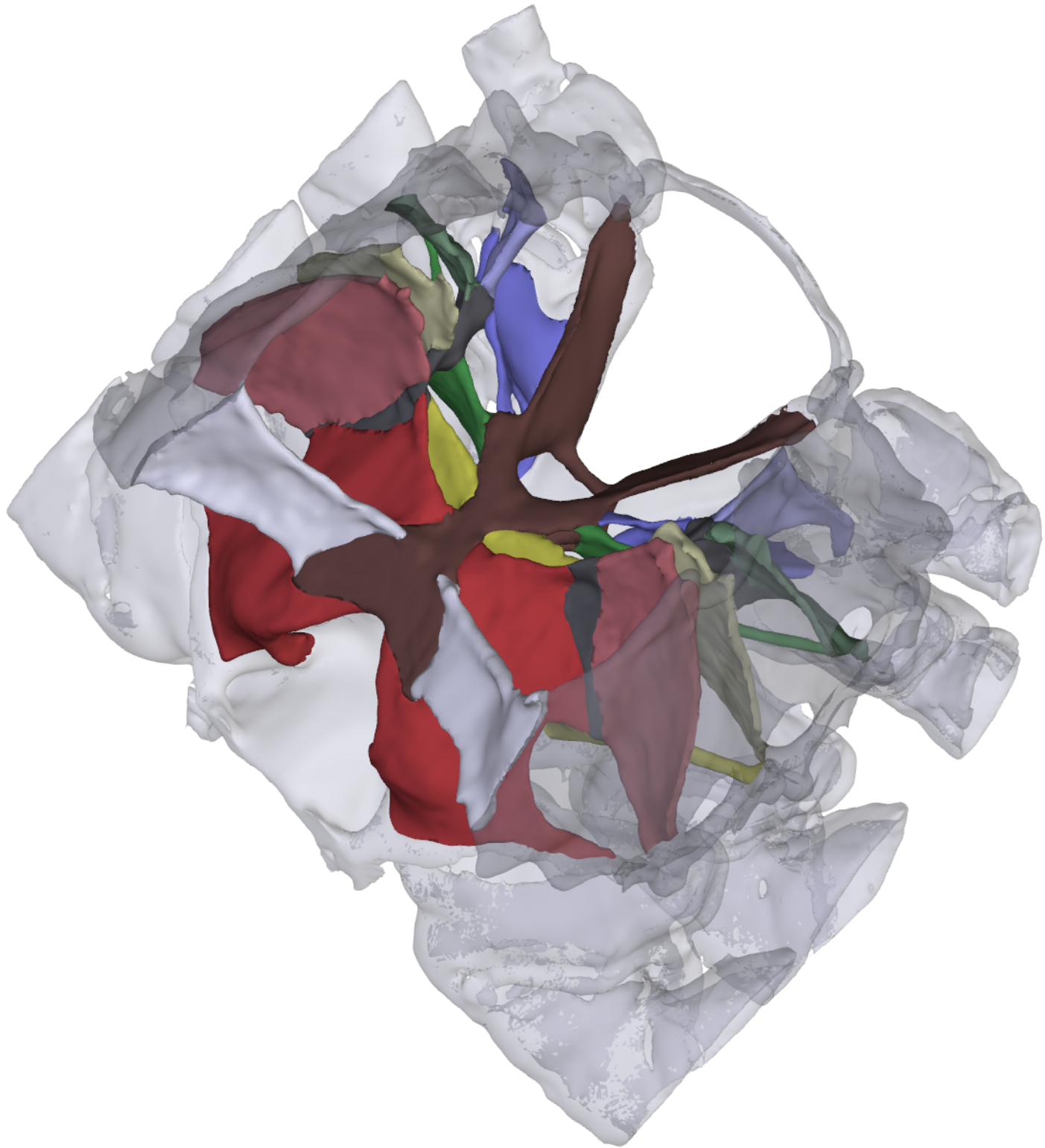

Supplement: Supplementary file 2 — Additional file 2. Three-dimensional (3D) model of Sternodromia monodi showing the axial skeleton, proximal podomeres of thoracomeres 4–8 and P5 extrinsic musculature. Use model hierarchy to show extrinsic musculature. [file 12983_2022_467_MOESM2_ESM.pdf]

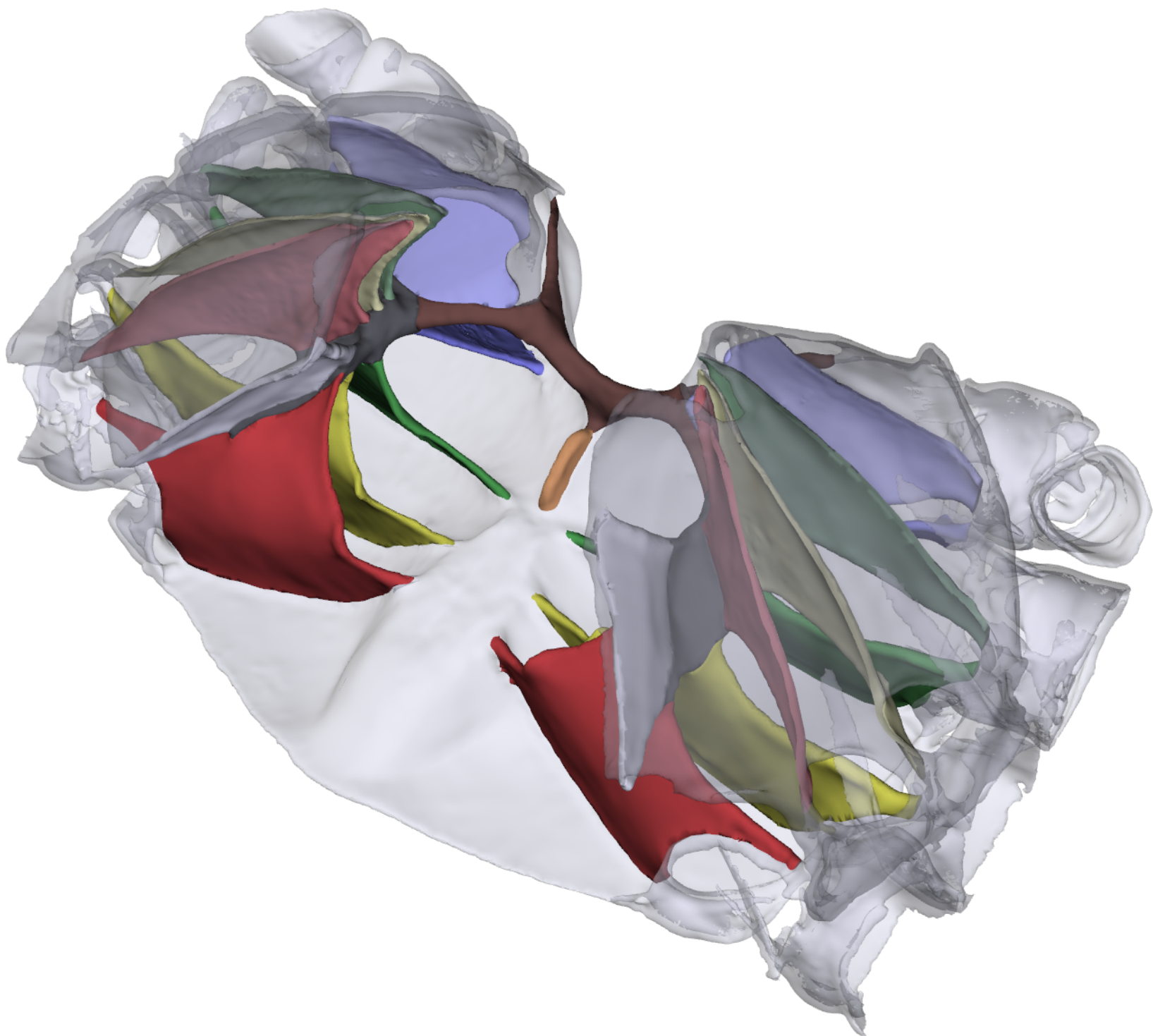

Supplement: Supplementary file 3 — Additional file 3. Three-dimensional (3D) model of Eriocheir sinensis showing the axial skeleton, proximal podomeres of thoracomeres 4–8 and P5 extrinsic musculature. Use model hierarchy to show extrinsic musculature. [file 12983_2022_467_MOESM3_ESM.pdf]

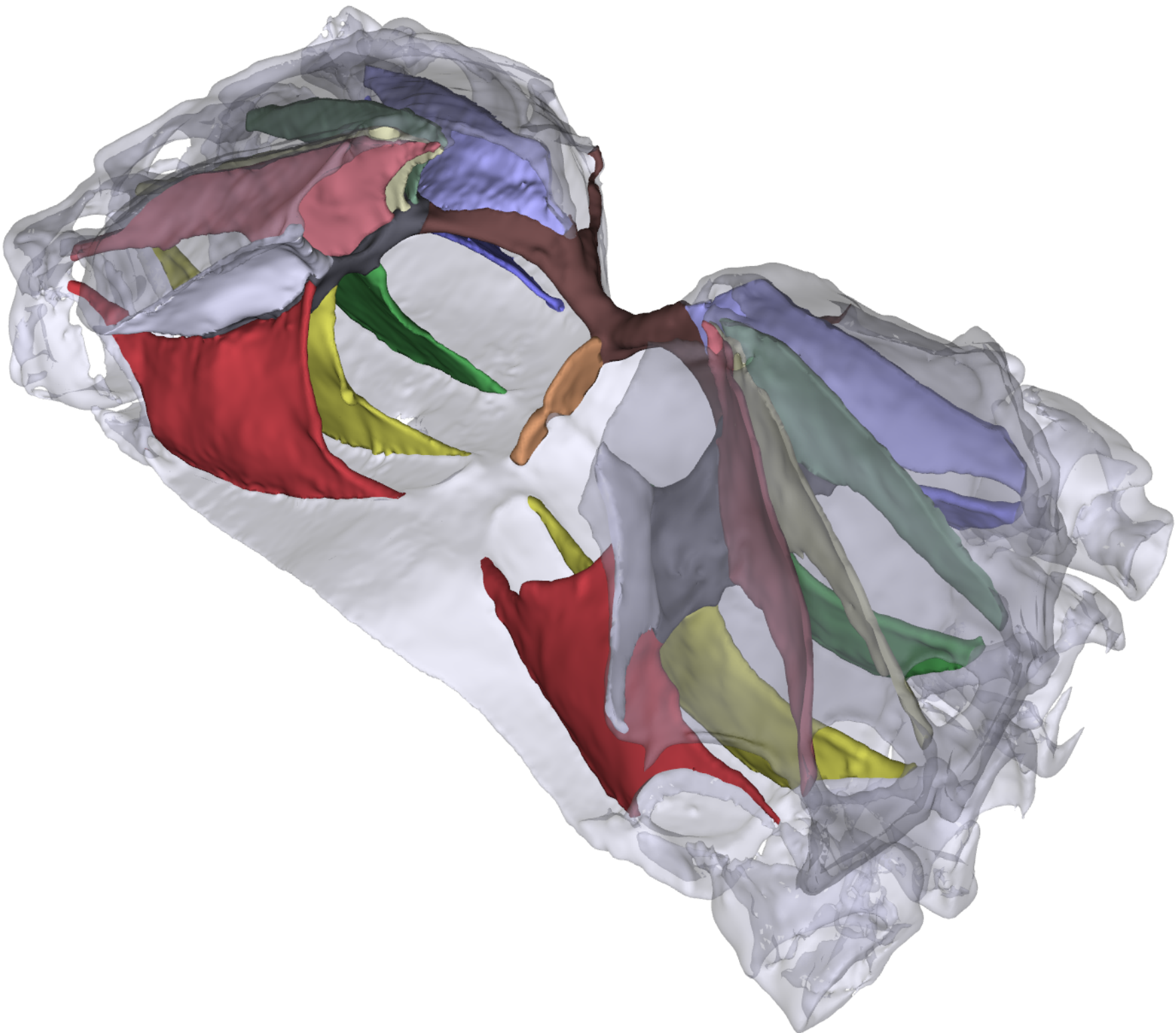

Supplement: Supplementary file 4 — Additional file 4. Three-dimensional (3D) model of Varuna litterata showing the axial skeleton, proximal podomeres of thoracomeres 4–8 and P5 extrinsic musculature. Use model hierarchy to show extrinsic musculature. [file 12983_2022_467_MOESM4_ESM.pdf]

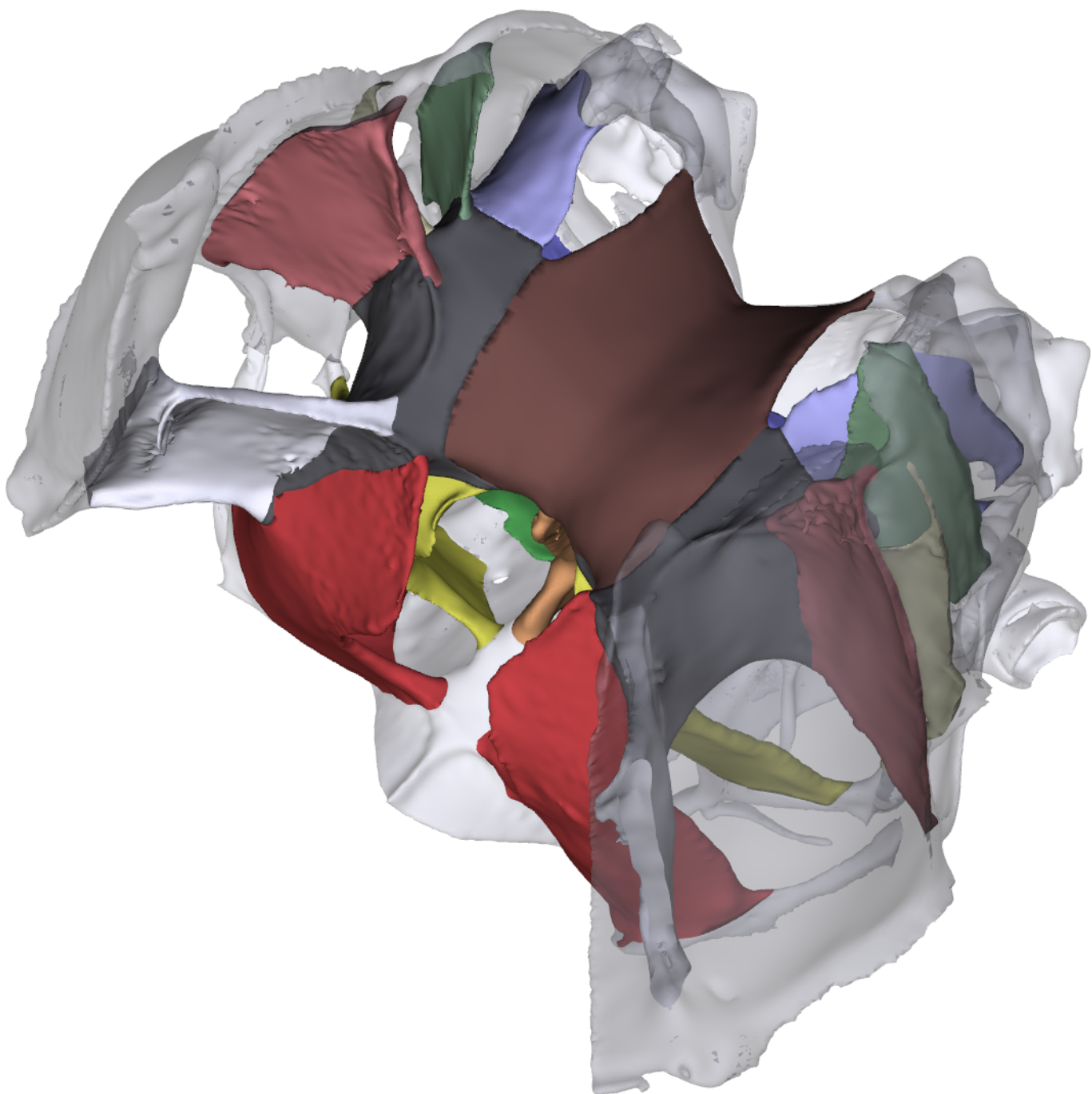

Supplement: Supplementary file 5 — Additional file 5. Three-dimensional (3D) model of Calappa granulata showing the axial skeleton, proximal podomeres of thoracomeres 4–8 and P5 extrinsic musculature. Use model hierarchy to show extrinsic musculature. [file 12983_2022_467_MOESM5_ESM.pdf]

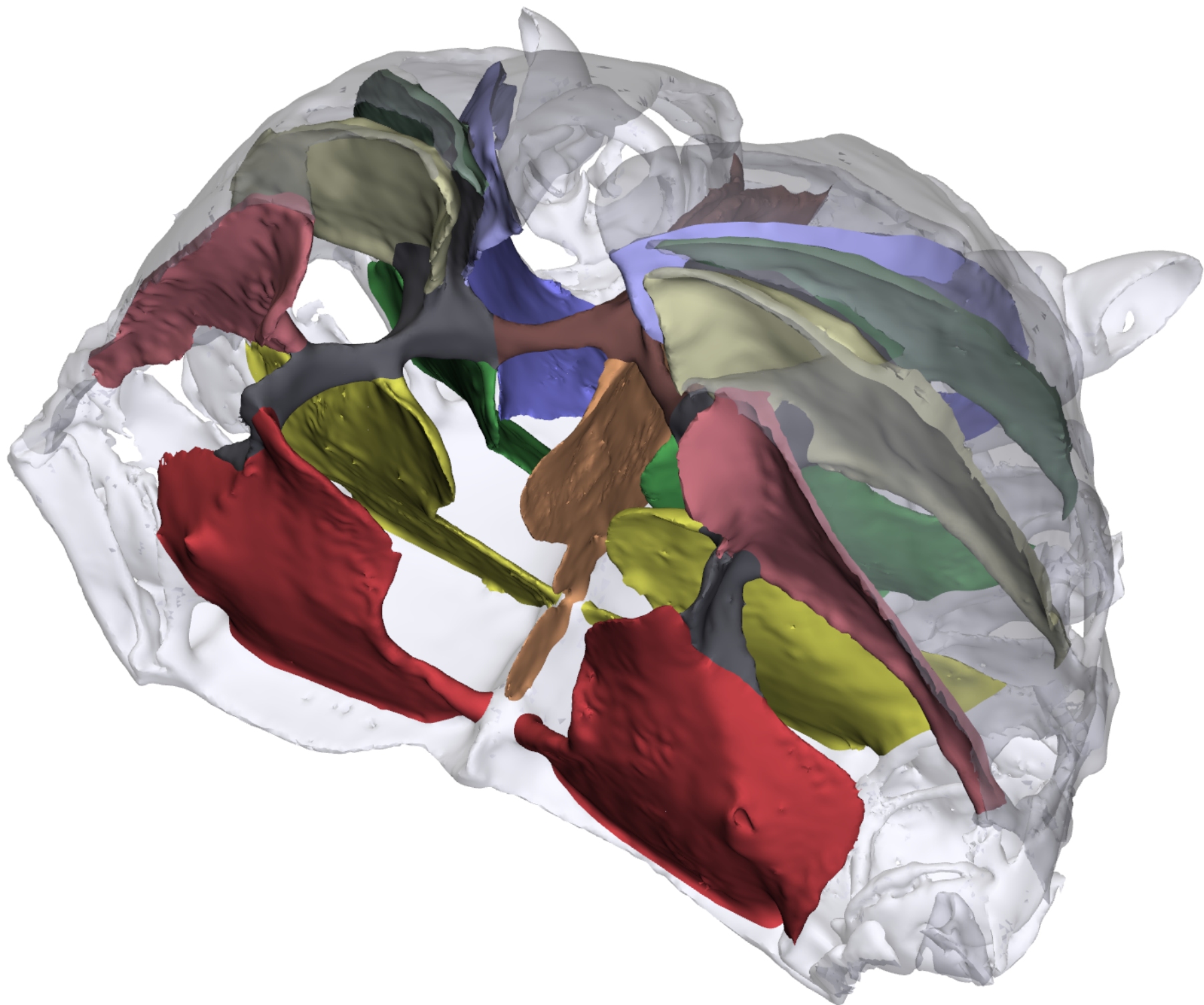

Supplement: Supplementary file 6 — Additional file 6. Three-dimensional (3D) model of Ashtoret lunaris showing the axial skeleton, proximal podomeres of thoracomeres 4–8, some P2–P4 extrinsic muscles and P5 extrinsic musculature. Use model hierarchy to show extrinsic musculature. [file 12983_2022_467_MOESM6_ESM.pdf]

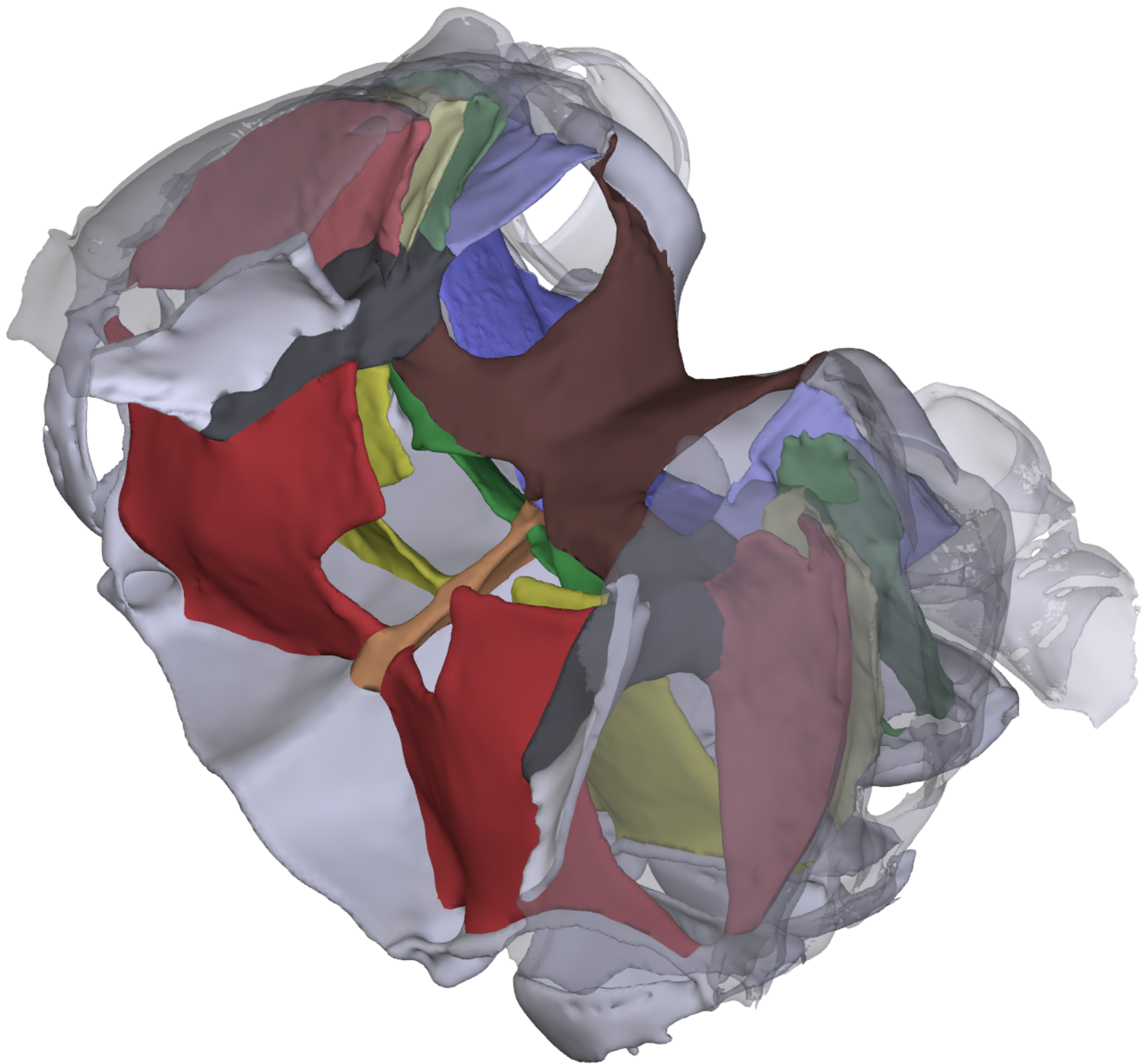

Supplement: Supplementary file 7 — Additional file 7. Three-dimensional (3D) model of Cancer irroratus showing the axial skeleton, proximal podomeres of thoracomeres 4–8 and P5 extrinsic musculature. Use model hierarchy to show extrinsic musculature. [file 12983_2022_467_MOESM7_ESM.pdf]

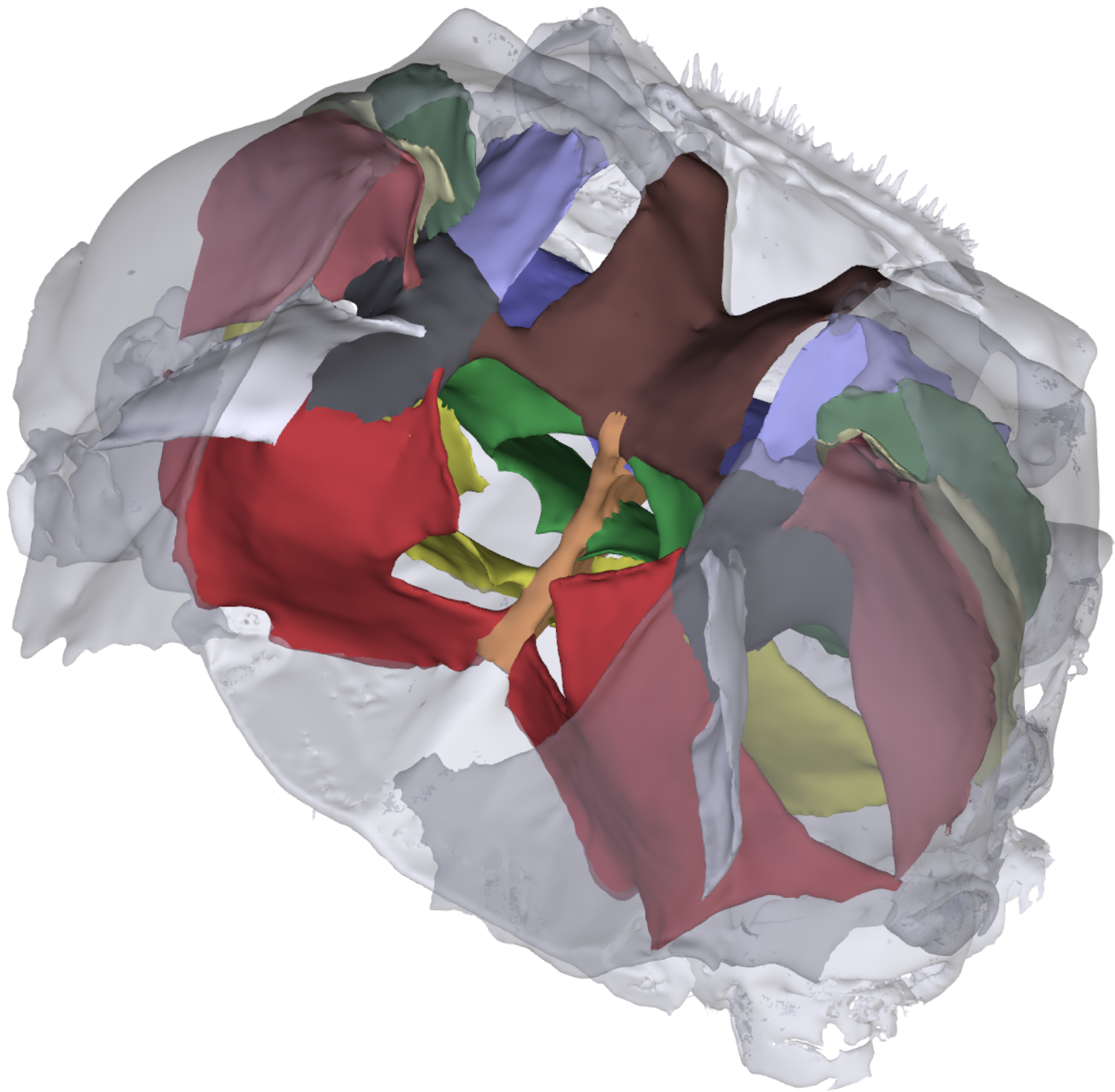

Supplement: Supplementary file 8 — Additional file 8. Three-dimensional (3D) model of Cancer pagurus showing the axial skeleton, proximal podomeres of thoracomeres 4–8, P5 intrinsic basi-ischium muscles and P5 extrinsic musculature. Use model hierarchy to show musculature. [file 12983_2022_467_MOESM8_ESM.pdf]

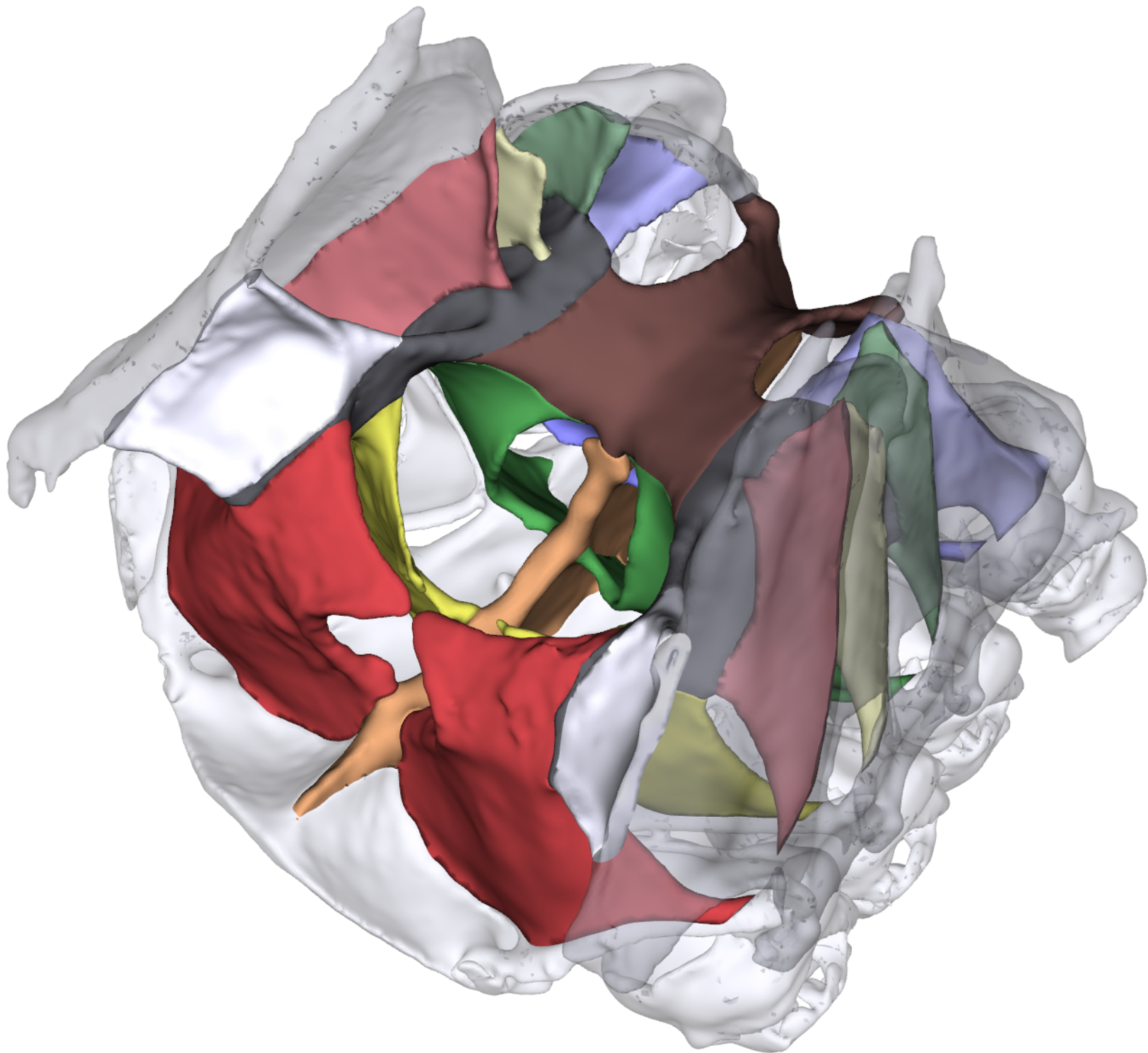

Supplement: Supplementary file 9 — Additional file 9. Three-dimensional (3D) model of Corystes cassivelaunus showing the axial skeleton and proximal podomeres of thoracomeres 4–8. [file 12983_2022_467_MOESM9_ESM.pdf]

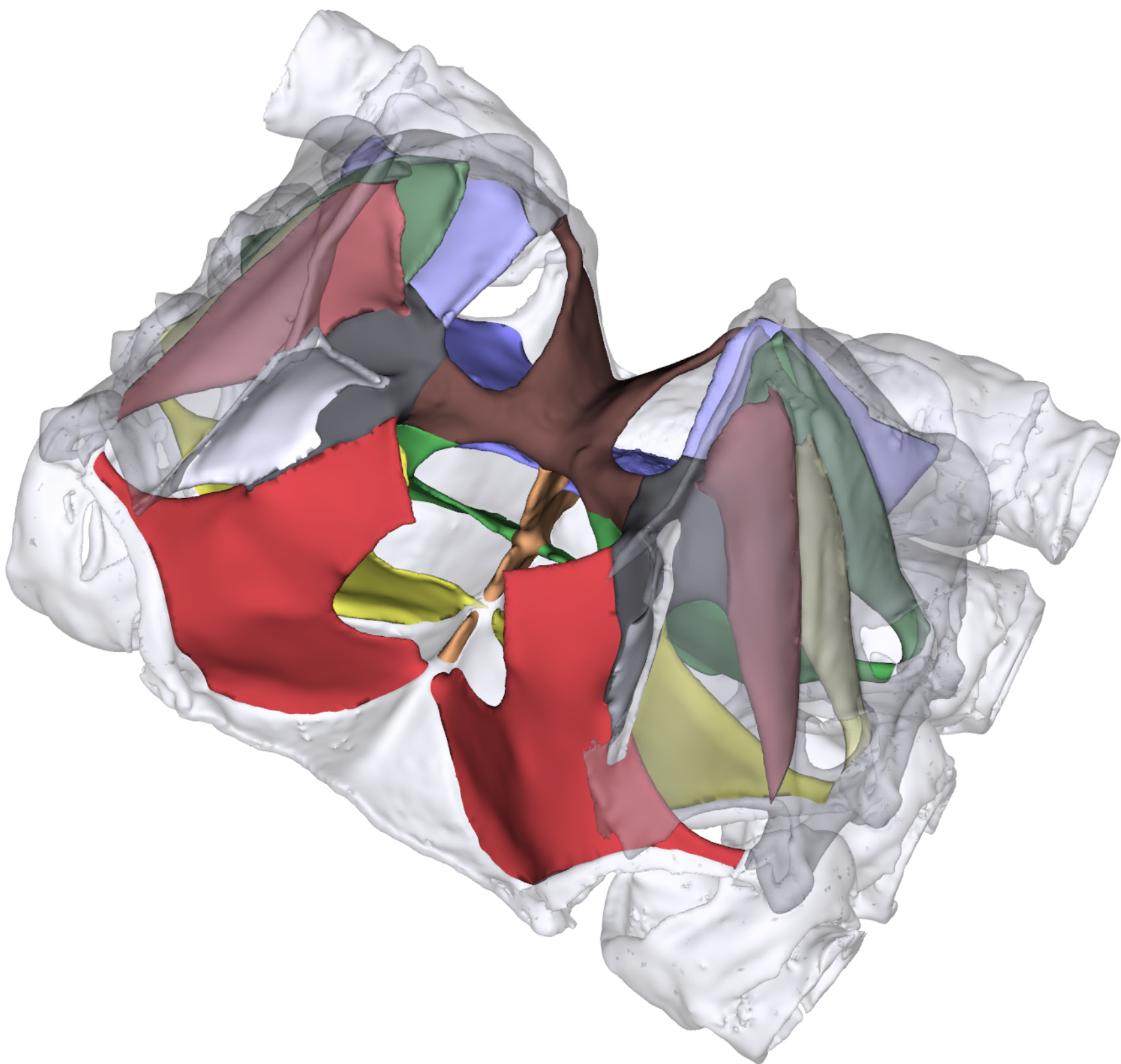

Supplement: Supplementary file 10 — Additional file 10. Three-dimensional (3D) model of Telmessus cheiragonus showing the axial skeleton, proximal podomeres of thoracomeres 4–8 and P5 extrinsic musculature. Use model hierarchy to show extrinsic musculature. [file 12983_2022_467_MOESM10_ESM.pdf]

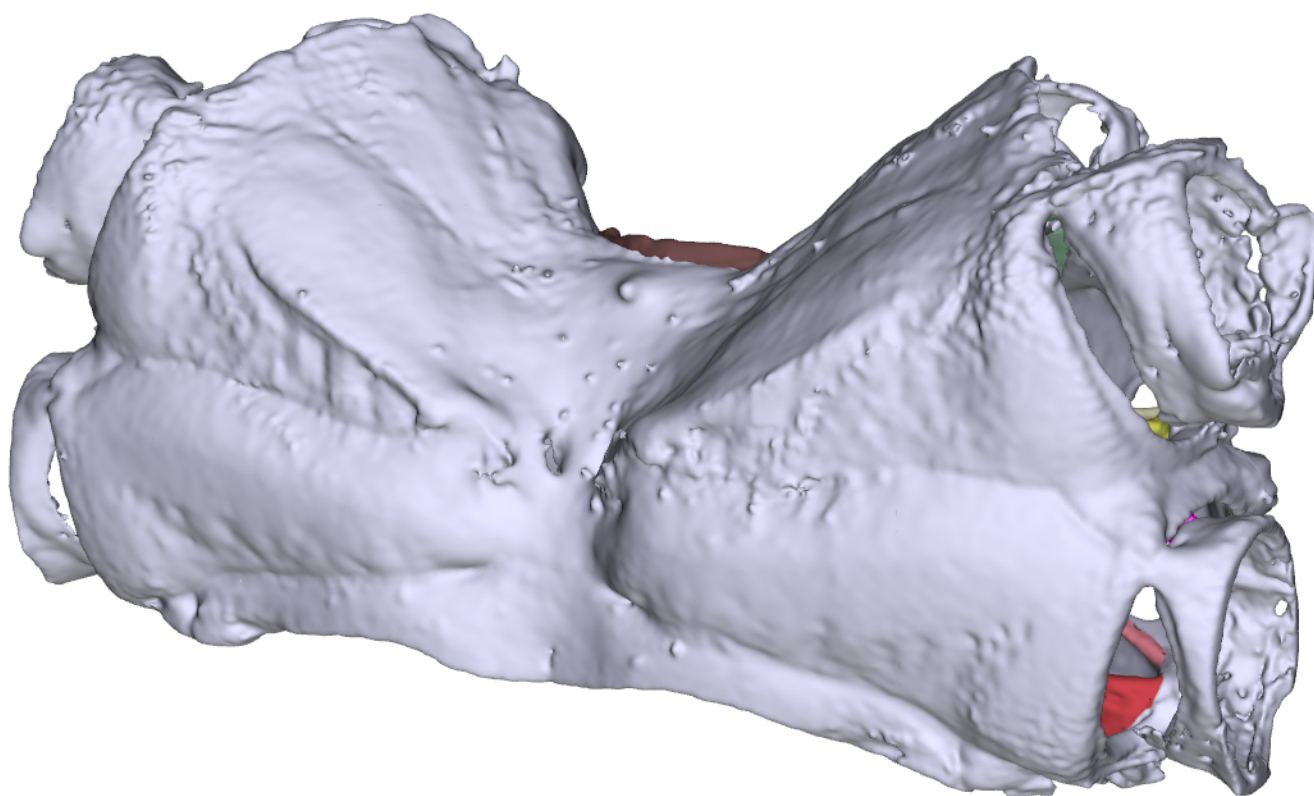

Supplement: Supplementary file 11 — Additional file 11. Three-dimensional (3D) model of Medorippe lanata showing the axial skeleton, proximal podomeres of thoracomeres 4–8, some P2 and P4 extrinsic muscles and P5 extrinsic musculature. [file 12983_2022_467_MOESM11_ESM.pdf]

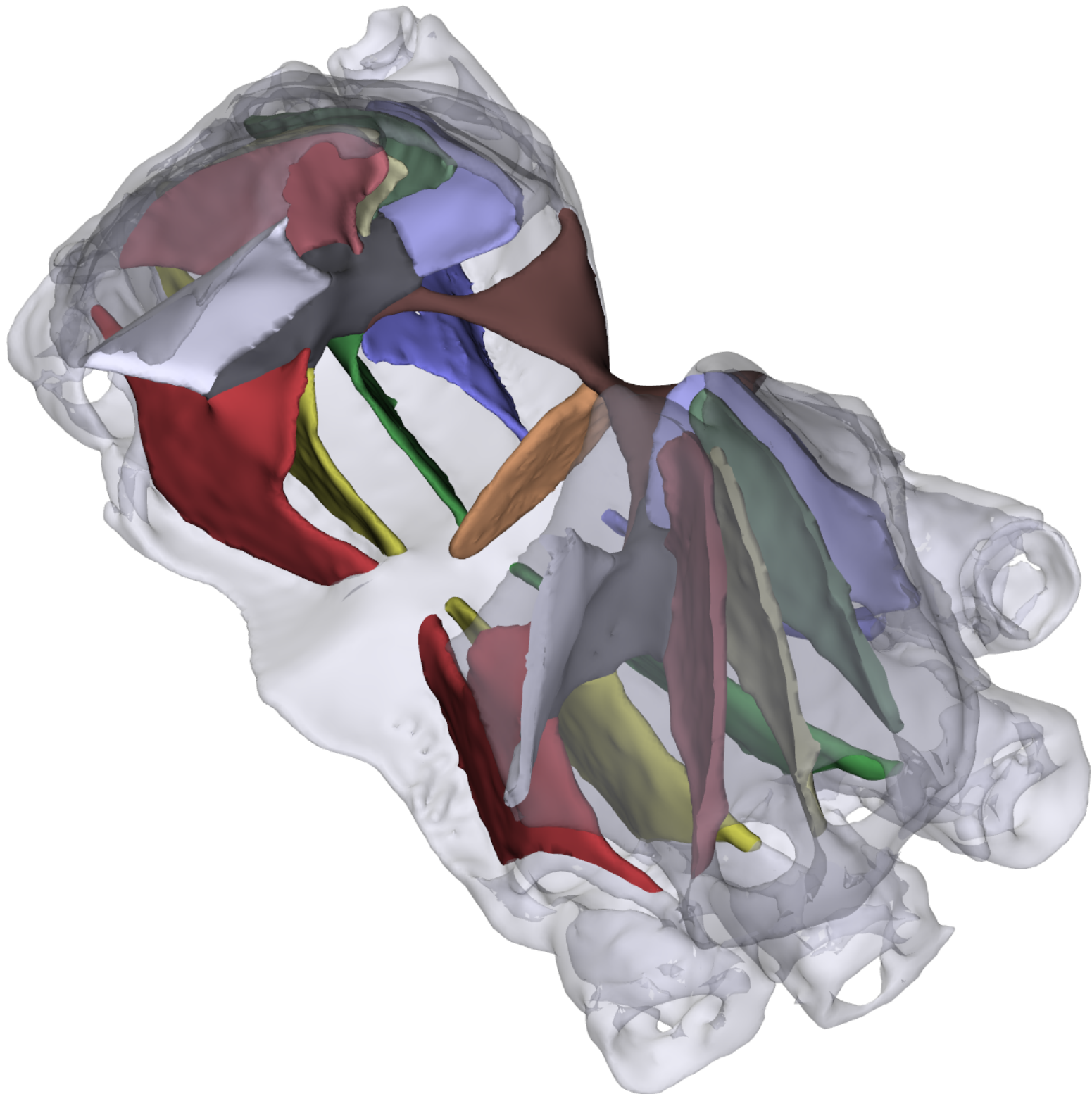

Supplement: Supplementary file 12 — Additional file 12. Three-dimensional (3D) model of Bathynectes maravigna showing the axial skeleton, proximal podomeres of thoracomeres 4–8 and P5 extrinsic musculature. Use model hierarchy to show extrinsic musculature. [file 12983_2022_467_MOESM12_ESM.pdf]

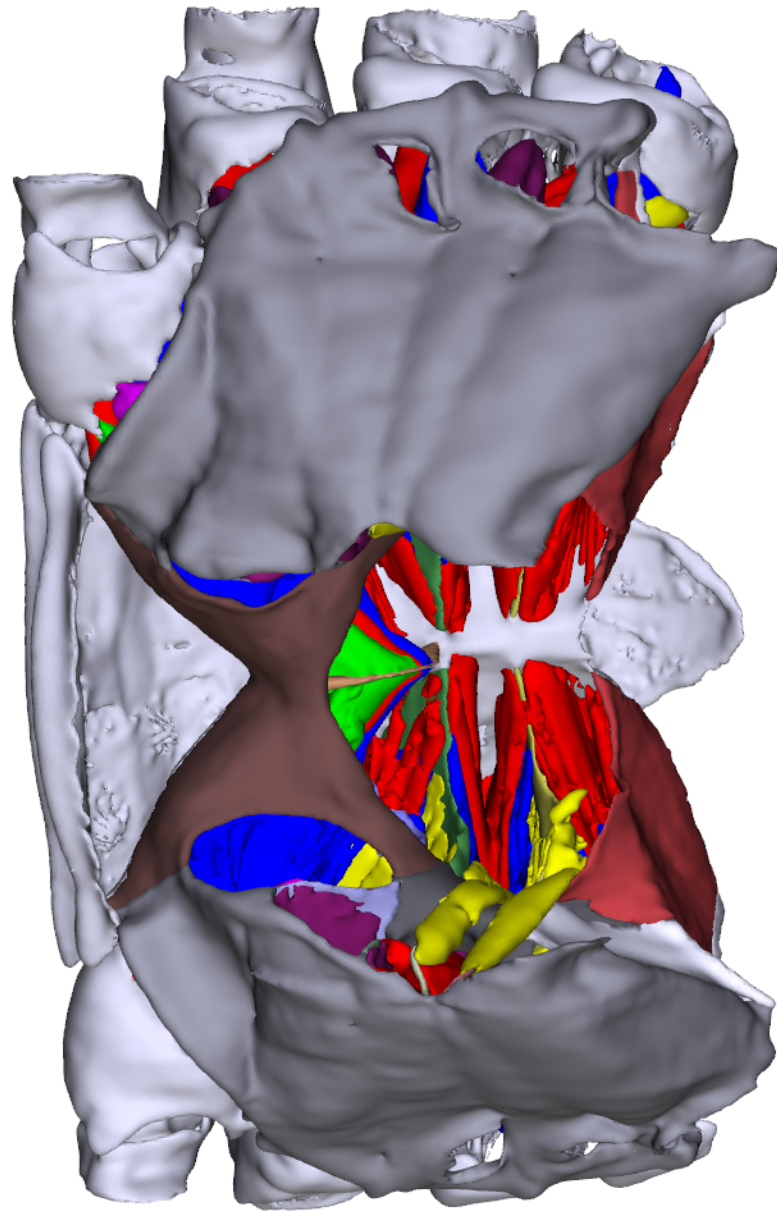

Supplement: Supplementary file 13 — Additional file 13. Three-dimensional (3D) model of Carcinus maenas showing the axial skeleton, proximal podomeres of thoracomeres 4–8, P5 intrinsic basi-ischium muscles and P2–P5 extrinsic musculature. [file 12983_2022_467_MOESM13_ESM.pdf]

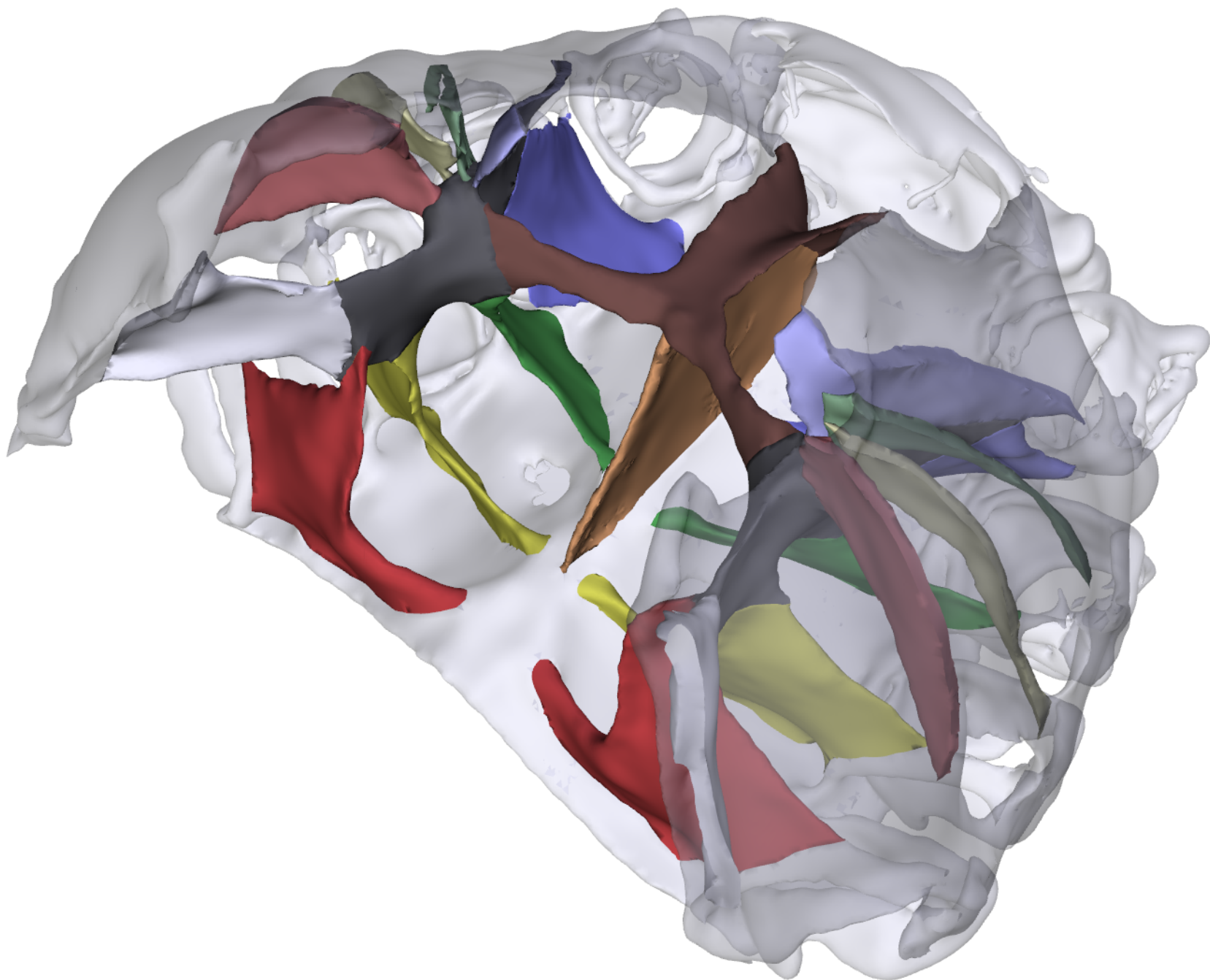

Supplement: Supplementary file 14 — Additional file 14. Three-dimensional (3D) model of Coelocarcinus foliatus showing the axial skeleton, proximal podomeres of thoracomeres 4–8, P4 extrinsic basi-ischium muscles and P5 extrinsic musculature. Use model hierarchy to show extrinsic musculature. [file 12983_2022_467_MOESM14_ESM.pdf]

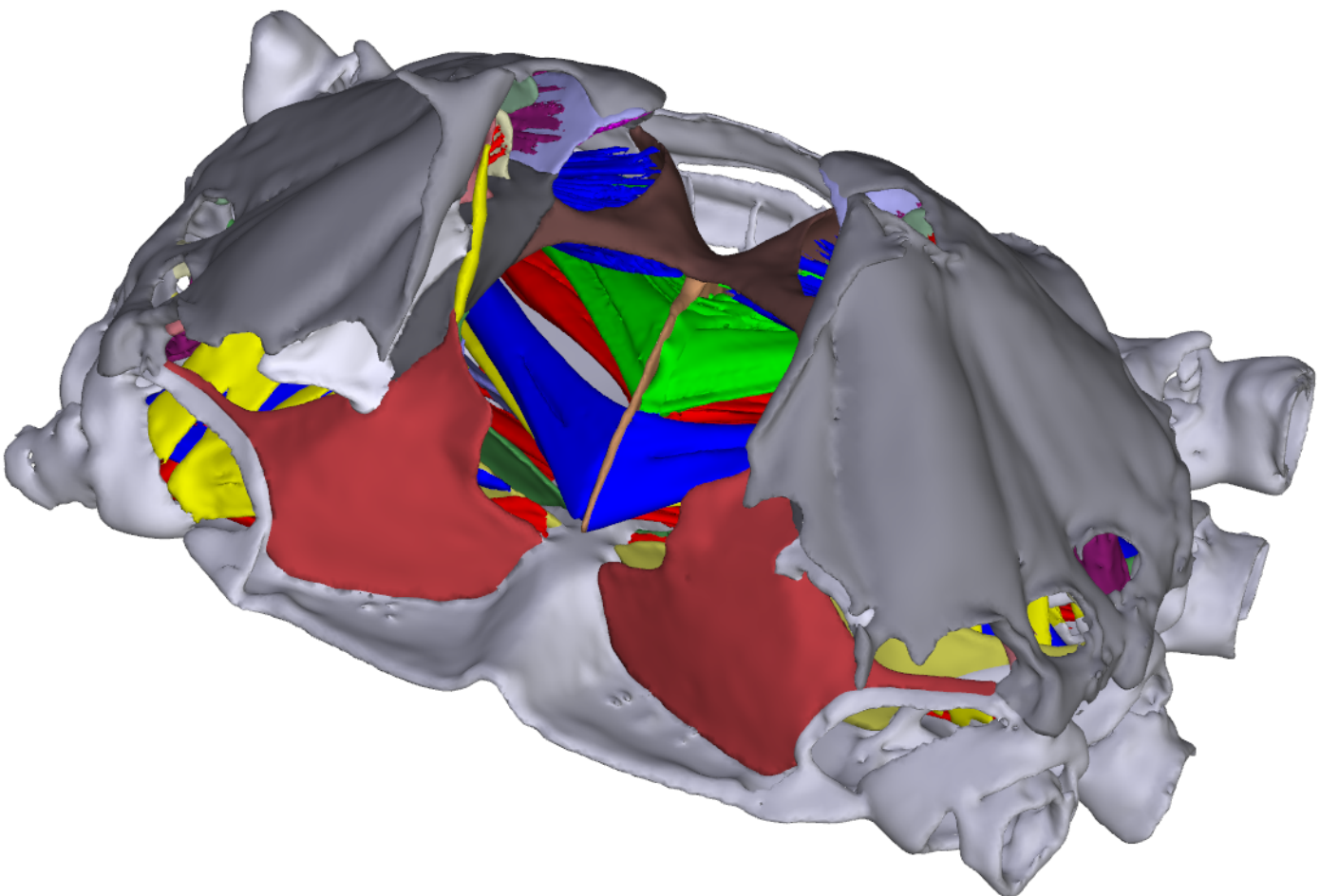

Supplement: Supplementary file 15 — Additional file 15. Three-dimensional (3D) model of Liocarcinus depurator showing the axial skeleton, proximal podomeres of thoracomeres 4–8 and P2–P5 extrinsic musculature. [file 12983_2022_467_MOESM15_ESM.pdf]

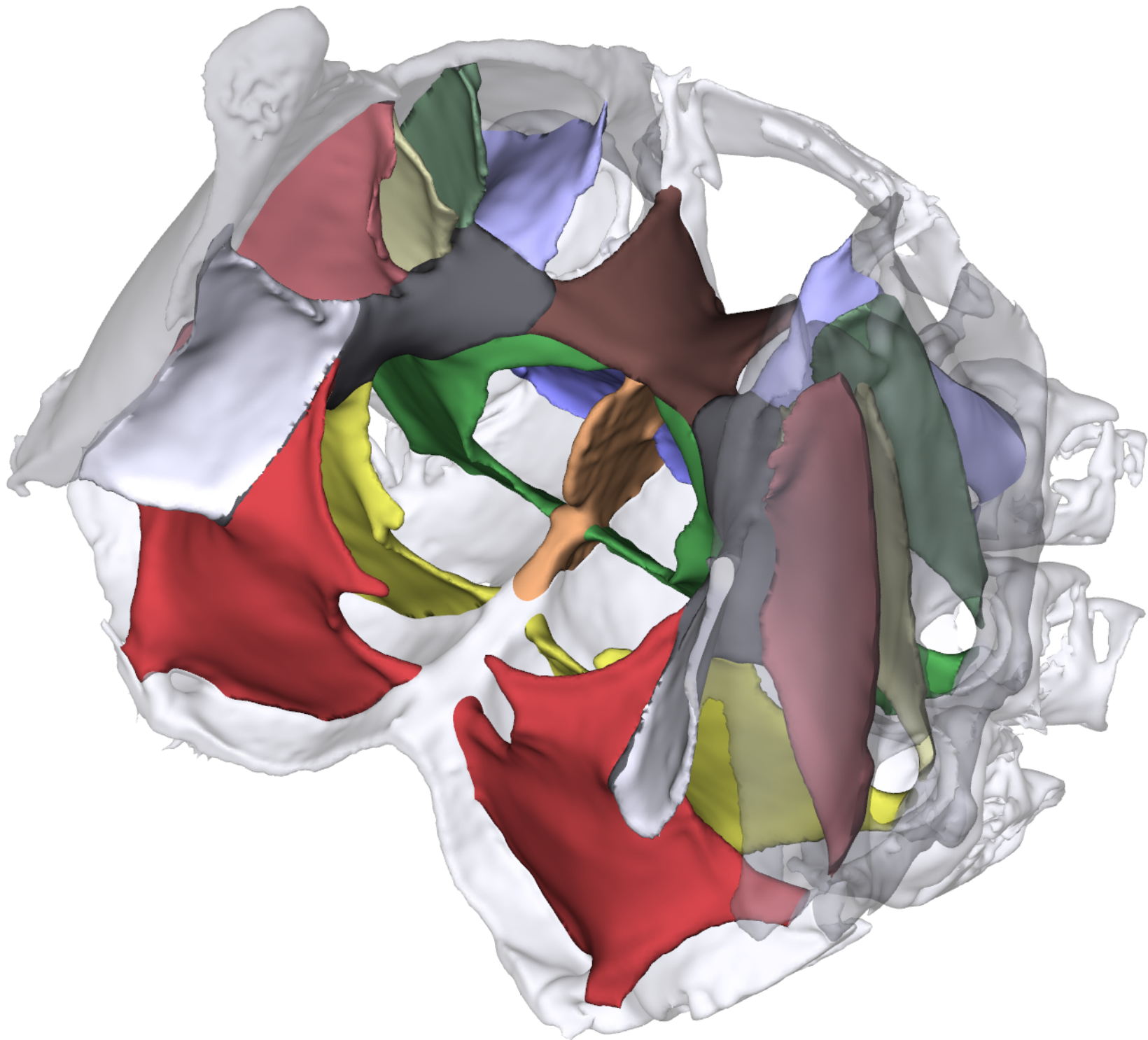

Supplement: Supplementary file 16 — Additional file 16. Three-dimensional (3D) model of Pirimela denticulata showing the axial skeleton, proximal podomeres of thoracomeres 4–8 and P5 extrinsic musculature. Use model hierarchy to show extrinsic musculature. [file 12983_2022_467_MOESM16_ESM.pdf]

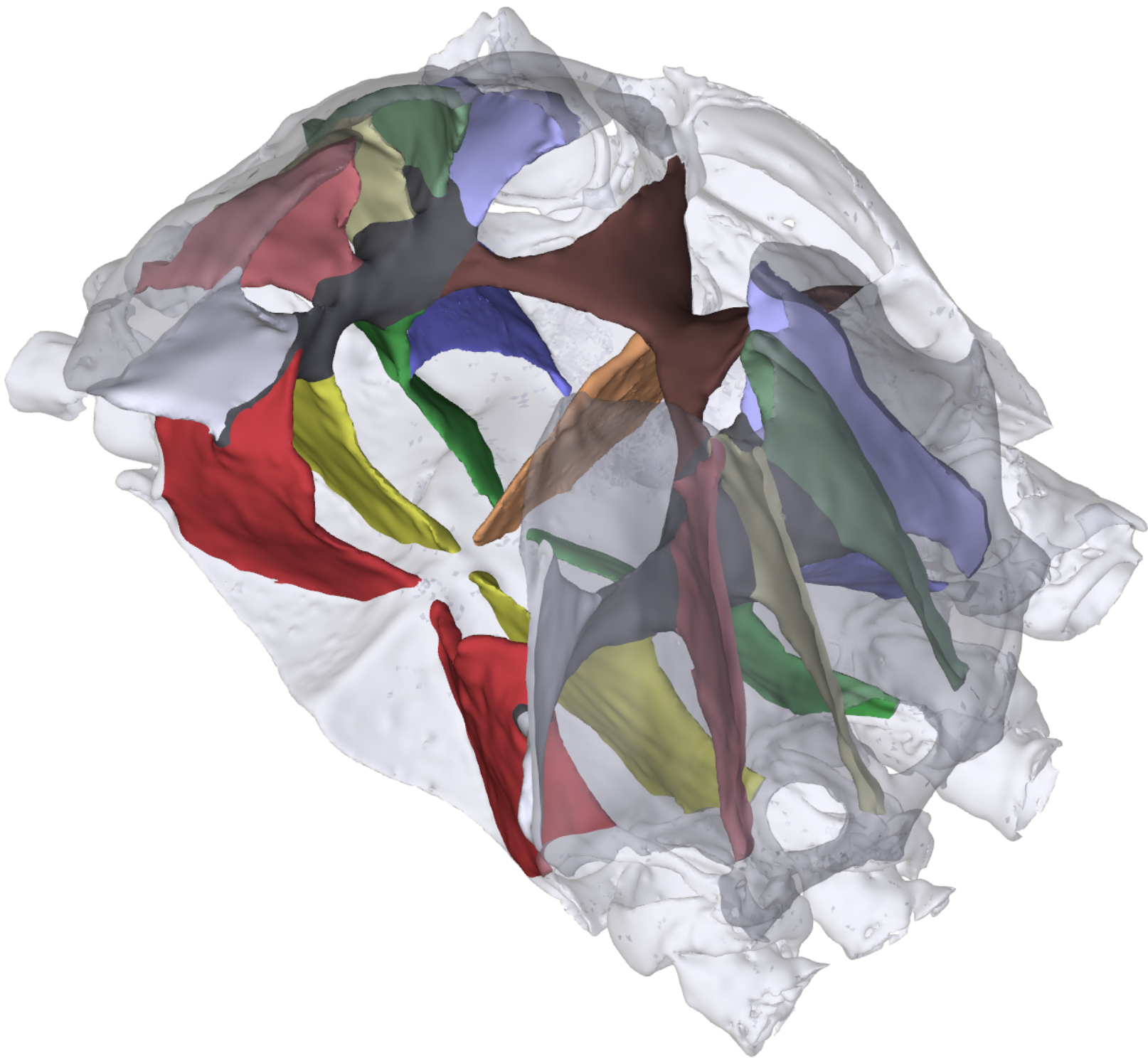

Supplement: Supplementary file 17 — Additional file 17. Three-dimensional (3D) model of “Polybius” henslowii showing the axial skeleton, proximal podomeres of thoracomeres 4–8, P5 intrinsic basi-ischium muscles and P5 extrinsic musculature. Use model hierarchy to show musculature. [file 12983_2022_467_MOESM17_ESM.pdf]

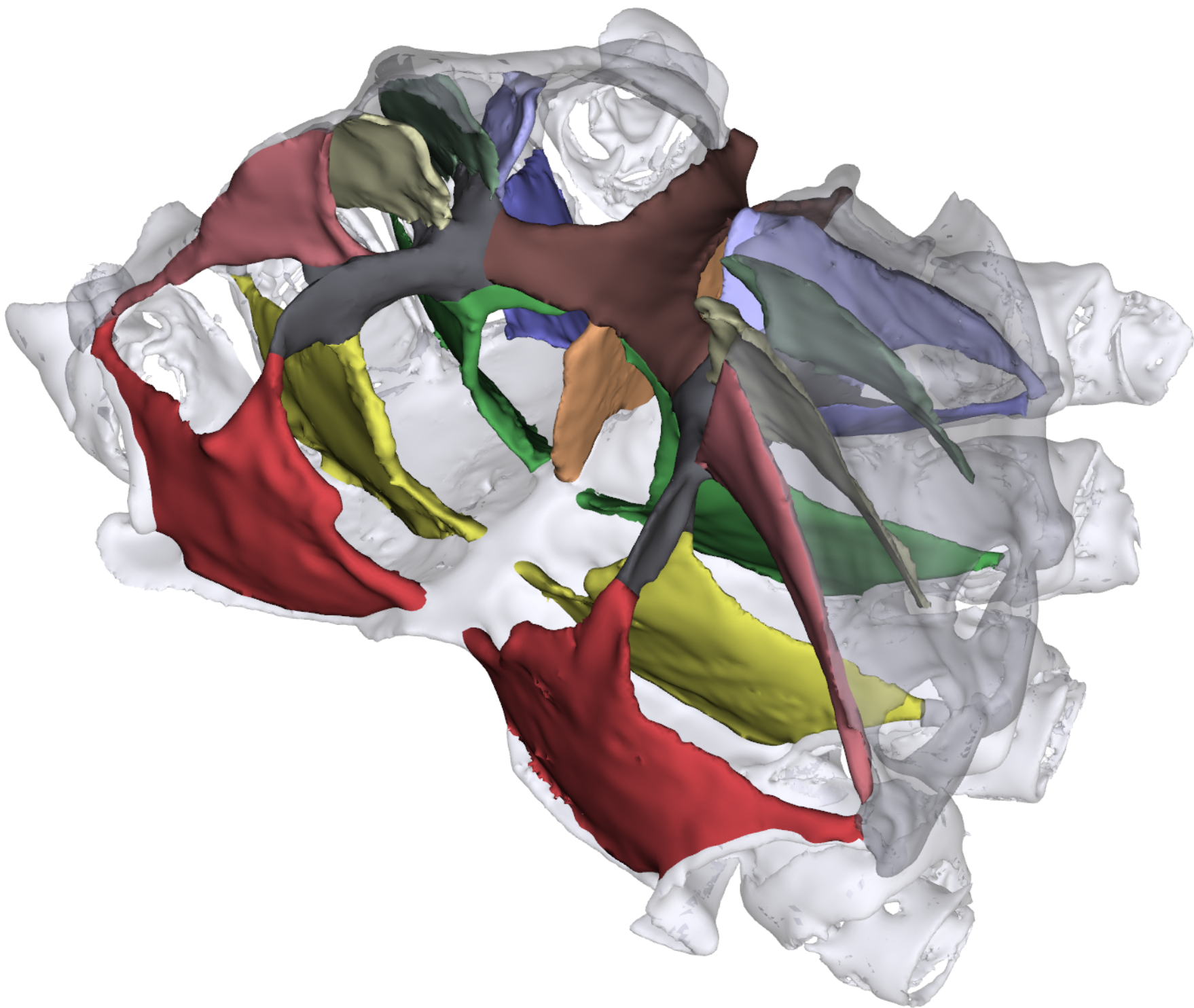

Supplement: Supplementary file 18 — Additional file 18. Three-dimensional (3D) model of Portumnus latipes showing the axial skeleton, proximal podomeres of thoracomeres 4–8 and P2–P5 extrinsic musculature. Use model hierarchy to show extrinsic musculature. [file 12983_2022_467_MOESM18_ESM.pdf]

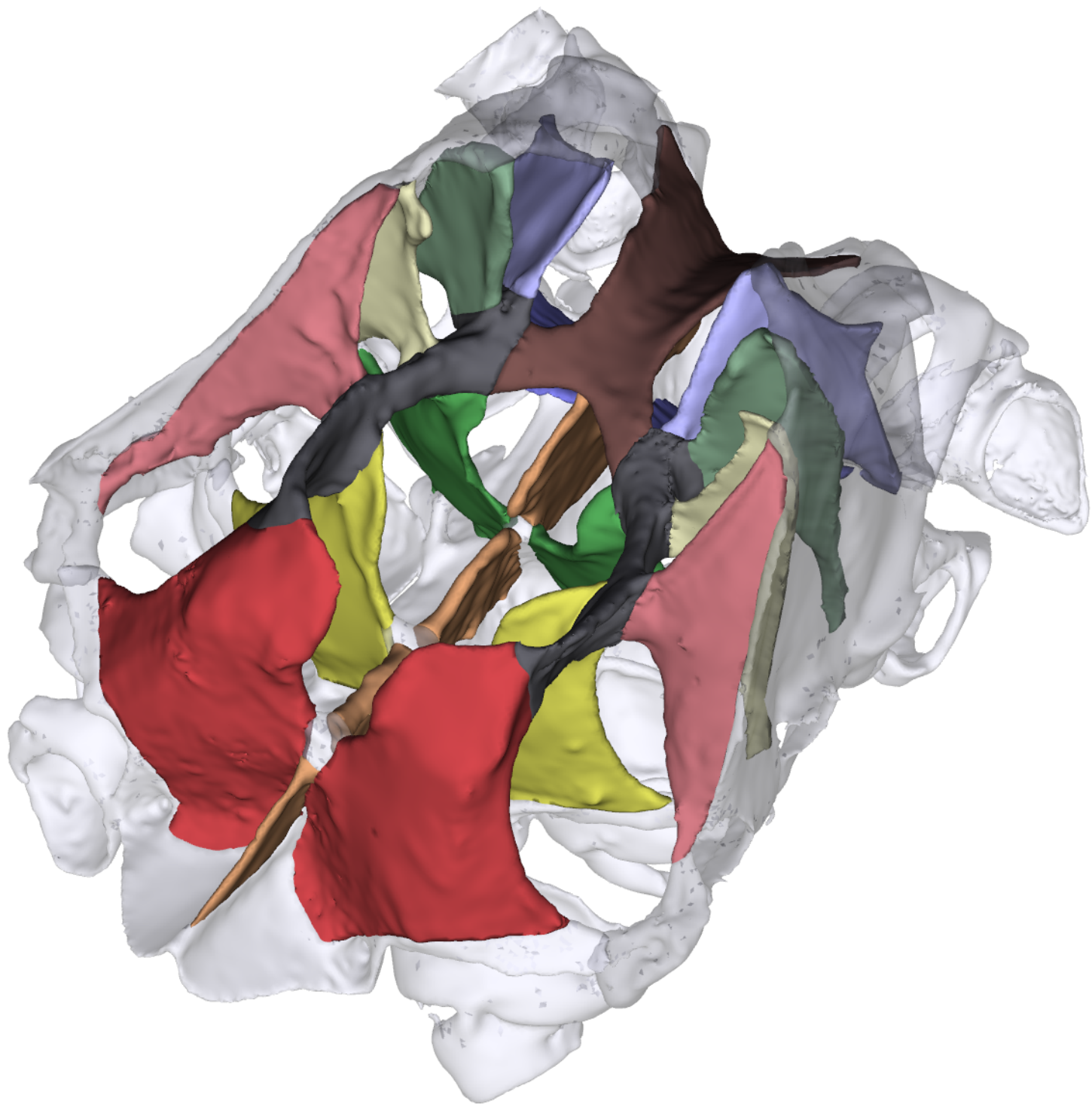

Supplement: Supplementary file 19 — Additional file 19. Three-dimensional (3D) model of Thia scutellata showing the axial skeleton, proximal podomeres of thoracomeres 4–8, P5 intrinsic basi-ischium muscles and P2–P5 extrinsic musculature. Use model hierarchy to show musculature. [file 12983_2022_467_MOESM19_ESM.pdf]

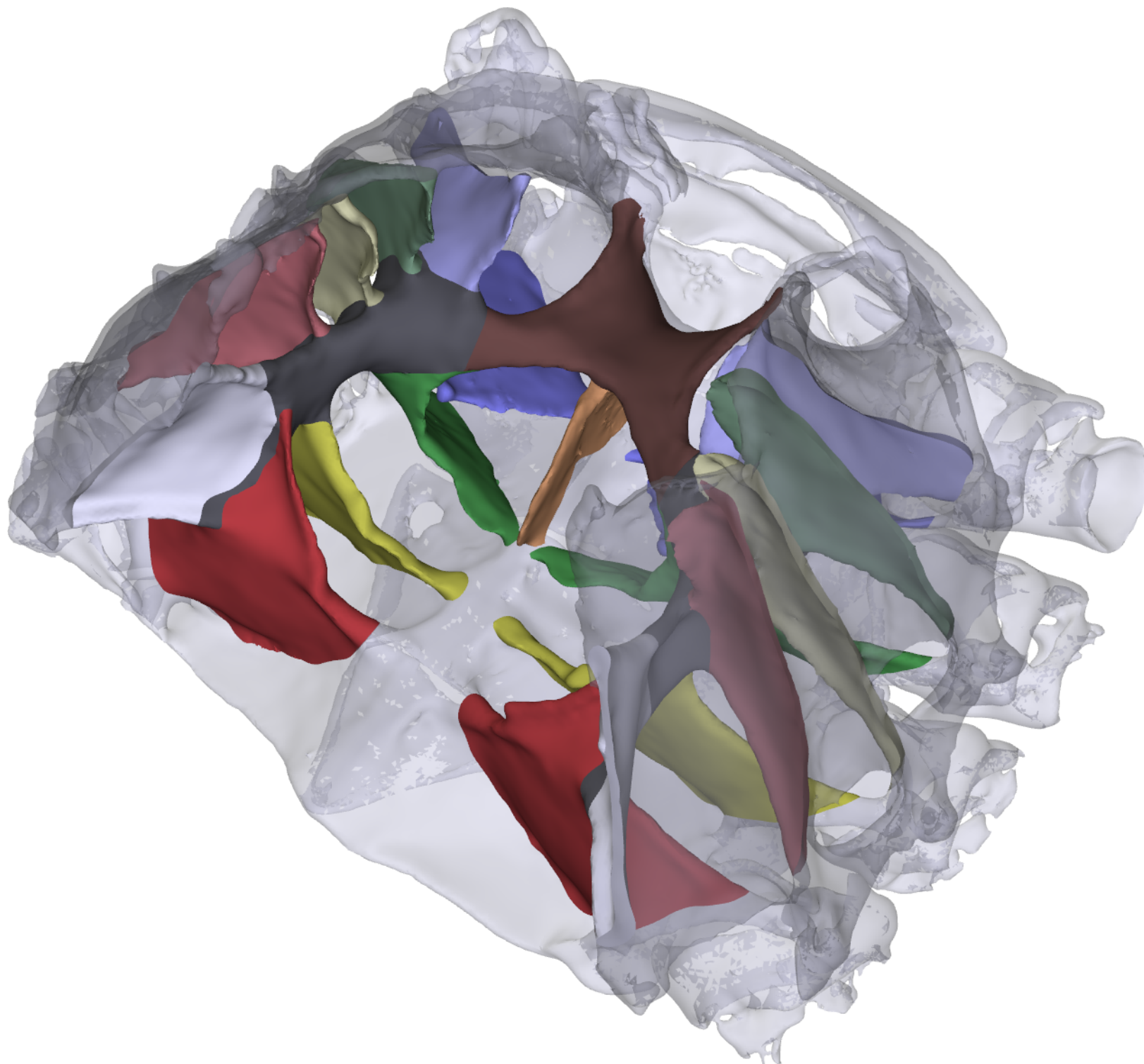

Supplement: Supplementary file 20 — Additional file 20. Three-dimensional (3D) model of Xaiva biguttata showing the axial skeleton, proximal podomeres of thoracomeres 4–8, P5 intrinsic basi-ischium muscles and P5 extrinsic musculature. Use model hierarchy to show musculature. [file 12983_2022_467_MOESM20_ESM.pdf]

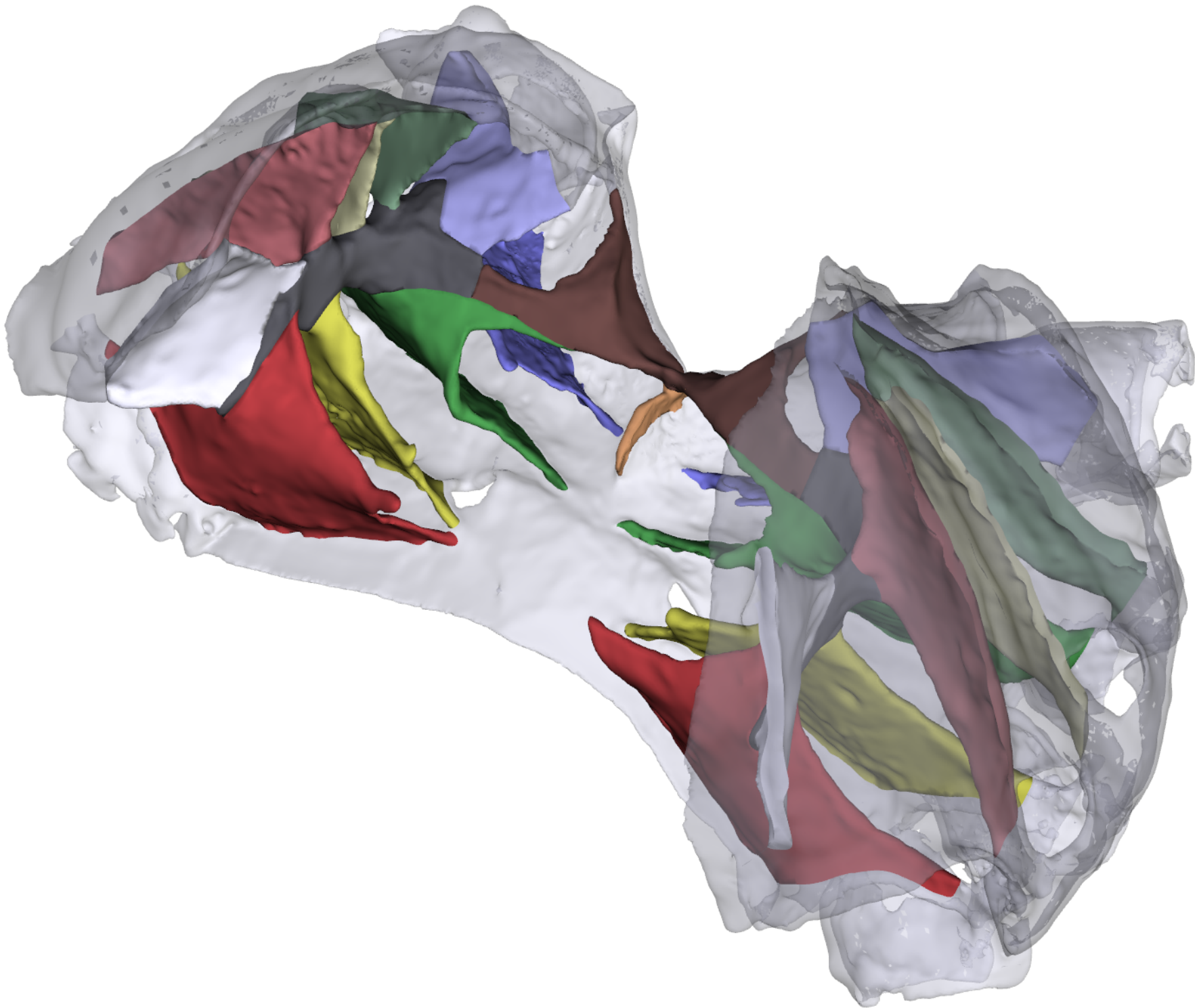

Supplement: Supplementary file 21 — Additional file 21. Three-dimensional (3D) model of Chaceon mediterraneus showing the axial skeleton, proximal podomeres of thoracomeres 4–8 and P5 extrinsic musculature. Use model hierarchy to show extrinsic musculature. [file 12983_2022_467_MOESM21_ESM.pdf]

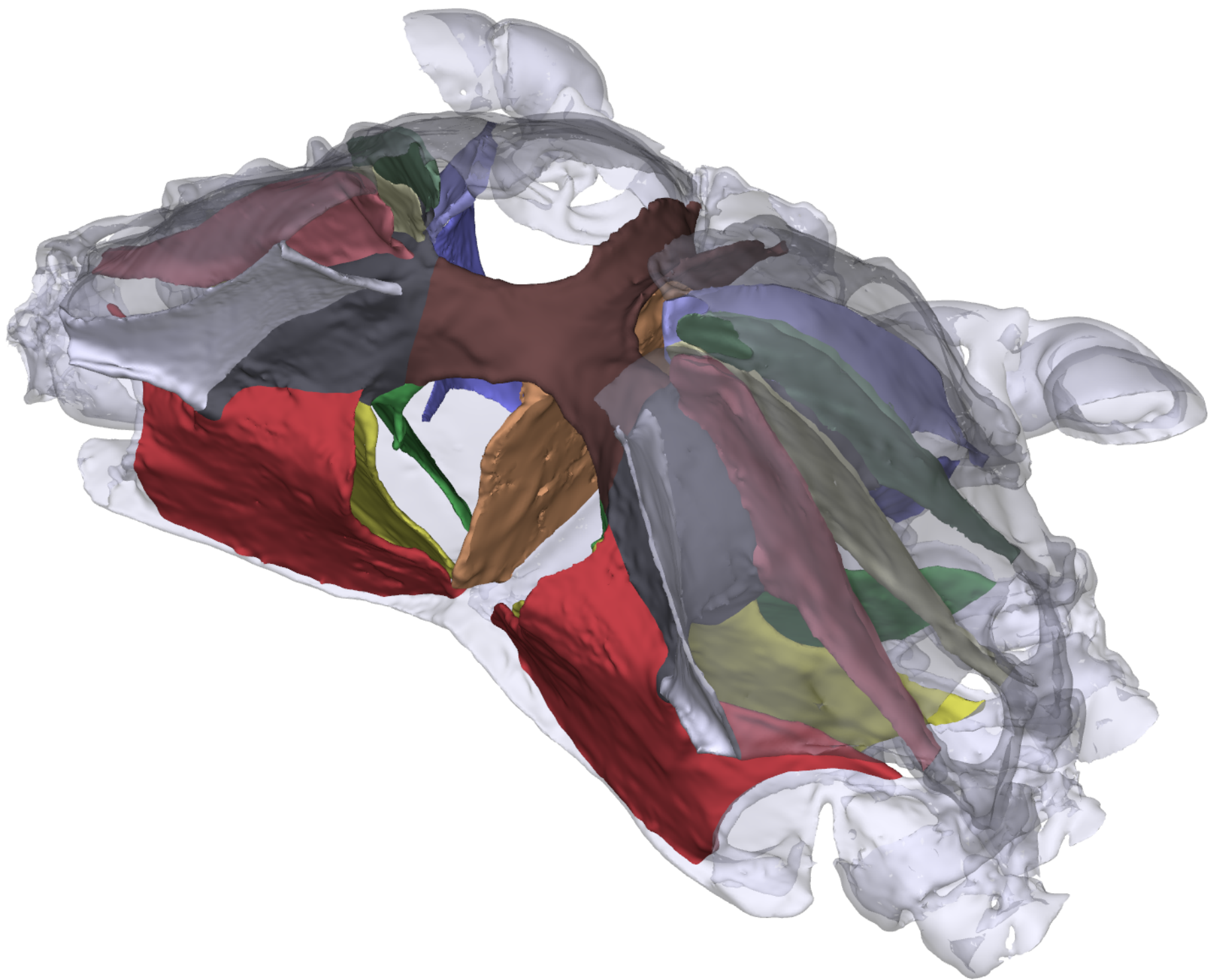

Supplement: Supplementary file 22 — Additional file 22. Three-dimensional (3D) model of Ovalipes ocellatus showing the axial skeleton, proximal podomeres of thoracomeres 4–8, some P2–P4 extrinsic muscles and P5 extrinsic musculature. Use model hierarchy to show extrinsic musculature. [file 12983_2022_467_MOESM22_ESM.pdf]

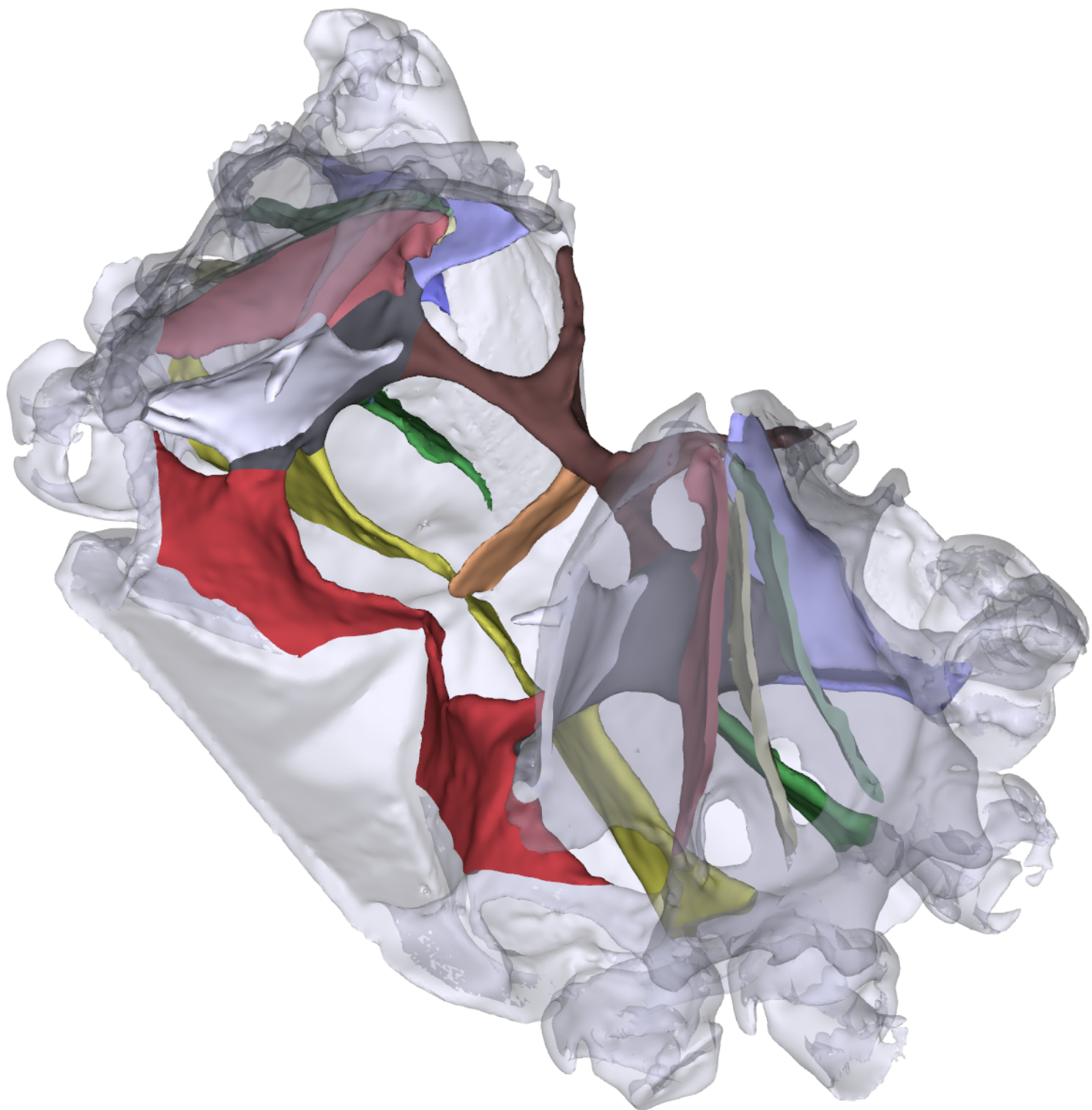

Supplement: Supplementary file 23 — Additional file 23. Three-dimensional (3D) model of Caphyra rotundifrons showing the axial skeleton, proximal podomeres of thoracomeres 4–8, P5 intrinsic basi-ischium muscles and P5 extrinsic musculature. Use model hierarchy to show musculature. [file 12983_2022_467_MOESM23_ESM.pdf]

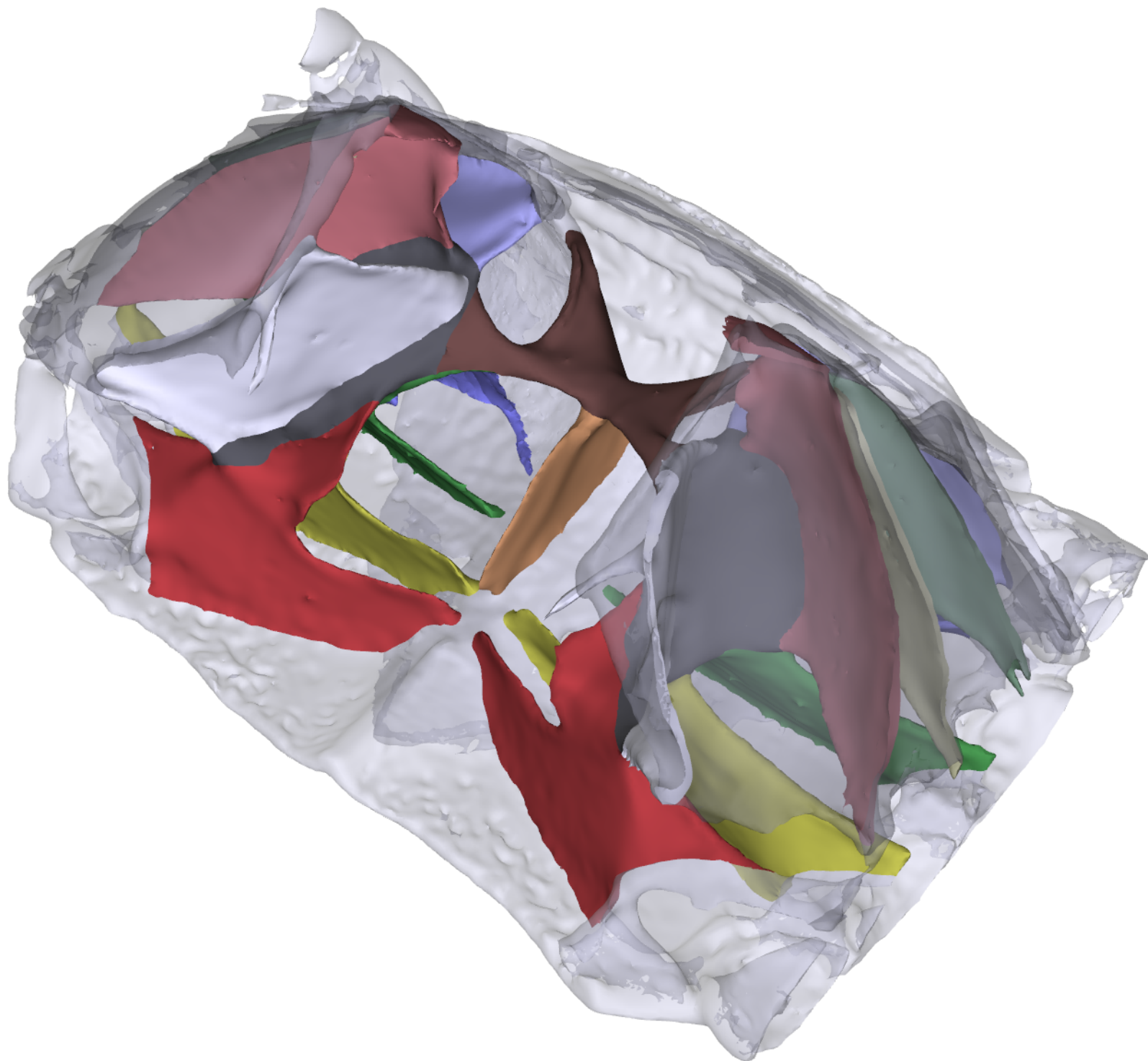

Supplement: Supplementary file 24 — Additional file 24. Three-dimensional (3D) model of Carupa tenuipes showing the axial skeleton, proximal podomeres of thoracomeres 4–8 and P5 extrinsic musculature. Use model hierarchy to show extrinsic musculature. [file 12983_2022_467_MOESM24_ESM.pdf]

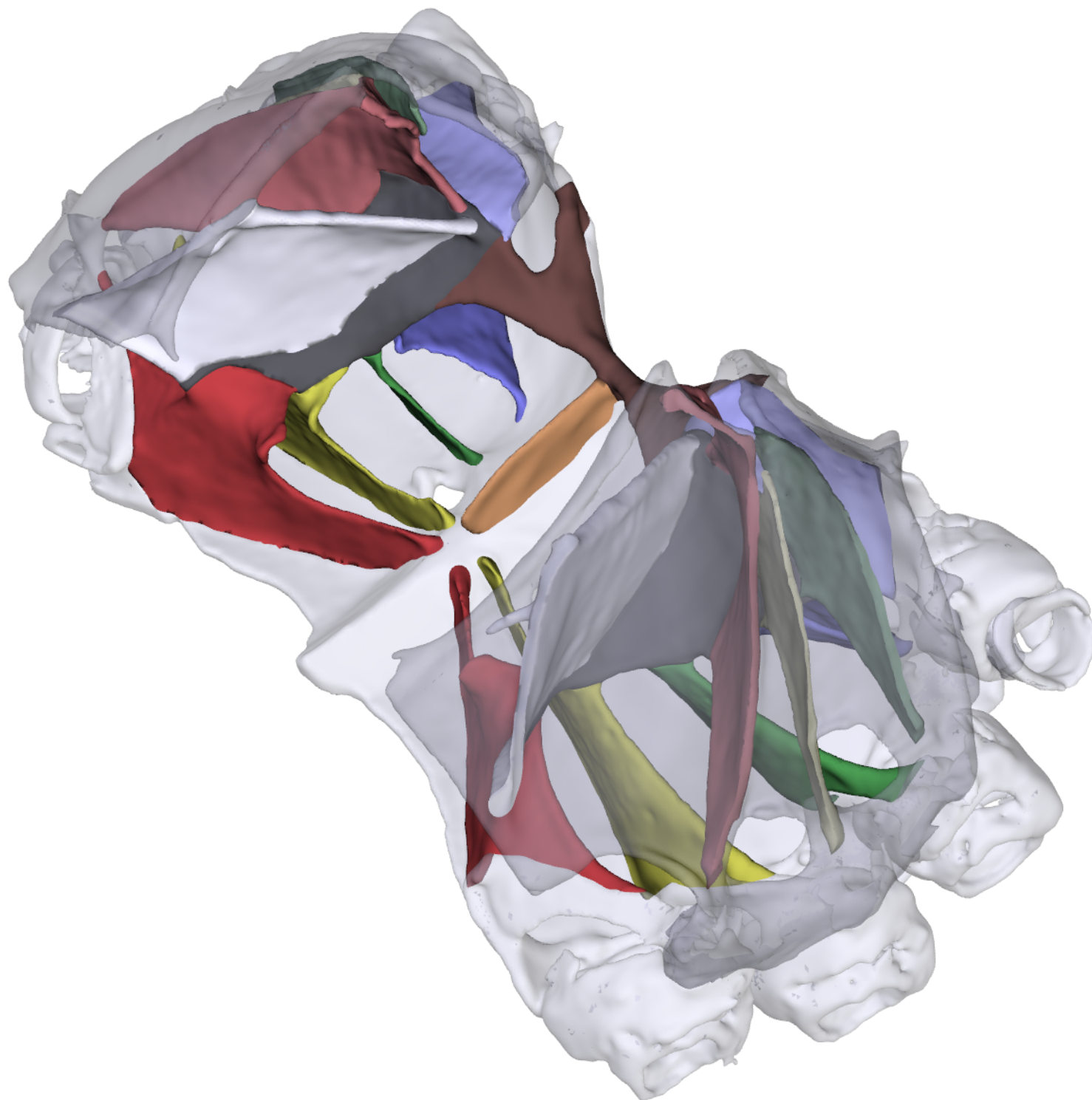

Supplement: Supplementary file 25 — Additional file 25. Three-dimensional (3D) model of Catoptrus nitidus showing the axial skeleton, proximal podomeres of thoracomeres 4–8 and P5 extrinsic musculature. Use model hierarchy to show extrinsic musculature. [file 12983_2022_467_MOESM25_ESM.pdf]

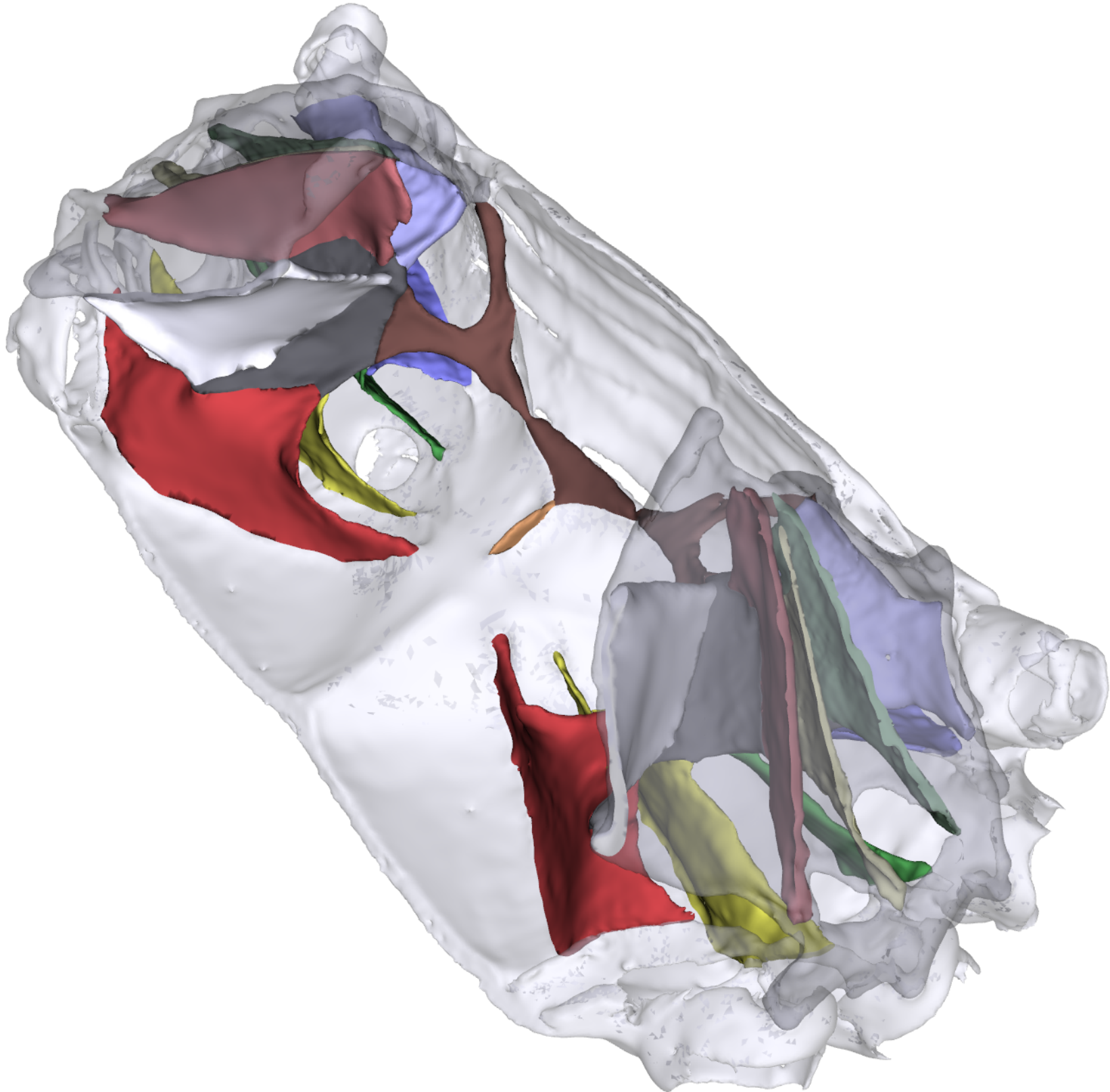

Supplement: Supplementary file 26 — Additional file 26. Three-dimensional (3D) model of Libystes nitidus showing the axial skeleton, proximal podomeres of thoracomeres 4–8, some P2–P4 extrinsic muscles, some P5 intrinsic muscles and P5 extrinsic musculature. Use model hierarchy to show musculature. [file 12983_2022_467_MOESM26_ESM.pdf]

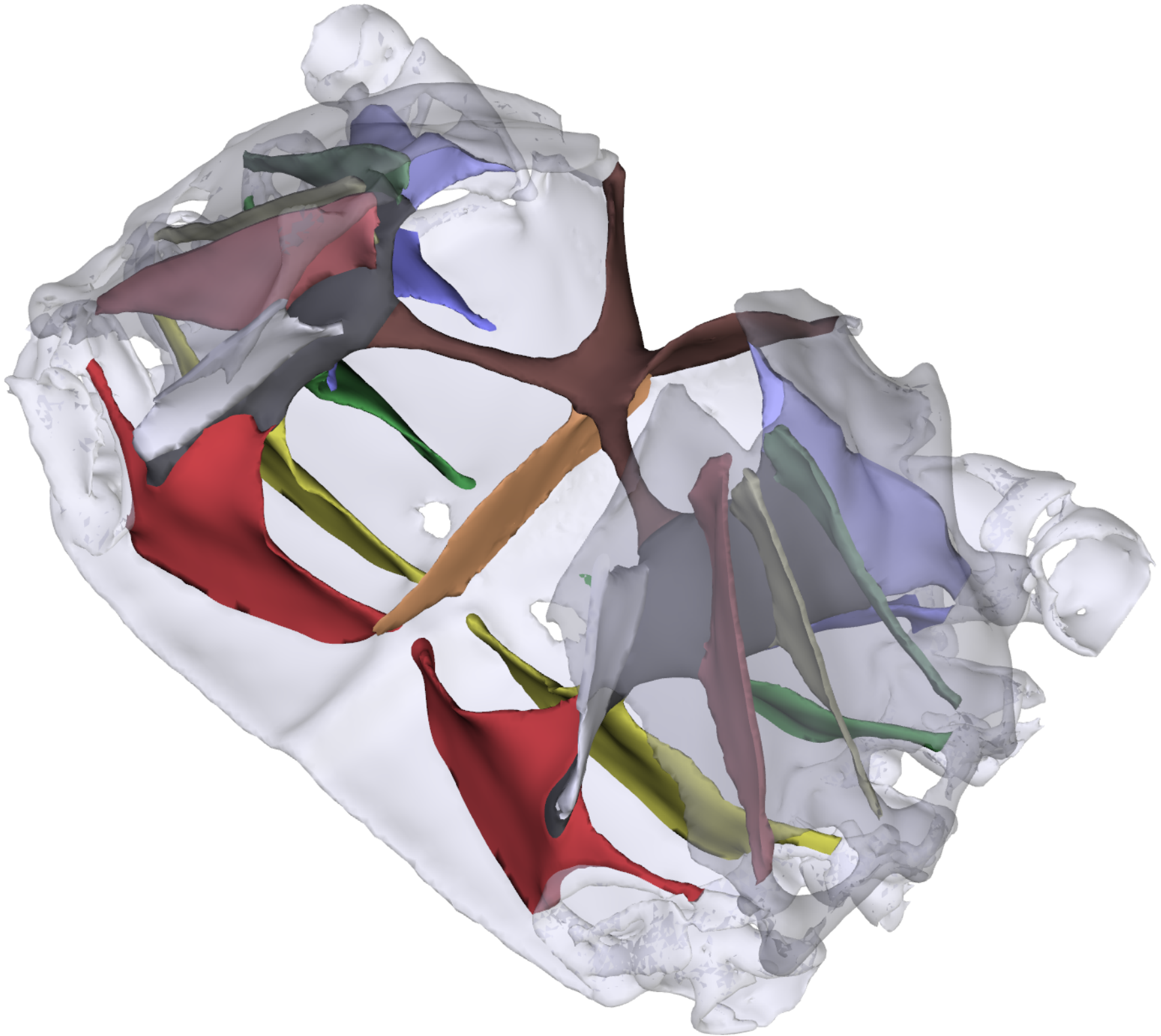

Supplement: Supplementary file 27 — Additional file 27. Three-dimensional (3D) model of Portunus inaequalis showing the axial skeleton, proximal podomeres of thoracomeres 4–8 and P5 extrinsic musculature. Use model hierarchy to show extrinsic musculature. [file 12983_2022_467_MOESM27_ESM.pdf]

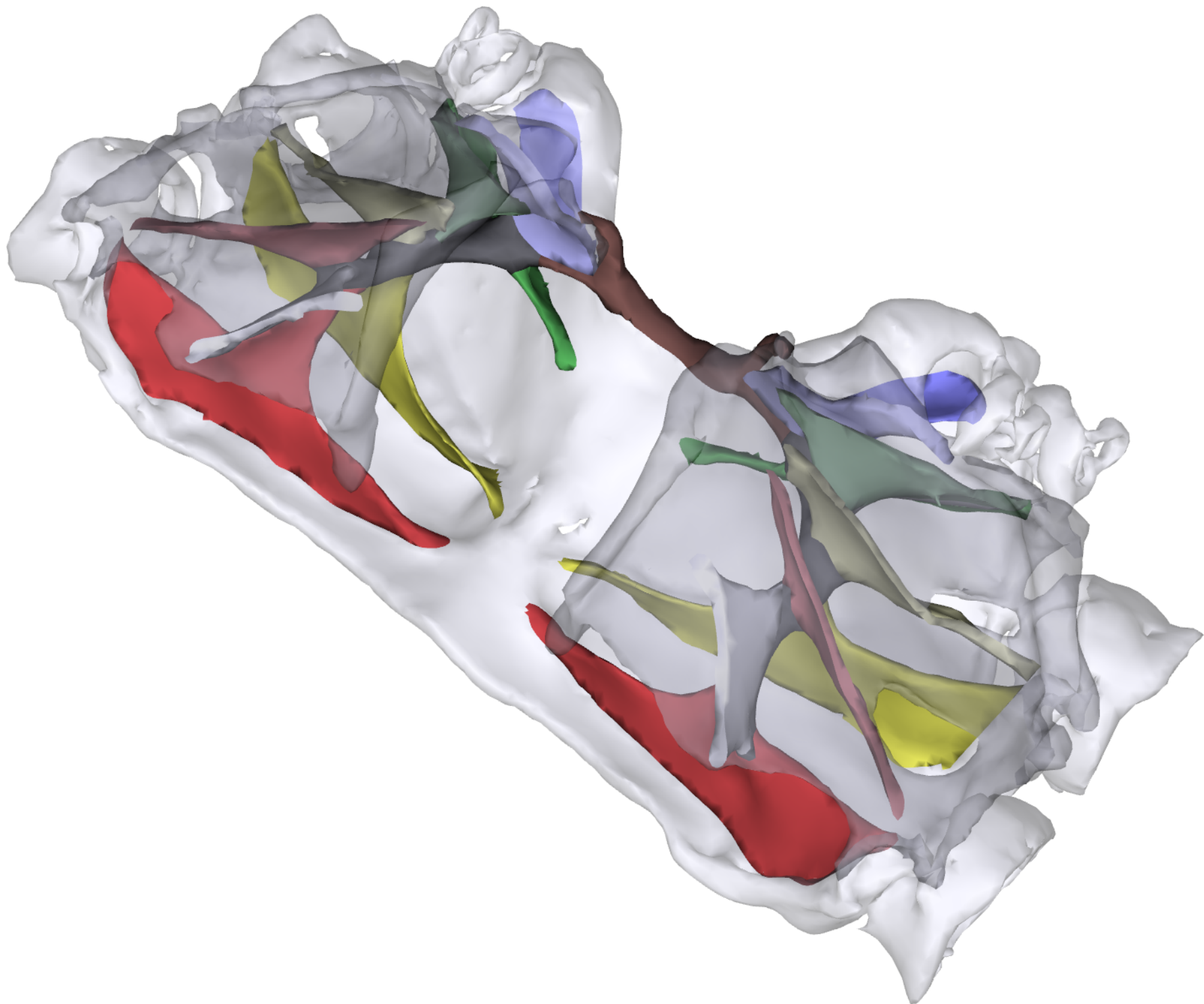

Supplement: Supplementary file 28 — Additional file 28. Low-resolution three-dimensional (3D) model of Medorippe lanata showing the axial skeleton and proximal podomeres of thoracomeres 4–8. [file 12983_2022_467_MOESM28_ESM.pdf]

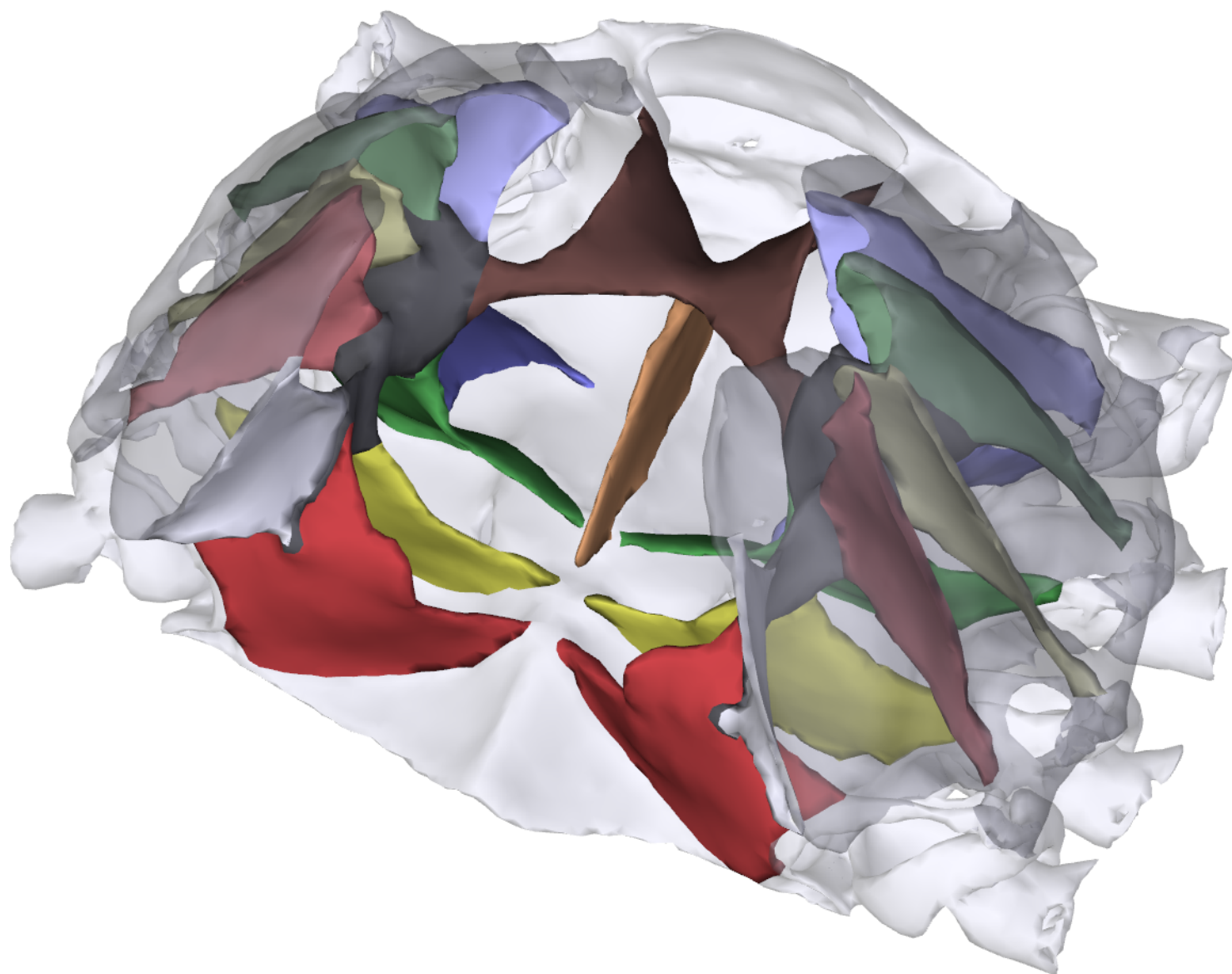

Supplement: Supplementary file 29 — Additional file 29. Low-resolution three-dimensional (3D) model of “Polybius” henslowii showing the axial skeleton and proximal podomeres of thoracomeres 4–8. [file 12983_2022_467_MOESM29_ESM.pdf]

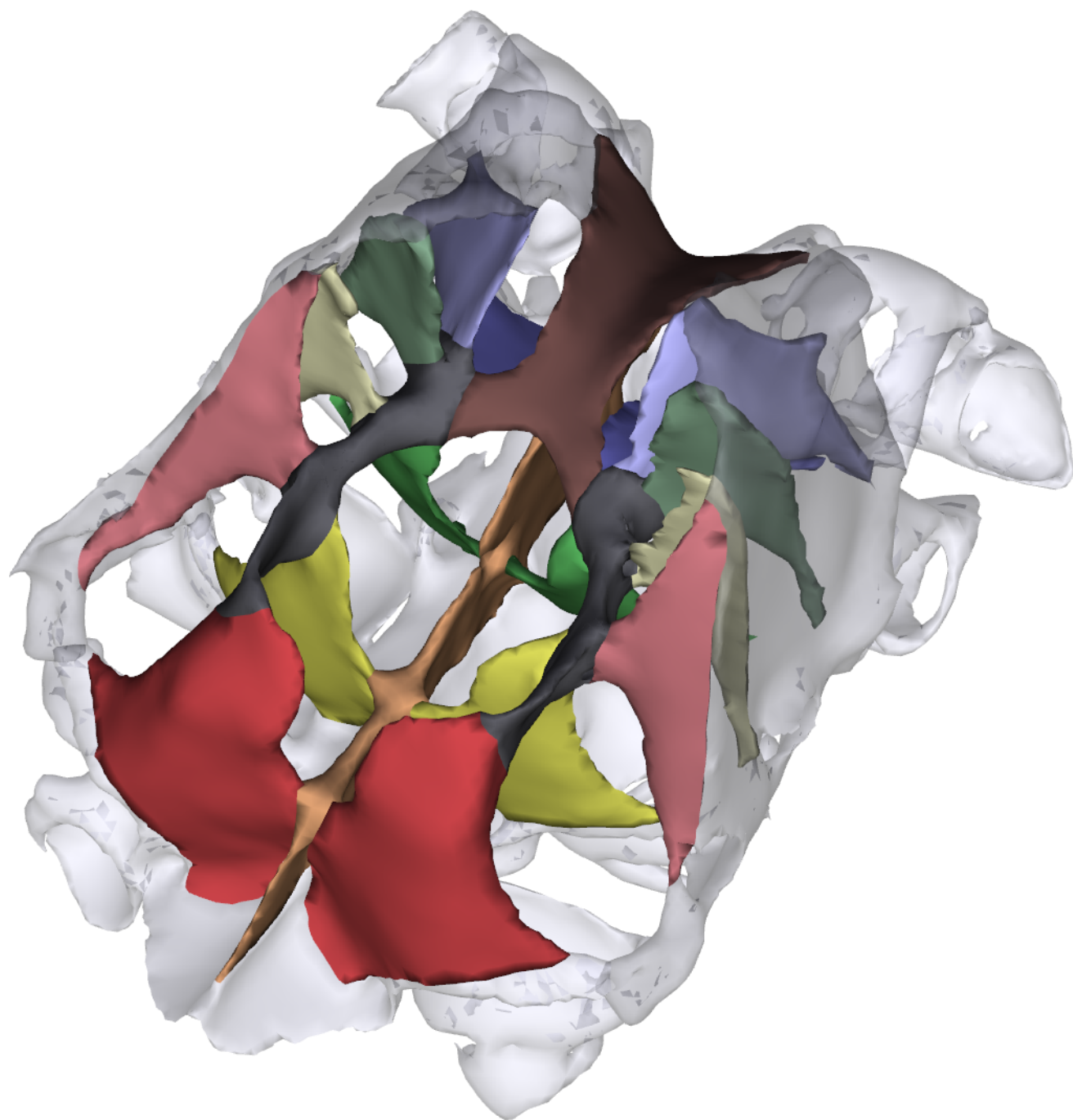

Supplement: Supplementary file 30 — Additional file 30. Low-resolution three-dimensional (3D) model of Thia scutellata showing the axial skeleton and proximal podomeres of thoracomeres 4–8. [file 12983_2022_467_MOESM30_ESM.pdf]

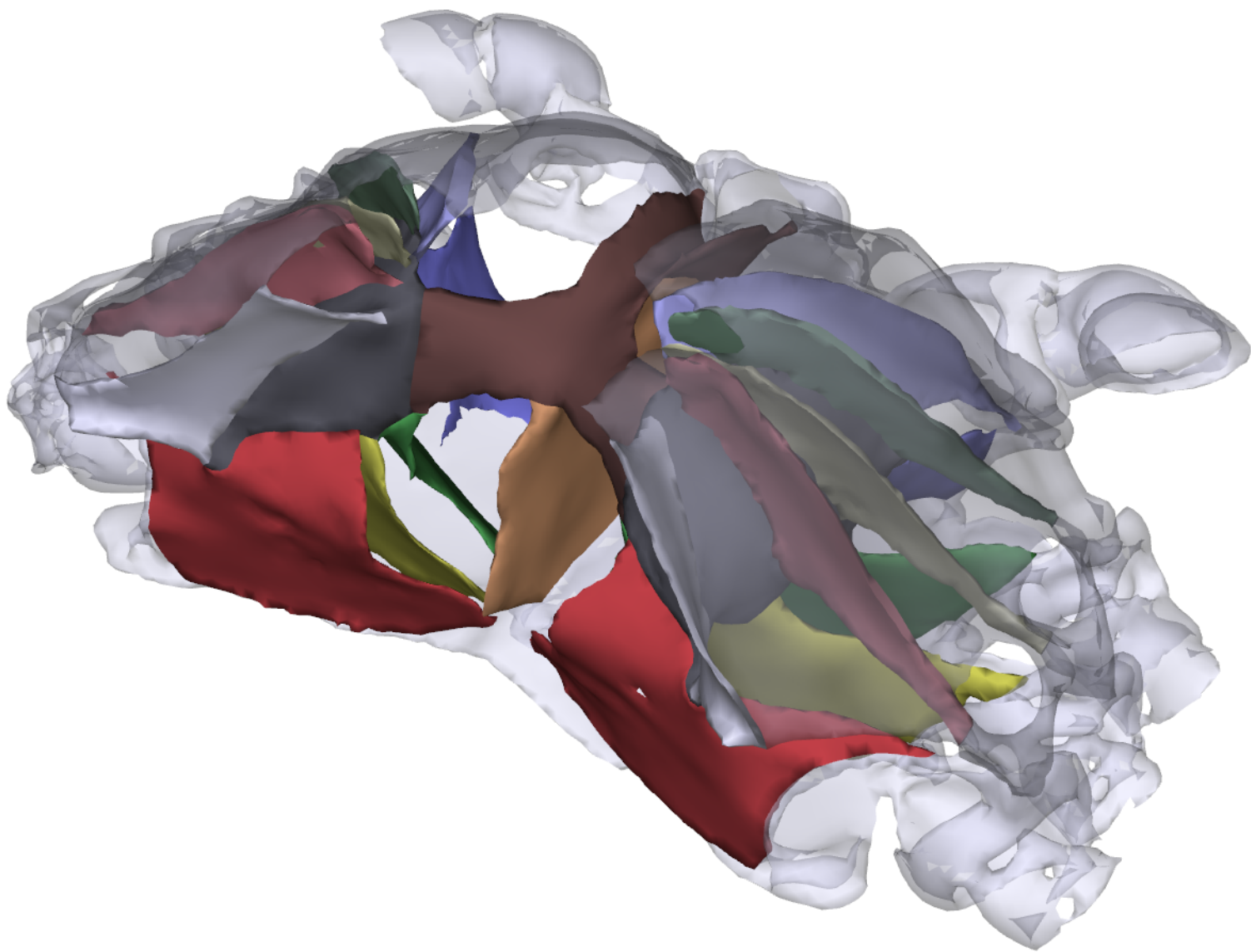

Supplement: Supplementary file 31 — Additional file 31. Low-resolution three-dimensional (3D) model of Ovalipes ocellatus showing the axial skeleton and proximal podomeres of thoracomeres 4–8. [file 12983_2022_467_MOESM31_ESM.pdf]

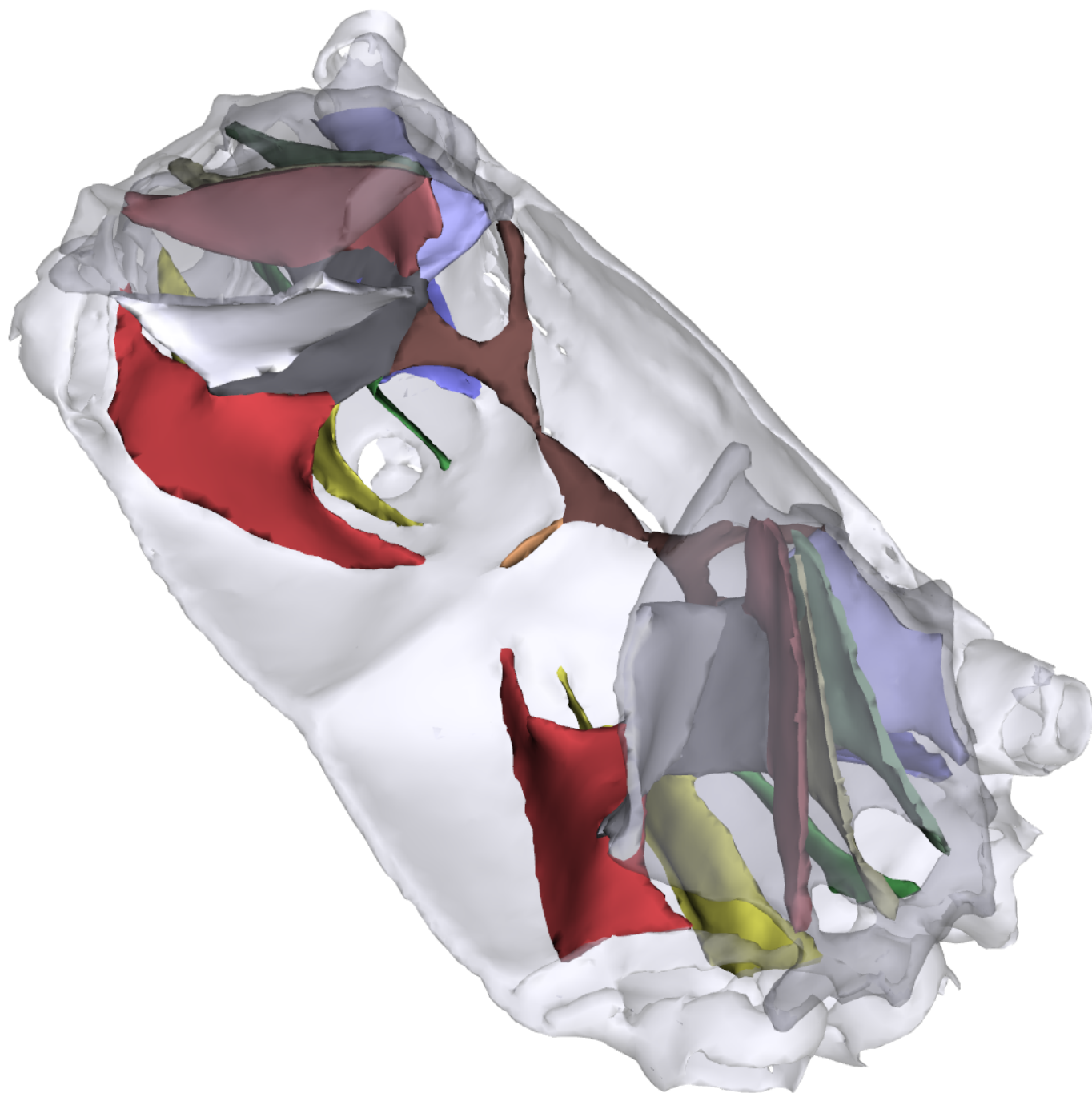

Supplement: Supplementary file 32 — Additional file 32. Low-resolution three-dimensional (3D) model of Libystes nitidus showing the axial skeleton and proximal podomeres of thoracomeres 4–8. [file 12983_2022_467_MOESM32_ESM.pdf]
